# Hybrid Polyketides from a *Hydractinia*-Associated *Cladosporium sphaerospermum* SW67 and Their Putative Biosynthetic Origin

**DOI:** 10.3390/md17110606

**Published:** 2019-10-24

**Authors:** Seoung Rak Lee, Dahae Lee, Hee Jeong Eom, Maja Rischer, Yoon-Joo Ko, Ki Sung Kang, Chung Sub Kim, Christine Beemelmanns, Ki Hyun Kim

**Affiliations:** 1 School of Pharmacy, Sungkyunkwan University, Suwon 16419, Korea; davidseoungrak@gmail.com (S.R.L.); pjsldh@naver.com (D.L.); itprthj44@gmail.com (H.J.E.); 2 Leibniz Institute for Natural Product Research and Infection Biology e.V., Hans-Knöll-Institute (HKI), 07745 Jena, Germany; Maja.Rischer@hki-jena.de; 3 Laboratory of Nuclear Magnetic Resonance, National Center for Inter-University Research Facilities (NCIRF), Seoul National University, Gwanak-gu, Seoul 08826, Korea; yjko@snu.ac.kr; 4 College of Korean Medicine, Gachon University, Seongnam 13120, Korea; kkang@gachon.ac.kr; 5 Department of Chemistry, Yale University, New Haven, CT 06520, USA; chungsub.kim@yale.edu; 6 Chemical Biology Institute, Yale University, West Haven, CT 06516, USA

**Keywords:** hybrid polyketides, tetramic acid, *Cladosporium sphaerospermum*, hybrid PKS-NRPS, LLC-PK1 cells

## Abstract

Five hybrid polyketides (**1a**, **1b**, and **2**–**4**) containing tetramic acid core including a new hybrid polyketide, cladosin L (**1**), were isolated from the marine fungus *Cladosporium sphaerospermum* SW67, which was isolated from the marine hydroid polyp of *Hydractinia echinata*. The hybrid polyketides were isolated as a pair of interconverting geometric isomers. The structure of **1** was determined based on 1D and 2D NMR spectroscopic and HR-ESIMS analyses. Its absolute configuration was established by quantum chemical electronic circular dichroism (ECD) calculations and modified Mosher’s method. Tetramic acid-containing compounds are reported to be derived from a hybrid PKS-NRPS, which was also proved by analyzing our ^13^C-labeling data. We investigated whether compounds **1**–**4** could prevent cell damage induced by cisplatin, a platinum-based anticancer drug, in LLC-PK1 cells. Co-treatment with **2** and **3** ameliorated the damage of LLC-PK1 cells induced by 25 μM of cisplatin. In particular, the effect of compound **2** at 100 μM (cell viability, 90.68 ± 0.81%) was similar to the recovered cell viability of 88.23 ± 0.25% with 500 μM *N*-acetylcysteine (NAC), a positive control.

## 1. Introduction

Marine invertebrates host a diverse assemblage of mostly beneficial microbes that play important roles in host development, fitness, and protection [1,2]. To ensure propagation within these often extreme and highly competitive host-specific microenvironments, microorganisms have developed unique metabolic and physiological capabilities that aid in their survival [3,4]. Amongst many other important biochemical traits, marine microorganisms are well known to produce structurally diverse and unique secondary metabolites, which often show important pharmacological activities, such as antioxidant, antibiotic, anticancer, or anti-inflammatory activities [5,6,7,8,9]. Previous reports have shown interesting marine natural products, e.g., hypochromins A and B were isolated from *Hypocrea vinisa*; penicillinolide A, a 10-membered lactone, was purified from *Penicillium* sp., and *p*-hydroxyphenopyrrozin was identified from *Chromocleista sphaerospermum* [10,11,12].

As a part of our continuing endeavor to discover novel bioactive natural products from various natural sources [13,14,15,16,17], we recently analyzed the marine fungus *Cladosporium sphaerospermum* sp. SW67, which was obtained from the polyp surface of the marine invertebrate *Hydractinia echinata* (Cnidaria). Subsequent co-culture assays and comparative metabolomics studies led to the isolation and characterization of three novel spirocyclic natural products containing a tetramic acid core—namely, cladosporicin A and cladosporiumins I and J—as well as the isolation of previously reported stereoisomers [18]. Based on our acquired whole genome sequence of *C. sphaerospermum* sp. SW67 and gene expression studies, we proposed a putative PKS-NRPS hybrid gene cluster (*cls*) as a genetic basis for the tetramic acid-derived metabolites [18].

Intriguingly, a previous study exploring the metabolites of the related deep-sea-derived fungus *C. sphaerospermum* sp. 2005-01-E3 led to the characterization of structurally-related hybrid polyketides containing 2,4-pyrrolidinedione (tetramic acid) derivatives, named cladosins A-E [19]. In addition, cladosins H-K, featuring tetramic acid derivatives with aniline moiety, were identified from the deep-sea-derived fungus *C. sphaerospermum* L3P3 [20]. These studies and the intriguing HPLC-UV profiles of our fungal extract prompted us to re-analyze our culture extracts resulting in the isolation of five hybrid polyketides (**1a**, **1b**, and **2**–**4**) containing tetramic acid core including a new fungal hybrid polyketide named cladosin L (**1**). Herein, we describe the isolation and structural characterization of the hybrid polyketides and their plausible biogenetic pathway, as well as their bioactivity screening.

## 2. Results and Discussions

### 2.1. Isolation and Structural Characterization

**Figure 1:**
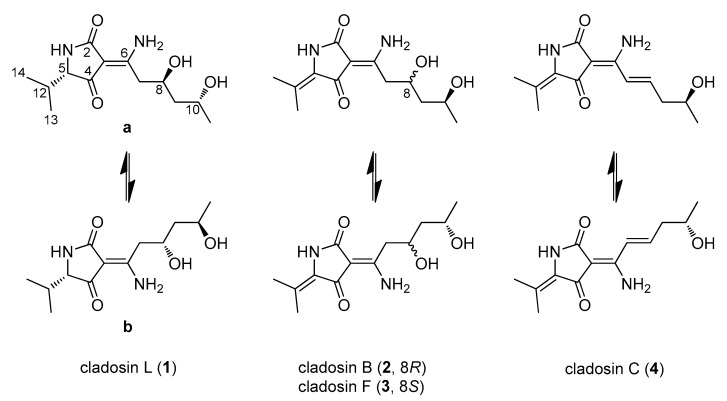
Chemical structures of compounds **1**–**4** from *C. sphaerospermum* SW67.

*C. sphaerospermum* SW67 was cultivated on a large scale on PDA and MEA agar plates for 14 days at 25 °C. Mycelium-covered plates were then extracted with MeOH and then filtered and evaporated to afford the crude culture extract. Subsequent solvent-partitioning of the extract and LC/MS-guided chemical analysis of the fractions combined with semi-preparative C18 reverse-phase HPLC yielded five hybrid polyketides (**1a**, **1b**, and **2**–**4**) with a very similar NMR signal pattern (Figure 1).

**Figure 2:**
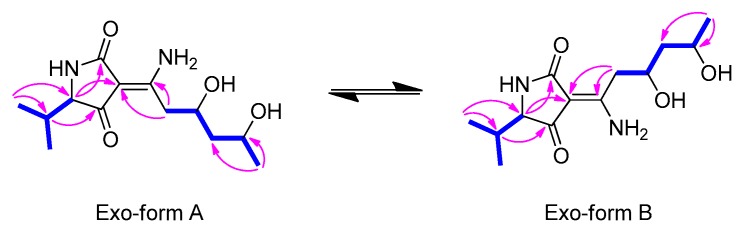
Key COSY (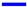) and HMBC (→) correlations for **1**.

Compound **1** was isolated as a yellowish oil with a negative optical rotation, [α]${}_{D}^{25}$−25.5 (*c* 0.05, MeOH) and as an inseparable mixture of two geometric isomers (**1a**:**1b**) present in an approximate ratio of 1:1. The mixture (**1a**/**1b**) was determined to have the same molecular formula of C_13_H_22_N_2_O_4_, requiring five degrees of unsaturation by the adduct ion of *m/z* 271.1658 [M+H]^+^ (calcd. for C_13_H_23_N_2_O_4_, 271.1658) detected in HR-ESI-MS. The IR spectrum showed absorption bands for the hydroxyl groups at 3435 cm^−1^ and carbonyl groups at 1634 cm^−1^. The ^1^H and ^13^C NMR data (Table 1) exhibited three methyls, two methylenes, and four methines including two oxygenated methines, an amide-like carbonyl, an *α*/*β*-unsaturated ketone, and two non-protonated sp^2^ carbons. Its NMR spectral features were very similar to those of the previously described cladosins B (**2**) and F (**3**), which were also isolated in the present work, except for the apparent difference in the chemical shifts of the methyl groups and one additional methine signal set at *δ*_H_ 3.59/3.66 and *δ*_C_ 68.0/67.0. The gross structure of **1a** was assembled by extensive 2D NMR analysis (Figure 2). The COSY correlations from H_2_-7 (*δ*_H_ 2.85 and 3.17)/H-8 (*δ*_H_ 4.14)/H_2_-9 (*δ*_H_ 1.59 and 1.60)/H-10 (*δ*_H_ 3.99)/H_3_-11 (*δ*_H_ 1.18) unambiguously established the spin system from C-7 to C-11 with the oxygen attached to C-8 and C-10. This partial structure was connected to the Δ^3(6)^ double bond as evidenced by the key HMBC correlations from H-7 (*δ*_H_ 2.85 and 3.17) to C-3 (*δ*_C_ 99.1) and C-6 (*δ*_C_ 172.3). The HMBC correlations from H_3_-13 (*δ*_H_ 0.77) and H_3_-14 (*δ*_H_ 1.01) to C-5 (*δ*_C_ 68.0) and C-12 (*δ*_C_ 32.1) led to the identification of an isobutyl moiety in the molecule. That the isobutyl unit was linked to the 2,4-pyrrolidinedione (tetramic acid) scaffold was based on the HMBC correlations from H-5 (*δ*_H_ 3.59) to the amide carbonyl (C-2, *δ*_C_ 178.3), olefinic carbon (C-3, *δ*_C_ 99.1), and *α*/*β*-unsaturated ketone carbon (C-4, *δ*_C_ 199.8). Finally, the amino group was placed on C-6 as deduced by the molecular formula of C_13_H_22_N_2_O_4_. Comparison of the NMR data of **1a** and **1b** suggested that they had a similar planar structure. The only significant differences between **1a** and **1b** were the chemical shifts of carbons attributable to the 2,4-pyrrolidinedione scaffold (Table 1), indicating that **1** existed as a pair of interconverting geometric isomers in solution [19]. The probability of the geometric isomers of **1** could be explained by the interconversion of keto-enamine and enol-imine forms [21,22]. However, the isomers **1a**/**1b** existed only in the keto-enamine form (Figure 1), which was explained through a conversion via tautomerism of the 3-acyltetramic acids. The geometric forms of **1a** and **1b** were determined as exo-form A (Δ^3(6)^: *Z*) and exo-form B (Δ^3(6)^: *E*) since the chemical shift of a carbonyl group forming the hydrogen-bond with amine group, is more downshifted when compared to that of the corresponding free carbonyl group in ^13^C NMR data [21,22].

**Figure 3:**
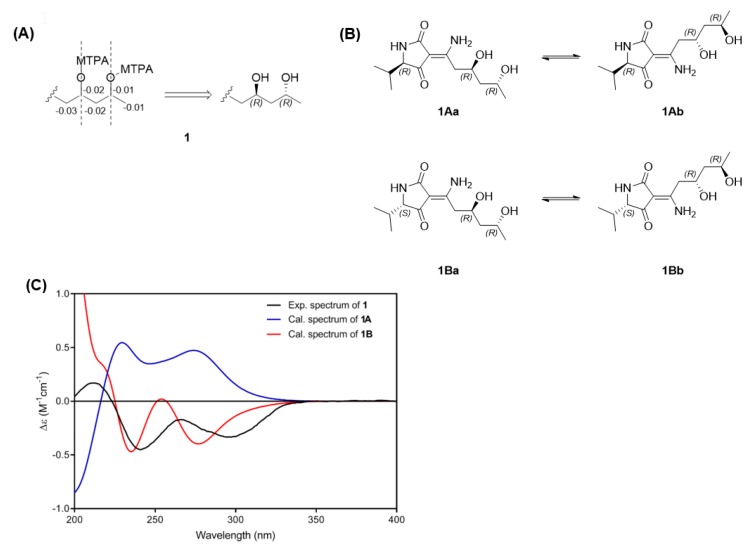
(**A**) Analysis of the modified Mosher’s method for **1**. ∆*δ* (*δ*_S_–*δ*_R_) values are shown. (**B**) Four diastereomers of **1**. (**C**) Experimental ECD spectrum of **1** and calculated ECD spectra of **1A/1B**.

The relative configuration of the stereogenic carbons of the pentan-1,3-diol (C-8 and C-10) of **1** was determined based on the characteristic signals for C-9 methylene protons (methylene group between the 1,3-diol), which were overlapped in *anti*-1,3-diols whereas, they appeared as two sets of multiplets in *syn*-1,3-diols [19,23]. Since the signals of the methylene protons of C-9 in **1** were overlapped at *δ*_H_ 1.58–1.60, the 1,3-diol was determined as *anti*. The absolute configuration of the *anti*-1,3-diol system was determined by the modified Mosher’s method. A detailed analysis of ^1^H NMR and TOCSY spectra of the (*R*)- and (*S*)-di-MTPA ester derivatives of **1** indicated that the distributed ∆*δ* values were negative (∆*δ*_L_), negative (∆*δ*_C_), and negative (∆*δ*_R_), which implied that the absolute configurations of both C-8 and C-10 were (*R*) (Figure 3A) [24]. To establish the absolute configuration of C-5 in **1**, the experimental ECD spectrum of **1** was compared to the calculated ECD spectra of two possible epimers (**1A**, 5(*R*); **1B**, 5(*S*), Figure 3B). Since **1** exists as 1:1 mixture of two geometric isomers, total four ECD spectra of **1Aa**, **1Ab**, **1Ba**, and **1Bb** were simulated (Figure S22) and the ECD spectra of the two geometric isomers (**1Aa** and **1Ab**; **1Ba** and **1Bb**) were averaged to generate two ECD spectra of **1A** and **1B** (Figure 3C). The experimental ECD spectrum of **1** showed two negative Cotton effects at 241 and 297 nm, which corresponded to two negative Cotton effects at 235 and 277 nm of **1B** spectrum whereas **1A** spectrum exhibited two positive Cotton effects at 230 and 274 nm. Therefore, the C-5 configuration was assigned as (*S*), which is in agreement with previously reported structures of cladosporiumins G and H; both were reported to be built up by L-valine. Accordingly, the complete structure of **1,** named cladosin L, is shown in Figure 1.

The known hybrid polyketides, isolated from *C. sphaerospermum* SW67, were identified as cladosin B (**2**), cladosin F (**3**), and cladosin C (**4**) by comparison of their NMR data with those reported in the literature [19,25].

### 2.2. Proposed Biogenesis of ***1**–**4***

**Figure 4:**
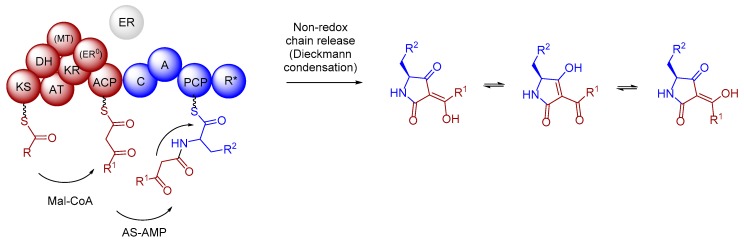
Fungal iterative hybrid PKS–NRPS megaenzymes are responsible for the formation of tetramic acid derivatives and often require a discrete ER to be completely functional. The R-domain catalyzes a non-reductive Dickmann condensation to release the product from the enzyme. Tetramic acids exists often as mixture of rapidly interconverting tautomers in solution arising from C–C bond rotation of the acyl side chain.

It is generally assumed that tetramic acid-containing compounds, such as cladosins and structurally related natural products [26], are built up by polyketide synthases (PKS)–non ribosomal peptide synthetases (NRPS) hybrid megaenzymes, which catalyze the formation and condensation of a tetraketide or a related unsaturated or reduced polyketide unit and an activated amino acid to yield the 1,3-dione-5,7-diol conjugate (Figure 4) [27,28,29].

In fungi, these PKS/NRPS hybrid gene clusters generally consist of a single iterative PKS module (incorporating acetate building blocks), which is followed by a single NRPS module (attaching the polyketide chain to an amino acid) and an offloading domain. The domain architecture of the iterative PKS module closely resembles the mammalian fatty acid synthases (FAS) with the following domain organization: KS (ketosynthase), AT (acyltransferase), DH (dehydratase), CMeT (C-methyltransferase), *KR (a structural domain variant of a ketoreductase), ER (enoylreductase), KR (ketoreductase), and ACP (acyl carrier protein). In contrast to FAS, modules of an iterative PKS within hybrid organizations are more promiscuous and produce individual and diverse polyketide chains with different reduction and methylation patterns [27,28,29]. In particular, enoyl reduction carried out by the ER domain in polyketide biosynthesis adds diversity to the growing PKS chain. However, sequence alignments of fungal ER domains in iterative PKS–NRPSs showed that almost all ERs are predicted to be inactive (ER^0^) due to a variety of significant sequence differences, e.g., missing a typical GGVG motif for NADPH binding. In most cases, *trans*-acting ERs were found to be necessary for the respective product formation [27,28,29]. The NRPS modules of iterative NRPS/PKS hybrids have the general domain organization of C (condensation), A (adenylation) and T (thiolation, also known as PCP peptide carrier protein). Different release mechanisms are responsible for offloading from PKS, NRPS or their respective hybrids, which are catalyzed by the respective enzymatic domains such as R (reductase), DKC (Dieckmann cyclase) or TE (thioesterase) [30].

A previous genome mining approach of the cladosin producing organism *Cladosporium* sp. SW67 and the related strain *Cladosporium* sp. UM843 revealed a putative PKS-NRPS cluster (*clsA-clsL*), which is proposed to be responsible for the formation of cladosins, cladosporiumins and cladosporicins. The region of the gene cluster *cls* encodes for five genes: a siderophore esterase (*clsA*), an AMP dependet synthetase/ligase (*clsD*), a γ-glutamyl transferase (*clsF*), a cytochrome P450 (*clsK*) and a PKS-NRPS hybrid gene (*clsI*), of which, only the cytochrome P450 (*clsK*) and the PKS-NRPS hybrid (*clsI*) gene appear to be necessary for tetramic acid formation. The possible roles of *clsA*-*F* remain unclear.

The domains of the putative PKS-NRPS were predicted as KS-AT-X-Y-KR-C-A-PCP-TD using PKS/NRPS analysis predictor [31], Blast searches [32] and Antismash [33]. Here, two domains (X, Y) located between the AT and the KS domain of the PKS-NRPS hybrid enzyme ClsI could not be assigned despite intensive manual database analyses of the three most similar genes using e.g., MIBig [34] and UniProt [35]. Here, it needs to be acknowledged that until today only limited numbers of fungal PKS/NRPS domains have been fully characterized, which clearly limits the assignments based on homology searches.

To identify possible conserved motifs within the unknown domains, the corresponding areas were extracted by using ClustalW implemented in Bioedit 7.2.0 [36] and aligned against each other (Table 2). Based on different motif alignments, we found that the first unidentified domain (X) within *clsI* shows weak homologies to known dehydratase (DH) domains. In general, dehydratase (DH) domains carry a characteristic catalytic proline and aspartic acid residues within a characteristic conserved H**P**ALL**D** motif [37]. Alignment of the unknown domain within ClsI and a related domain in CluI detected in *Cladosporium* sp. UM843 revealed a T**P**MAA**D** motif, which is in weak accordance with so far reported homologous (Table S8).

Subsequently, we analyzed the second unknown domain by different motif alignments. Genes with the highest identities for already described PKS-NRPSs mainly contained methyltransferase (C-MT) domains at the corresponding sites. Regions of sequence similarities within SAM dependent MTs are divided into three different motifs (I, II and II) [38,39] and comparative alignments between the unknown second domains (Y) and known C-MT domains showed that neither Motif I [(L/I/V)(V/L)(E/D)(V/I)G(C/G)G(P/T)G] nor Motif III [(G/P)(T/Q)(A/Y/F)DA(Y/V/I)(I/F)(L/V/C)] was detectable within the second domain (Y). However, weak homologies to Motif II [LL(K/R)PGG(L/I/R) (I/L)(V/I/F/L)(L/I)] could be detected in both unknown domains (Y) of *Cladosporium* sp. SW67 and UM843 (Table 3).

**Figure 5:**
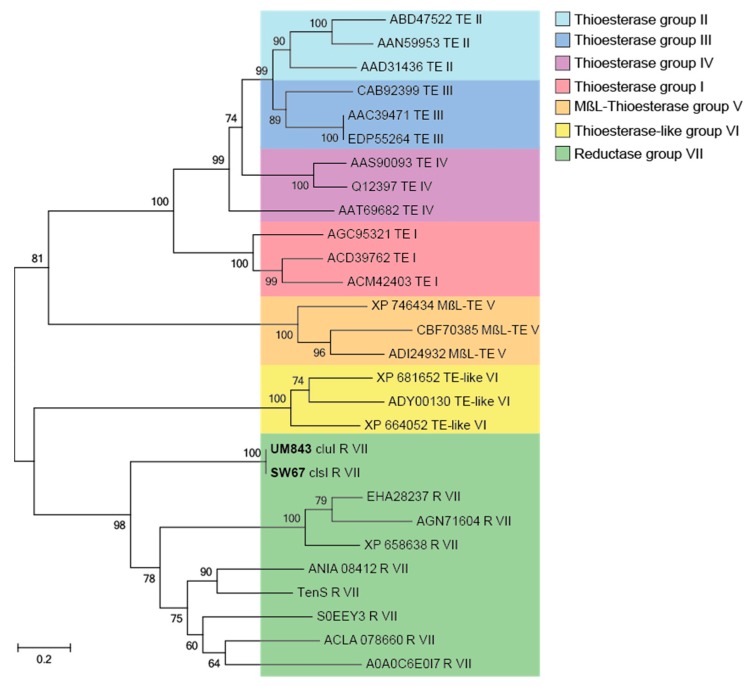
Phylogenetic tree of different fungal product-releasing and their functional classification. The different types of product-releasing enzyme domains in NR-PKSs have been highlighted with different color code [42]. Phylogenetic analysis was conducted using neighbor joining method [43,44]. Bold text represents the *Cladosporium* sp. SW67 and UM843 group (accession number/gene name, type of release reaction, group of product-release enzyme). Probability values > 50% are shown at the nodes based on 1000 bootstraps. The tree is drawn to scale with branch lengths measured in the number of substitutions per site.

In a last step, we analyzed the catalytic domain responsible for product-release from the PKS-NRPS enzyme. The formation of the 1,3-dione-5,7-diol conjugate requires a Dieckmann-type condensation/cyclization of a polyketide unit and a valine residue (Figure 5). To analyze if the proposed PKS-NRPS enzymes harbors the necessary release domain [30,40]. Thus, amino acid sequences of NR-PKSs enzymes with known product release mechanisms were again collected from NCBI and UniProt databases [35] and closest homologues were obtained by identity search in the MiBIG database [34] and the first three matches were individually evaluated by BLAST. The product-release enzyme domains of *clsI* and *cluI* were extracted by PKS/NRPS Analyzer and manually aligned using the ClustalW tool implemented in Bioedit 7.2.0 and partial sequences were manually compiled, assembled and trimmed (Tables S8 and S9). Initial tree(s) for the heuristic search were obtained automatically by applying neighbor joining approach. The robustness of branches was assessed by bootstrap analysis with 1000 replicates. The tree is drawn to scale with branch lengths measured in the number of substitutions per site and evolutionary analyses were conducted in MEGA7 [41].

**Figure 6:**
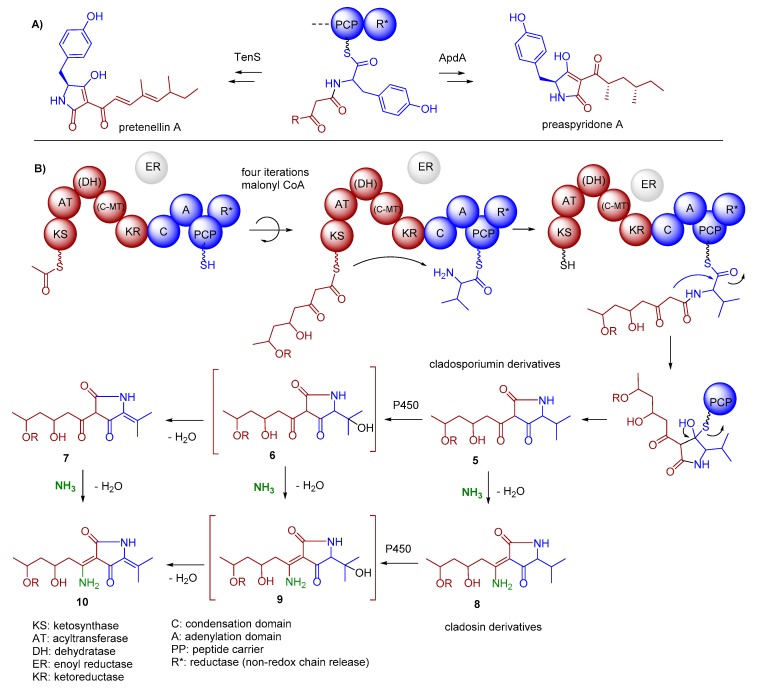
(**A**) TenS and ApDA as examples of fungal iterative hybrid PKS–NRPS that include a non-reductive Dieckmann-type condensation reactions yielding peraspyridone A and pretenellin A, respectively. (**B**) Proposed biosynthetic pathway of the core structure formation of cladosporiumin and cladosin-type natural products. Domains of PKS-NRPS are putatively assigned as KS-AT-(*DH)-(*C-MT)-KR-C-A-PCP-TE (* weak homologies).

Due to similarities and cluster grouping, the product release domain of *Cladosporium* SW67 and UM843 can be classified as reductase (R) domain (Figure 4). This domain was initially found to catalyze an NAD(P)H-dependent reductive release of fungal iterative PKS–NRPSs products based on sequence homology to SDR (shortchain dehydrogenase/reductase) family proteins. Detailed studies on tenellin [45,46] and aspyridone A [47] biosynthesis showed that R domains also catalyze non-reductive Dieckmann-type condensation reactions yielding the tetramic acids derivatives peraspyridone A and pretenellin A (Figure 6A) [48]. In case of peraspyridone A, subsequent oxidation by an additional P450 monooxygenase yields aspyridone A; a similar mechanism was proposed for the biosynthesis of tenellin A.

In summary, we propose the following domain arrangement of the putative PKS-NRPS (KS-AT-*DH-*cMT-KR-C-A-PCP-R) (Figure 6B). Whether or not the poorly assigned domains (-*DH-*cMT-) are fully functional, cannot be deduced at this stage. Here, we also propose that the identified terminal reductase (R) domain in *clsI* and *cluI* performs a non-reductive Dieckmann-type cyclisation to yield the 2,4-pyrrolidinedione skeleton, which after oxidation of the valine unit (cytochrome P450 *clsK*) and (spontaneous) elimination of H_2_O results in derivative **7** bearing a thermodynamically stable tetra-substituted double bond (Figure 6B). Whether or not the cytochrome P450 *clsK* [49] is also responsible for the elimination of H_2_O cannot be deduced at this stage.

**Figure 7:**
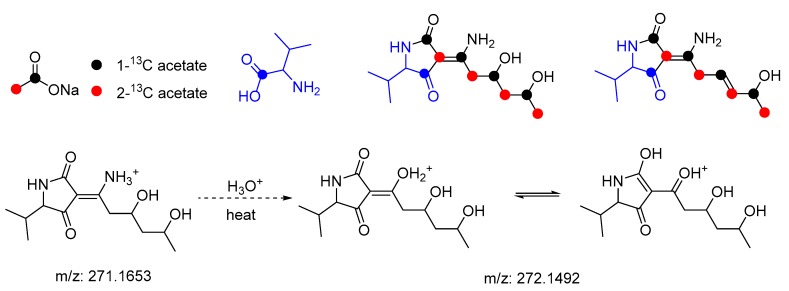
The proposed ^13^C-labeling pattern of **1**–**4** based on a hybrid PKS-NRPS assembly line.

Tetramic acids of types **5** and **7** exist in both, keto and enol form, which allows for the (spontaneous) reaction with NH_3_ yielding enamines **8** and **10**, respectively. Due to a tautomeric equilibrium, enols and imines were found as interconverting *E/Z*-mixtures. To confirm the biosynthetic origin, we re-analyzed our ^13^C-labeling data (addition of ^13^C-valine and ^13^C-acetate to a growth medium, Figure 7). As expected, an LC/MS based analysis of the enriched ^13^C-labeled compound mixture revealed the incorporation of one valine residue and a PKS-typical CH_3_CO_2_-dependent pattern (incorporation of four acetate units) into **1** (Figure S15) confirming once more the biosynthetic proposal.

### 2.3. Biological Evaluation of ***1**–**4***

In our ongoing studies to explore biologically active unique natural products, we particularly focused on the discovery of renoprotective natural products for protection against cisplatin-induced nephrotoxicity [50,51,52], which is a major issue in the clinical use of cisplatin in cancer patients [53,54,55]. It has been reported in the literatures that some natural products exhibit effective protection against cisplatin-induced nephrotoxicity [55,56,57]. In our recent studies, it was found that 1-*O*-(2-aminobenzoyl)-α-L-rhamnopyranoside from a termite-associated *Streptomyces* sp. RB1 [50], dehydroeburicoic acid monoacetate from *Poria cocos* [51], and sterols, (22*E*,24*S*)-ergosta-7,22-diene-3β,5α,6β-triol and (22*E*,24*R*)-ergosta-8(14),22-diene-3β,5α,6β,7α-tetrol from *Pleurotus cornucopiae* [52] showed the renoprotective effects. Despite the continued publications of these effects, these studies failed to achieve clinical success. Therefore, there is an urgent need to develop effective agents to save the lives of cancer patients having impaired renal functions.

**Figure 8:**
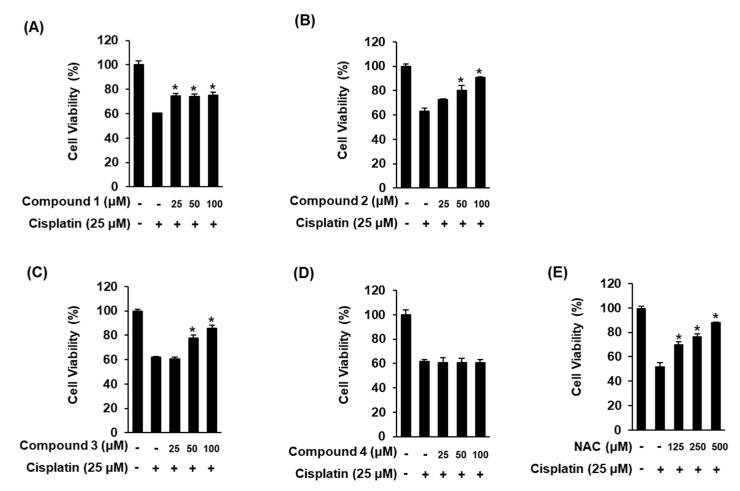
Comparison of the protective effects of **1**–**4** (**A**–**D**) and NAC (**E**, positive control) on the viability of LLC-PK1 cells exposed to 25 μL of cisplatin for 24 h by MTT assay. The control cells were treated with the vehicle only (mean ± SD, * *p* < 0.05 vs. control).

To identify active compounds having protective effects against cell damage caused by cisplatin, the LLC-PK1 cells were pre-treated with **1**–**4** for 2 h before treatment with 25 μM of cisplatin. After treatment with cisplatin, the cell viability was 63.01 ± 2.47% when compared to the control. The reduction in cell viability in response to cisplatin-induced cell death was recovered to 80.14 ± 4.16% and 90.68 ± 0.81% after pretreatment with 50 μM and 100 μM of **2**, respectively (Figure 8B). Cell viability was reduced by 61.93 ± 0.72% after treatment with 25 μM of cisplatin, whereas it was increased by 77.65 ± 2.43% and 85.6 ± 2.47% after treatment with 50 μM and 100 μM of **3**, respectively (Figure 8C). Although the protective effects of **2** and **3** were similar, **2** showed a better effect. In addition, the effect of **2** at 100 μM was similar to the recovered cell viability of 88.23 ± 0.25% with 500 μM *N*-acetylcysteine (NAC), a positive control (Figure 8E). Compound **4** showed no protective effect and **1** showed minor but insignificant protective effect (Figure 8A,D). In the structure activity relationship (SAR), the comparison of protective effects of active compounds (**1**, **2** and **3**) vs inactive compounds (**4**) suggested that the presence of hydroxy group at C-8 may be essential for the renoprotective effect against cisplatin-induced nephrotoxicity in LLC-PK1 cells. Therefore, there is a need to further evaluate the mechanisms of action of **2** and **3** against cisplatin-induced damage to LLC-PK1 cells.

## 3. Materials and Methods

### 3.1. General Experimental Procedures

Optical rotations were measured using a Jasco P-1020 polarimeter (Jasco, Easton, MD, USA). The IR spectra were recorded on a Bruker IFS-66/S FT-IR spectrometer. The ESI and HR-ESI mass spectra were recorded on an SI-2/LCQ DecaXP Liquid chromatography (LC)-mass spectrometer. The experimental ECD spectra in MeOH were acquired in a quartz cuvette of 1 mm optical path length on a JASCO J-1500 spectropolarimeter (Tokyo, Japan). NMR, including COSY, HSQC, and HMBC, experiments were conducted utilizing a Varian UNITY INOVA 800 NMR spectrometer operating at 800 MHz (^1^H) and 200 MHz (^13^C) with chemical shifts given in ppm (δ). The preparative high-performance liquid chromatography (HPLC) utilized a Waters 1525 Binary HPLC pump with Waters 996 Photodiode Array Detector (Waters Corporation, Milford, CT, USA). Semi-preparative HPLC was carried out using a Shimadzu Prominence HPLC System with SPD-20A/20AV Series Prominence HPLC UV-Vis Detectors (Shimadzu, Tokyo, Japan). LC/MS analysis was carried out on an Agilent 1200 Series HPLC system (Agilent Technologies, Santa Clara, CA, USA) equipped with a diode array detector and a 6130 Series ESI mass spectrometer using an analytical Kinetex (4.6 × 100 mm, 3.5 μm). Merck precoated Silica gel F254 plates and RP-18 F254s plates were used for thin-layer chromatography (TLC). The spots were detected on TLC under UV light or by heating after spraying with anisaldehyde-sulfuric acid.

### 3.2. Biological Material

The fungus was obtained as previously described [18]. In brief, polyps of *H. echinata* (Alfred Wegener Institute, Sylt, Germany) were aseptically homogenated, diluted with sterile filtered seawater, and plated onto potato dextrose agar (PDA) plates. The plates were incubated for 1–3 weeks at room temperature, and colonies showing fungal morphologies were selected. The isolate SW67 was identified as *C*. *sphaerospermum* based on the analysis of the internal transcribed spacer (ITS) gene sequence. 

### 3.3. Extraction and Isolation

Overall, approximately 400 PDA and 400 MEA plates were inoculated with a 100 µL aliquot of a turbid fungal spore suspension of C. *sphaerospermum* SW67 in sterile PBS. The suspension was evenly distributed over the agar surface, and the plates were incubated at 25 °C in the dark for 14 days. The agar was then cut into squares, consolidated, and macerated overnight in MeOH. The MeOH solution was filtered and removed under reduced pressure to obtain the crude MeOH extract. The MeOH extract (6 g) was dissolved in distilled water (700 mL) and then partitioned with EtOAc (700 mL), yielding 0.25 g of EtOAc-soluble fraction. The EtOAc-soluble fraction was fractionated by preparative reversed-phase HPLC (Phenomenex Luna C18, 250 × 21.2 mm i.d., 5 μm) using CH_3_CN-H_2_O (1:9–3:2, v/v, gradient system, flow rate: 5 mL/min) to afford six subfractions (A–F). Compound **1** (2.0 mg, *t*_R_ = 29.0 min) was purified from subfraction B (15 mg) by semi-preparative reversed-phase HPLC (Phenomenex Luna C18, 250 × 10.0 mm i.d., 5 μm) with an isocratic solvent system of 29% MeOH. The subfraction C (14 mg) was purified by semi-preparative reversed-phase HPLC (Phenomenex Luna C18, 250 × 10.0 mm i.d., 5 μm) with an isocratic solvent system of 31% MeOH to afford **2** (3.0 mg, *t*_R_ = 36.0 min) and **3** (7.4 mg, *t*_R_ = 40.0 min). Compound **4** (1.7 mg, *t*_R_ = 39.0 min) was isolated from subfraction D (8 mg) by semi-preparative reversed-phase HPLC (Phenomenex Luna C18, 250 × 10.0 mm i.d., 5 μm) with an isocratic solvent system of 38% MeOH.

#### Cladosin L (**1**)

Yellowish oil; [α]${}_{D}^{25}$ −25.5 (*c* 0.05, MeOH); IR (KBr) ν_max_ 3435, 2966, 2843, 1634, 1521, and 1060 cm^−1^; UV (MeOH) λ_max_ (log ε) 198 (2.5), 236 (1.9), and 296 (3.9) nm; ECD (MeOH) λ_max_ (△ε) 211 (1.7), 241 (−3.2), 266 (−1.6), and 297 (−2.5) nm; ^1^H (800 MHz) and ^13^C NMR (200 MHz), see Table 1; (+)-HRESIMS *m/z* 271.1658 [M + H]^+^ (calcd. for C_13_H_23_N_2_O_4_, 271.1658).

### 3.4. Preparation of Mosher Ester Derivatives from Cladosin L *(**1**)*

Cladosin L (**1**) (0.5 mg) was dissolved in deuterated pyridine (0.25 mL) and transferred to a clean NMR tube and then a small quantity of 4-(dimethylamino)pyridine was added. (*S*)-(+)-*α*-methoxy-*α*-(trifluoromethyl) phenylacetyl (MTPA) chloride (5 µL) was added into the NMR tubes under an N_2_ gas stream and the NMR tube was shaken carefully to mix the sample with added reagents. The NMR tube was stored at room temperature overnight, which afforded the (*R*)-MTPA ester derivative of **1**. The (*S*)-MTPA ester derivative of **1** was also prepared using (*R*)-MTPA chloride according to the procedure described above. The ^1^H NMR and TOCSY spectra were directly obtained from the Mosher’s ester derivatives of **1** in NMR tube.

*(R)-MTPA Ester of***1**. ^1^H NMR (Pyridine-*d*_5_, 800 MHz): *δ* 3.44 (H-7), 4.99 (H-8), 2.04 (H-9), 4.59 (H-10), 1.37 (H-11); ESIMS m/z 725.2 [M + Na]^+^.

*(S)-MTPA Ester of***1**. ^1^H NMR (Pyridine-*d*_5_, 800 MHz): *δ* 3.41 (H-7), 4.97 (H-8), 2.02 (H-9), 4.58 (H-10), 1.36 (H-11); ESIMS m/z 725.2 [M + Na]^+^.

### 3.5. Computational Analysis

All conformers of **1Aa**, **1Ab**, **1Ba**, and **1Bb** used in this study were found using the Macromodel (version 2015-2, Schrödinger LLC, New York, NY, USA) module with “Mixed torsional/Low-mode sampling” in the MMFF force field. The searches were implemented in the gas phase with a 5 kJ/mol energy window limit and 10,000 maximum number of steps to explore all potential conformers. The Polak–Ribiere Conjugate Gradient (PRCG) method was utilized to minimize conformers with 10,000 iterations and a 0.001 kJ (mol Å)^−1^ convergence threshold on the Root Mean Square (RMS) gradient. All the conformers were subjected to geometry optimization using the Gaussian 16 package (Gaussian Inc., Wallingford, CT, USA) in the gas phase at B3LYP/6-31+G(d,p) level and proceeded to calculation of excitation energies, oscillator strength, and rotatory strength at B3LYP/6-31+G(d,p) level in the Polarizable Continuum Model (PCM, methanol). The ECD spectra were Boltzmann-averaged based on the calculated Gibbs free energy of each conformer (Tables S2–S5) and visualized with SpecDis software (Version 1.71) [58] with a σ/γ value of 0.30 eV. The calculated ECD spectra of **1A** and **1B** were obtained by averaging those of **1Aa** and **1Ab,** and **1Ba** and **1Bb**, respectively, since the compound **1** exists as a 1:1 mixture of two geometric isomers (**1a** and **1b**) as described in the Section 2.1 above.

### 3.6. Isotope Labeling

For ^13^C labeling experiments, 2 g/L of sodium [1-^13^C] acetate, sodium [2-^13^C] acetate, or [1-^13^C] valine was each added separately to 1 L of PDA medium (6.6 g/L). Each plate was inoculated with 100 µL of a spore suspension (SW67) in sterile PBS, and the plates were incubated for 14 d at room temperature in the dark. The plates were cut into pieces and extracted with 100% MeOH (if not mentioned otherwise, mixtures refer to MeOH in ddH_2_O) at 4 °C overnight. The extracts were filtrated and the solvent was evaporated under reduced pressure. The remaining extract was re-dissolved in 10% MeOH and loaded on a pre-activated and equilibrated C18 cartridge (100 mg C18, 10% MeOH). The loaded SPE column was washed with 20% MeOH, and then the metabolites were eluted using 100% MeOH. The extracts were then concentrated under reduced pressure. Finally, the organic extract was dissolved with 100% MeOH to yield a 1.0 mg/mL stock solution for the UHPLC-MS analysis.

For compound **1** (*m/z* = 271.15 [M − H]^−^), feeding with [1-^13^C] sodium acetate and [2-^13^C] sodium acetate, respectively, resulted in the detection of the corresponding mass shift of up to +4 *m/z.* Similar, addition of [1-^13^C] valine resulted in the detection of the corresponding mass shift of +1 *m/z*. However, it needs to be noted that a dominant signal (*m/z* 270) of the closely related cladosporiumin F overlapped with the respective signal of compound **1** due to their similar retention times. If the high detection level of cladosporiumin F was due to heat/acid-induced hydrolysis during measurement, it could not be excluded at this stage. Both the signal sets showed the same ^13^C incorporation pattern.

### 3.7. Renoprotective Effects against Cisplatin-Induced Kidney Cell Damage

The protective effects of compounds **1**–**4** against cisplatin-induced renal cell death were determined by Ez-Cytox cell viability assay kit (DOGEN, Seoul, Korea) using LLC-PK1 pig kidney epithelium cells (American Type Culture Collection, Rockville, MD, USA). The LLC-PK1 cells were cultured in Dulbecco’s modified Eagle medium (Manassas, VA, USA) supplemented with 10% FBS, 1% penicillin/streptomycin, and 4 mM L-glutamine at 37 °C with 5% CO_2_ in a humidified incubator and with 5% CO2 in air at 37 °C. The cells were seeded onto 96-well culture plates at 1 × 10^4^ cells per/well and incubated for 24 h to adhere. Thereafter, the cells were pre-treated with 25, 50, and 100 μM of compounds **1**–**4**, and then 25 μM of cisplatin was added to the wells. After 24 h, the medium containing the compounds **1**–**4** and/or cisplatin was removed, and a serum-free medium containing Ez-Cytox reagent was added to the wells. After incubation for 2 h at 37 °C, the cell viability was measured by absorbance at 450 nm using a microplate reader (PowerWave XS; Bio-Tek Instruments, Winooski, VT, USA). *N*-acetyl cysteine (NAC) was used as a positive control.

### 3.8. Statistical Analyses

All data described in this study were repeated at least three times and are represented as the mean ± S.E.M. The statistical significance was determined by one-way analysis of variance (ANOVA). *p*-values of < 0.05 were considered statistically significant.

## 4. Conclusions

Marine invertebrates host a diverse assemblage of mostly beneficial microbes that can produce novel natural products. Herein, we isolated the marine fungus *C. sphaerospermum* SW67 from the hydroid polyp of *H. echinata.* LC/MS-guided chemical analysis of the marine fungus *C. sphaerospermum* SW67 led to the isolation and characterization of five hybrid polyketides (**1a**, **1b**, and **2**–**4**) containing tetramic acid core including a previously unreported cladosin L (**1**), which was isolated as a pair of interconverting geometric isomers. As indicated by biosynthetic gene cluster analysis and ^13^C-labeling studies, compounds **1**–**4** are formed via the generally assumed biosynthesis of microbial tetramic acids. Although the timing of the putative oxygenation and dehydration of the valine residue is unknown, the compound **1** could be regarded as a putative precursor of previously and herein reported cladosin F (**3**). Compounds **2** and **3** ameliorated the damage of LLC-PK1 cells induced by 25 μM of cisplatin. In particular, **2** at 100 μM (cell viability, 90.68 ± 0.81%) exhibited a significant protective effect against cell damage, which was similar to that of the positive control, 500 μM NAC (cell viability, 88.23 ± 0.25%). These findings provide experimental evidence that **2** could be an adjunct candidate to treat cisplatin-induced adverse effects and/or to prevent anticancer drug-induced nephrotoxicity.

## Figures and Tables

**Table 1:** ^1^H (800 MHz) and ^13^C (200 MHz) NMR data of **1** in CD_3_OD.

Position	1			
	Exo-Form A (a)		Exo-Form B (b)	
	*δ*_H_ (*J* in Hz)	*δ*_C_, typ	*δ*_H_ (*J* in Hz)	*δ*_C_, typ
2		178.3, CO		176.5, CO
3		99.1, C		97.7, C
4		199.8, CO		202.0, CO
5	3.59, d (3.0)	68.0, CH	3.66 d (3.0)	67.0, CH
6		172.3, C		172.7, C
7	2.85, m; 3.17, dd (13.0, 4.0)	41.1, CH_2_	2.82 m; 3.13 dd (13.5, 4.5)	42.1, CH_2_
8	4.14, m	69.6, CH	4.13 m	69.2, CH
9	1.59, m; 1.60, m	48.0, CH_2_	1.59 m; 1.60 m	47.8, CH_2_
10	3.99, m	66.1, CH	3.99 m	66.1, CH
11	1.18, d (6.0)	25.0, CH_3_	1.18 d (6.0)	25.1, CH_3_
12	2.13, m	32.1, CH	2.12 m	32.1, CH
13	0.77, d (7.0)	16.4, CH_3_	0.78 d (7.0)	16.7, CH_3_
14	1.01, d (7.0)	20.7, CH_3_	1.01 d (7.0)	20.6, CH_3_

**Table 2:** Sequence alignment of the first unknown domain of *Cladosporium* SW67 and *Cladosporium* sp. UM843 with DH-domains of known NRPS-PKSs with high identities (Tables S8 and S9). Red marked lines indicate conserved DH motif. Accession numbers are derived from the UniProt database.

	Amino Acid Sequence																													
**SW67_clsI_unknown_1**	**R**	**E**	**W**	**E**	**T**	**Q**	**F**	**Q**	**L**	**T**	**P**	**M**	**A**	**A**	**D**	**S**	**R**	**Y**	**N**	**F**	**R**	**L**	**M**	**I**	**C**	**G**	**P**	**S**	**E**	**S**
**UM843_cluI_unknown_1**	**R**	**E**	**W**	**E**	**T**	**Q**	**F**	**Q**	**L**	**T**	**P**	**M**	**A**	**A**	**D**	**S**	**R**	**Y**	**N**	**F**	**R**	**L**	**M**	**I**	**C**	**G**	**P**	**S**	**E**	**S**
**ACLA_078660_DH**	**F**	**N**	**H**	**S**	**Q**	**P**	**L**	**L**	**I**	**H**	**P**	**A**	**T**	**L**	**D**	**A**	**A**	**I**	**Q**	**S**	**I**	**M**	**L**	**A**	**Y**	**C**	**Y**	**P**	**G**	**D**
**A0A0C6E017_DH**	**D**	**M**	**Q**	**I**	**D**	**N**	**Y**	**V**	**V**	**N**	**P**	**G**	**F**	**L**	**D**	**V**	**A**	**F**	**Q**	**S**	**V**	**Y**	**T**	**A**	**F**	**S**	**S**	**P**	**A**	**S**
**ANIA_08412_DH**	**V**	**P**	**D**	**A**	**D**	**E**	**L**	**L**	**V**	**H**	**P**	**I**	**D**	**L**	**D**	**A**	**A**	**F**	**Q**	**S**	**V**	**M**	**L**	**A**	**Y**	**S**	**Y**	**P**	**G**	**D**
**A0A0C6E0I7_DH**	**P**	**V**	**S**	**W**	**T**	**H**	**T**	**L**	**T**	**H**	**P**	**A**	**P**	**I**	**D**	**T**	**A**	**V**	**Q**	**G**	**L**	**L**	**T**	**A**	**F**	**S**	**F**	**P**	**G**	**D**
**FFUJ_12239_DH**	**F**	**N**	**H**	**S**	**Q**	**P**	**L**	**L**	**I**	**H**	**P**	**A**	**T**	**L**	**D**	**A**	**A**	**I**	**Q**	**S**	**I**	**M**	**L**	**A**	**Y**	**C**	**Y**	**P**	**G**	**D**
**ACLA_078660_DH**	**C**	**L**	**S**	**D**	**T**	**G**	**L**	**L**	**V**	**H**	**P**	**A**	**F**	**L**	**D**	**M**	**T**	**L**	**H**	**A**	**T**	**L**	**A**	**A**	**F**	**A**	**S**	**P**	**G**	**D**
**B1GVX7_DH**	**P**	**V**	**S**	**W**	**T**	**H**	**T**	**L**	**T**	**H**	**P**	**A**	**P**	**I**	**D**	**T**	**A**	**V**	**Q**	**G**	**L**	**L**	**T**	**A**	**F**	**S**	**F**	**P**	**G**	**D**
**A0JJU1_DH**	**A**	**D**	**L**	**N**	**D**	**C**	**Y**	**L**	**V**	**H**	**P**	**A**	**I**	**L**	**D**	**V**	**A**	**F**	**Q**	**T**	**I**	**F**	**V**	**A**	**R**	**A**	**H**	**P**	**D**	**S**
**S0EET5_DH**	**V**	**V**	**P**	**D**	**F**	**P**	**A**	**M**	**I**	**H**	**P**	**A**	**L**	**I**	**D**	**G**	**A**	**F**	**Q**	**S**	**I**	**F**	**A**	**A**	**Y**	**C**	**Q**	**P**	**D**	**D**

**Table 3:** Sequence alignment of the second unknown domain of *Cladosporium* sp. SW67 and *Cladosporium* sp. UM843 and C-MT-domains of known NRPS-PKSs with high identities (Tables S8 and S9). Colored lines indicate conserved MT-domain motif II. Accession numbers are derived from the UniProt database.

	Amino Acid Sequence																													
**SW67_clsI_unknown_2**	**-**	**-**	**-**	**-**	**-**	**-**	**-**	**F**	**D**	**Q**	**E**	**N**	**H**	**R**	**V**	**S**	**P**	**G**	**G**	**C**	**I**	**C**	**V**	**L**	**H**	**S**	**R**	**T**	**-**	**-**
**UM843_cluI_unknown_2**	**-**	**-**	**-**	**-**	**-**	**-**	**-**	**F**	**D**	**Q**	**E**	**N**	**H**	**R**	**V**	**S**	**P**	**G**	**G**	**C**	**I**	**C**	**V**	**L**	**H**	**S**	**R**	**T**	**-**	**-**
**ACLA_078660_C-MT**	**T**	**R**	**D**	**L**	**A**	**Q**	**T**	**V**	**R**	**N**	**V**	**R**	**R**	**L**	**L**	**K**	**P**	**G**	**G**	**Y**	**L**	**L**	**L**	**L**	**E**	**I**	**T**	**E**	**N**	**-**
**A0A0C6E017_C-MT**	**C**	**A**	**R**	**L**	**D**	**E**	**A**	**V**	**A**	**N**	**L**	**R**	**K**	**L**	**L**	**K**	**P**	**G**	**G**	**L**	**L**	**V**	**L**	**G**	**E**	**G**	**A**	**S**	**D**	**G**
**ANIA_08412_C-MT**	**T**	**H**	**S**	**L**	**E**	**N**	**T**	**L**	**R**	**Q**	**C**	**R**	**K**	**L**	**L**	**R**	**P**	**G**	**G**	**R**	**L**	**V**	**L**	**L**	**E**	**I**	**T**	**R**	**-**	**-**
**A0A0C6E0I7_C-MT**	**T**	**R**	**D**	**L**	**A**	**Q**	**T**	**V**	**R**	**N**	**V**	**R**	**R**	**L**	**L**	**K**	**P**	**G**	**G**	**Y**	**L**	**L**	**L**	**L**	**E**	**I**	**T**	**E**	**N**	**-**
**FFUJ_12239_C-MT**	**T**	**E**	**F**	**L**	**E**	**K**	**T**	**M**	**R**	**N**	**V**	**R**	**T**	**L**	**L**	**K**	**P**	**G**	**G**	**Y**	**L**	**C**	**L**	**L**	**E**	**C**	**T**	**G**	**-**	**-**
**ACLA_078660_C-MT**	**T**	**H**	**S**	**L**	**E**	**N**	**T**	**L**	**R**	**Q**	**C**	**R**	**K**	**L**	**L**	**R**	**P**	**G**	**G**	**R**	**L**	**V**	**L**	**L**	**E**	**I**	**T**	**R**	**-**	**-**
**B1GVX7_C-MT**	**T**	**R**	**N**	**L**	**G**	**V**	**T**	**L**	**G**	**N**	**V**	**R**	**S**	**L**	**L**	**K**	**P**	**G**	**G**	**Y**	**L**	**L**	**L**	**N**	**E**	**K**	**T**	**G**	**P**	**-**
**A0JJU1_C-MT**	**T**	**K**	**S**	**L**	**T**	**V**	**T**	**M**	**R**	**N**	**T**	**R**	**K**	**L**	**L**	**K**	**P**	**G**	**G**	**Q**	**L**	**L**	**L**	**L**	**E**	**V**	**T**	**S**	**-**	**-**
**S0EET5_C-MT**	**T**	**P**	**D**	**L**	**E**	**K**	**T**	**M**	**A**	**H**	**A**	**R**	**S**	**L**	**L**	**K**	**P**	**G**	**G**	**Q**	**M**	**V**	**I**	**L**	**E**	**I**	**T**	**H**	**K**	**-**

## Supplementary Materials

The following are available online at https://www.mdpi.com/1660-3397/17/11/606/s1, Figure S1: The HR-ESIMS data of **1**; Figure S2: The ^1^H NMR spectrum of **1** (CD_3_OD, 800 MHz); Figure S3: The ^13^C NMR spectrum of **1** (CD_3_OD, 200 MHz); Figure S4: The ^1^H-^1^H COSY spectrum of **1** (CD_3_OD); Figure S5: The HSQC spectrum of **1** (CD_3_OD); Figure S6: The HMBC spectrum of **1** (CD_3_OD); Figure S7: The ROESY spectrum of **1** (CD_3_OD); Figure S8: The ^1^H NMR spectrum of the (*R*)-MTPA esterification of compound **1**; Figure S9: The TOCSY spectrum of the (*R*)-MTPA esterification of compound **1**; Figure S10: The ^1^H NMR spectrum of the (*S*)-MTPA esterification of compound **1**; Figure S11: The TOCSY spectrum of the (*S*)-MTPA esterification of compound **1**; Figure S12: Media compositions for *C. sphaerospermum* SW67; Figure S13: Comparative total ion chromatogram (positive mode) of culture extracts obtained from SW67; Figure S14: Comparative selected ion chromatogram (*m/z* 271.15) of culture extracts obtained from SW67; Figure S15: LC/MS analysis of extracts obtained from ^13^C-sublemented cultures; Figure S16: The ^1^H NMR spectrum of **2** (CD_3_OD, 800 MHz); Figure S17: The LC/MS data of **2**; Figure S18: The ^1^H NMR spectrum of **3** (CD_3_OD, 800 MHz); Figure S19: The LC/MS data of **3**; Figure S20: The ^1^H NMR spectrum of **4** (CD_3_OD, 800 MHz); Figure S21: The LC/MS data of **4**; Figure S22: Calculated ECD spectra of **1Aa**, **1Ab**, **1Ba**, and **1Bb** and experimental ECD spectrum of **1**; Table S1: Structures of reported hybrid polyketides containing the tetramic acid moiety; Table S2: Gibbs free energies and Boltzmann distribution of conformers **1Aa**; Table S3: Gibbs free energies and Boltzmann distribution of conformers **1Ab**; Table S4: Gibbs free energies and Boltzmann distribution of conformers **1Ba**; Table S5: Gibbs free energies and Boltzmann distribution of conformers **1Bb**; Table S6: Coordinates of the conformers; Table S7: Detailed analysis of the gene NRPS/PKS hybdrid gene cluster; Table S8: List of NR-PKSs used for alignment of unknown ClsI and CluI domains; Table S9: List of NR-PKSs related to known polyketides used for phylogenetic analysis of fungal product-release enzymes.
