# Supplementary material for: Hybrid Polyketides from a Hydractinia-Associated Cladosporium sphaerospermum SW67 and Their Putative Biosynthetic Origin
Source: Mar Drugs. 2019 Oct 24;17(11):606. doi: 10.3390/md17110606 (PMC6891565; doi:10.3390/md17110606)
Supplement: Supplementary file 1 [file marinedrugs-17-00606-s001.pdf]

Supporting Information

Hybrid Polyketides from a *Hydractinia*-associated *Cladosporium sphaerospermum* SW67 and Their Putative Biosynthetic Origin

Seoung Rak Lee [1], Dahae Lee [1], Hee Jeong Eom [1], Maja Rischer [2], Yoon-Joo Ko [3], Ki Sung Kang [4], Chung Sub Kim [5,6], Christine Beemelmanns [2,*] and Ki Hyun Kim [1,*]

[1] School of Pharmacy, Sungkyunkwan University, Suwon 16419, Republic of Korea
[2] Leibniz Institute for Natural Product Research and Infection Biology e.V., Hans-Knöll-Institute (HKI), Jena, Germany
[3] Laboratory of Nuclear Magnetic Resonance, National Center for Inter-University Research Facilities (NCIRF), Seoul National University, Gwanak-gu, Seoul 08826, Republic of Korea
[4] College of Korean Medicine, Gachon University, Seongnam 13120, Republic of Korea
[5] Department of Chemistry, Yale University, New Haven, CT 06520, USA; chungsub.kim@yale.edu (C.S.K.)
[6] Chemical Biology Institute, Yale University, West Haven, CT 06516, USA

Figure S1. The HR-ESIMS data of **1**……………….……………………………………………………………………S3
Figure S2. The ^{1}H NMR spectrum of **1** (CD_3OD, 800 MHz)..…..………………………………………………………….S4
Figure S3. The ^{13}C NMR spectrum of **1** (CD_3OD, 200 MHz)..………………………………………….…………………S5
Figure S4. The ^{1}H-^{1}H COSY spectrum of **1** (CD_3OD)..………………………………………….………….………………S6
Figure S5. The HSQC spectrum of **1** (CD_3OD)….………………………………………………………………………………S7
Figure S6. The HMBC spectrum of **1** (CD_3OD)…………………………………………………………………………………S8
Figure S7. The ROESY spectrum of **1** (CD_3OD)…………………………………………………………………………………S9
Figure S8. The ^{1}H NMR spectrum of the (*R*)-MTPA esterification of compound **1**.………………………………………………S10
Figure S9. The TOCSY spectrum of the (*R*)-MTPA esterification of compound **1**.……………………………………………………S11
Figure S10. The ^{1}H NMR spectrum of the (*S*)-MTPA esterification of compound **1**.………………………………………………S12
Figure S11. The TOCSY spectrum of the (*S*)-MTPA esterification of compound **1**.……………………………………………………S13
Figure S12. Media compositions for *C. sphaerospermum* SW67………………………………………………………………………S14
Figure S13. Comparative total ion chromatogram (positive mode) of culture extracts obtained from SW67 ………………………………S16
Figure S14. Comparative selected ion chromatogram (*m/z* 271.15) of culture extracts obtained from SW67………………………………S17
Figure S15. LC/MS analysis of extracts obtained from ^{13}C-sublemented cultures…………………………………………………………S18
Figure S16. The ^{1}H NMR spectrum of **2** (CD_3OD, 800 MHz)..…..…………………………………………………………………..S19
Figure S17. The LC/MS data of **2**..…..……………………………………………………………………………………….…S20
Figure S18. The ^{1}H NMR spectrum of **3** (CD_3OD, 800 MHz)..…..…………………………………………………………………..S21
Figure S19. The LC/MS data of **3**..…..………………………………………………………………………………………..S22
Figure S20. The ^{1}H NMR spectrum of **4** (CD_3OD, 800 MHz)..…..…………………………………………………………………..S23
Figure S21. The LC/MS data of **4**..…..………………………………………………………………………………………..S24
Figure S22. Calculated ECD spectra of **1Aa**, **1Ab**, **1Ba**, and **1Bb** and experimental ECD spectrum of **1**……..……………………………. S25
Table S1. Structures of reported hybrid polyketides containing the tetramic acid moiety…………………………………………………S26-31
Table S2. Gibbs free energies and Boltzmann distribution of conformers **1Aa**………………………………………………………….S32
Table S3. Gibbs free energies and Boltzmann distribution of conformers **1Ab**………………………………………………………….S32
Table S4. Gibbs free energies and Boltzmann distribution of conformers **1Ba**………………………………………………………….S33
Table S5. Gibbs free energies and Boltzmann distribution of conformers **1Bb**……………………………………………………….…S33
Table S6. Coordinates of the conformers………………………………………………………………………………………..…S34-83
Table S7. Detailed analysis of the gene NRPS/PKS hybdrid gene cluster……………………………………………………………..……S84
Table S8. List of NR-PKSs used for alignment of unknown ClsI and CluI domains…………………………………………………….S86
Table S9. List of NR-PKSs related to known polyketides used for phylogenetic analysis of fungal product-release enzymes……………….S87

Figure S1. The HR-ESIMS data of **1**

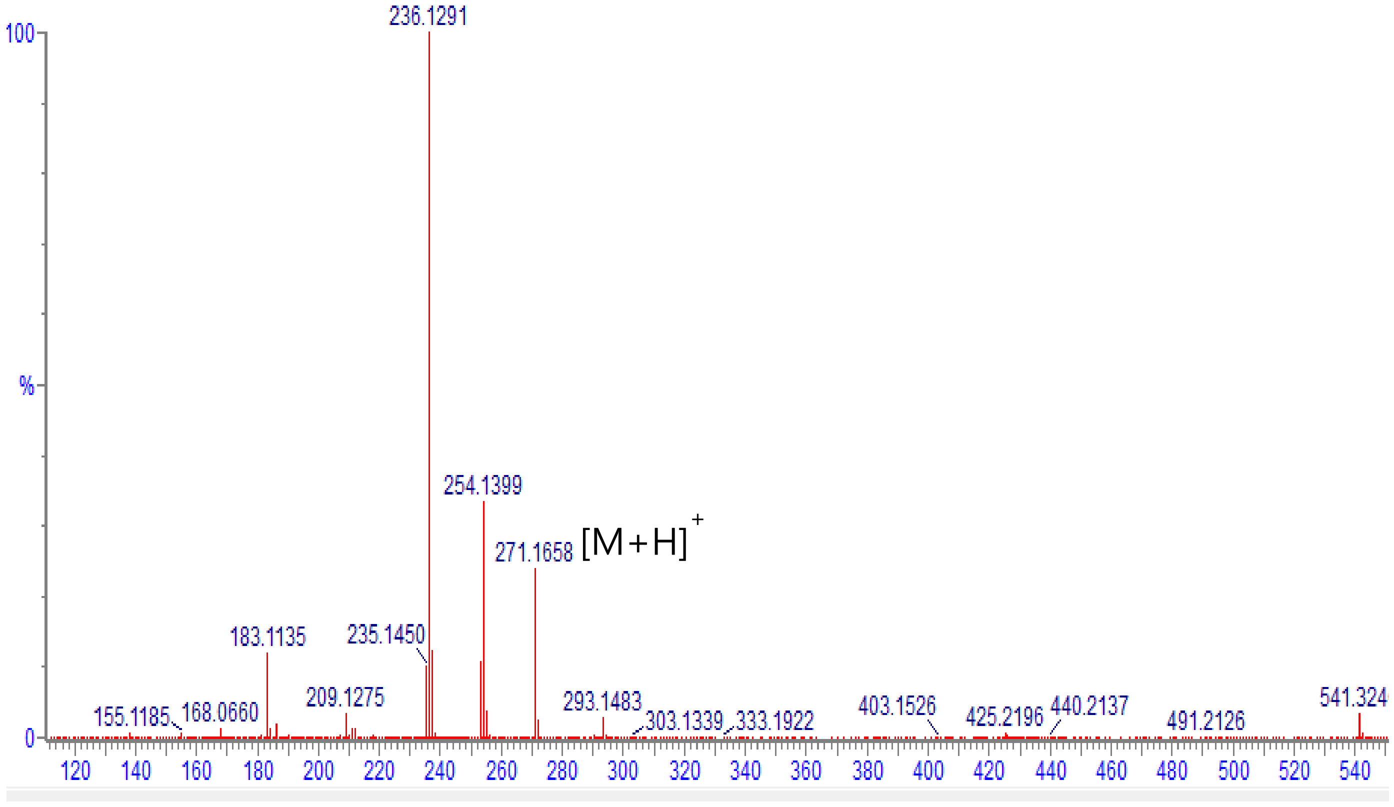

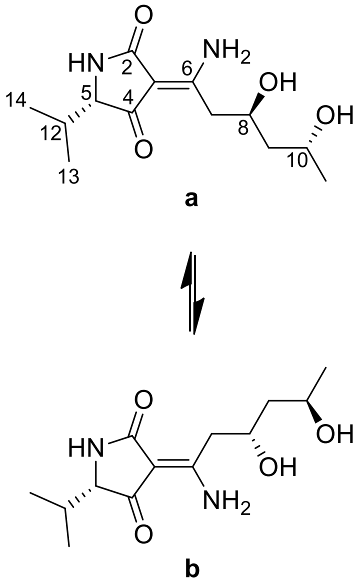

1

$C_{13}H_{22}N_2O_4$
Exact Mass: 270.158
Mol. Wt.: 270.3248

Figure S2. The ^{1}H NMR spectrum of **1** (CD_3OD, 800 MHz)

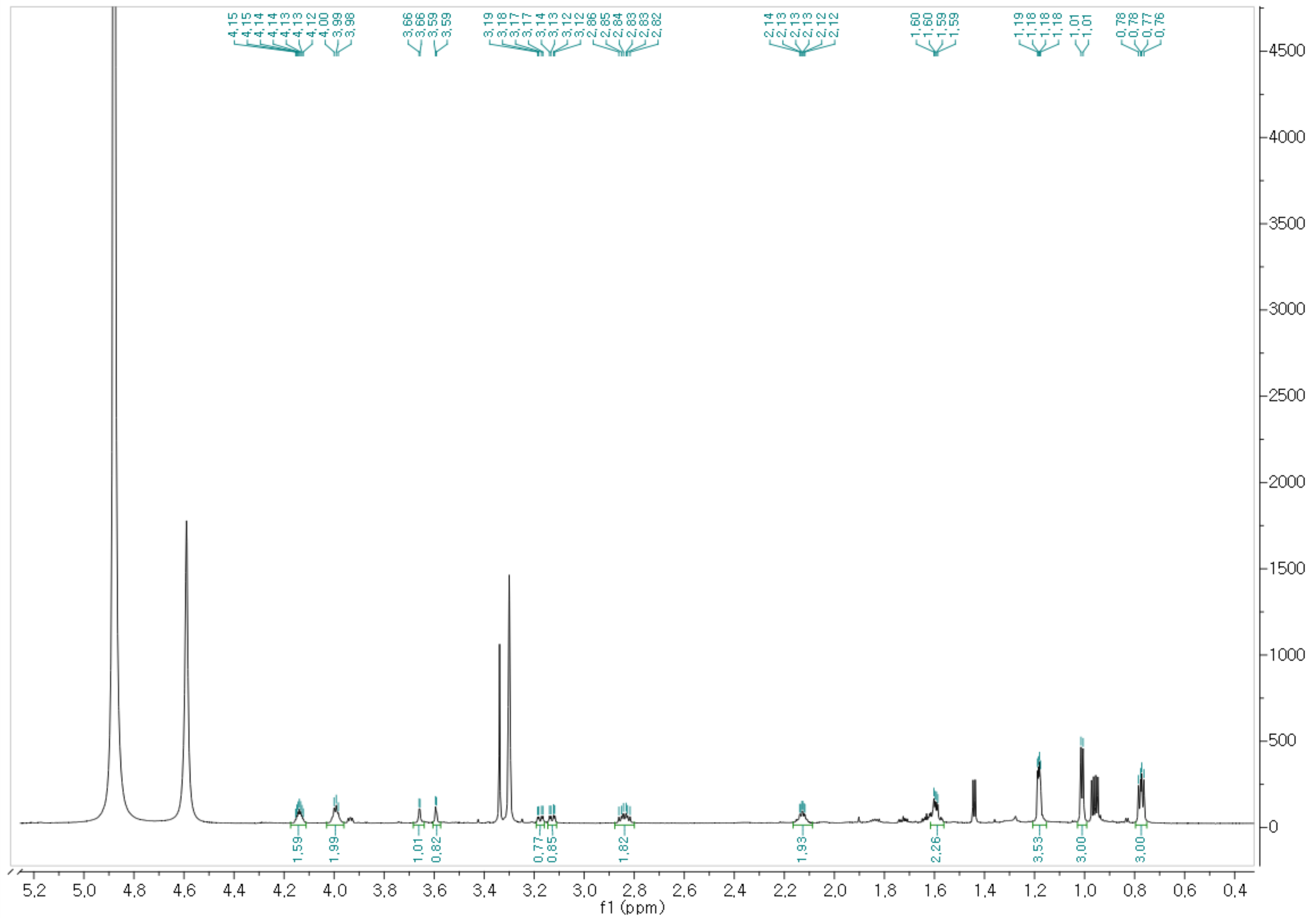

Figure S3. The ^{13}C NMR spectrum of **1** (CD_3OD, 200 MHz)

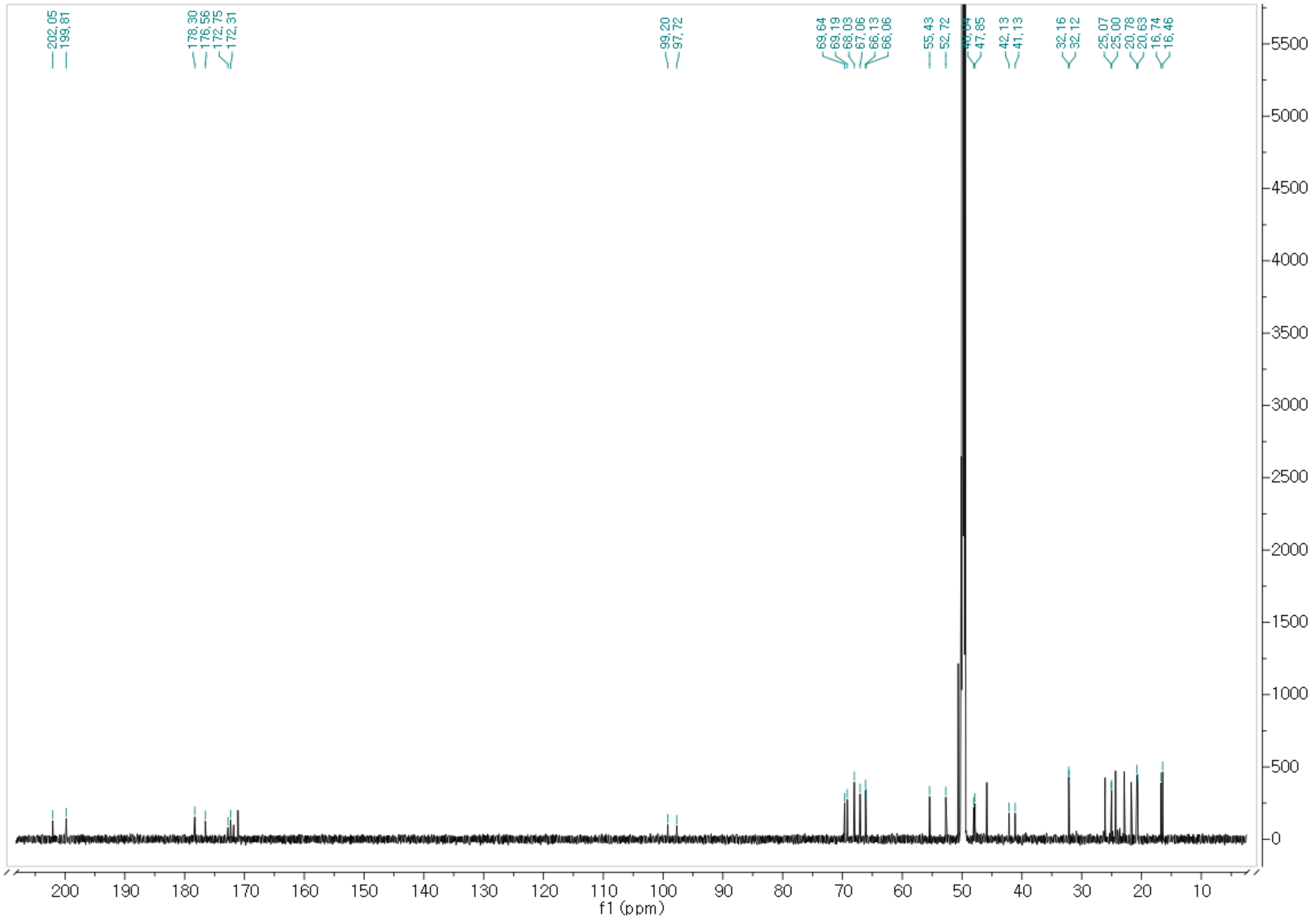

Figure S4. The ^{1}H-^{1}H COSY spectrum of **1** (CD_3OD)

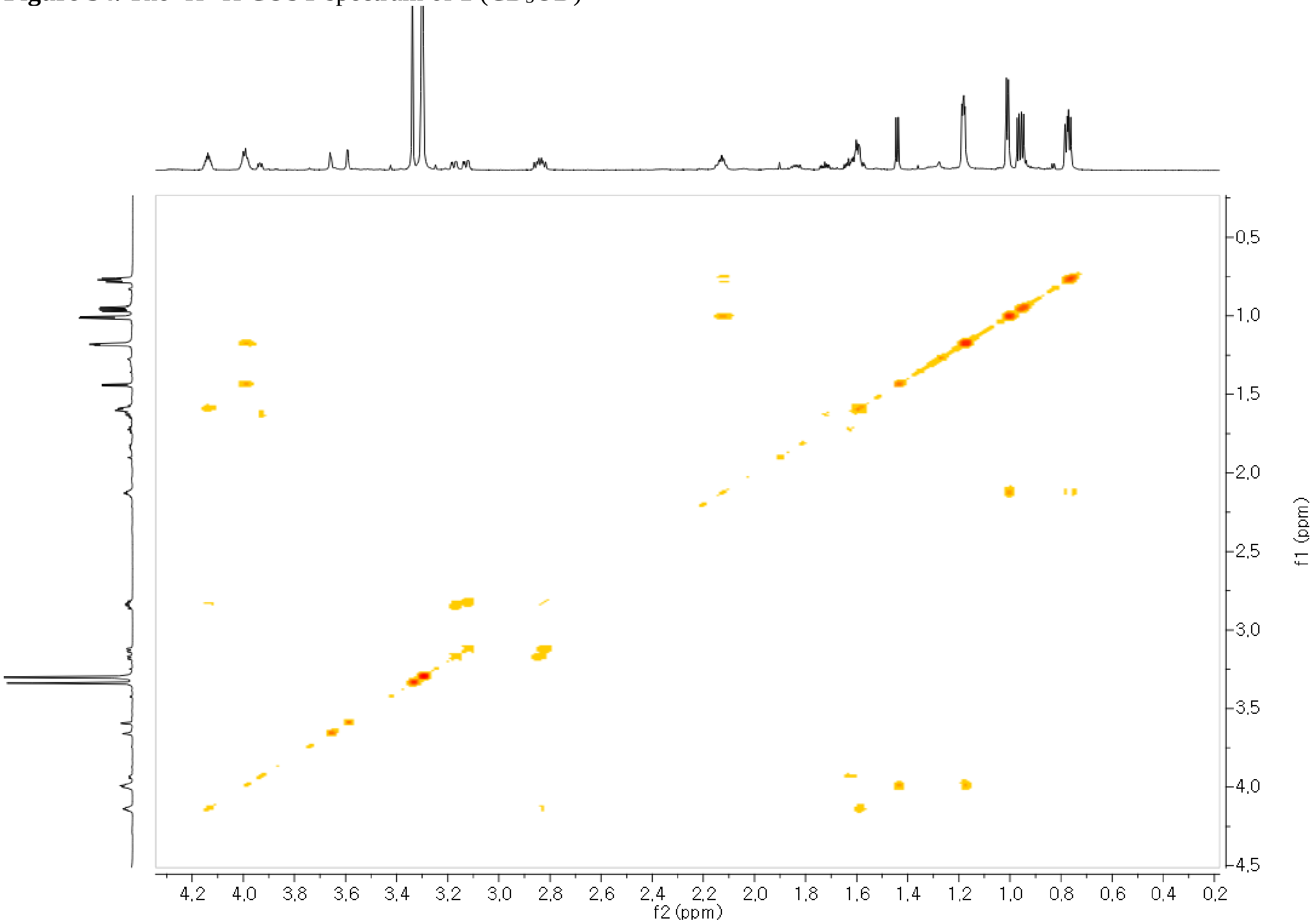

Figure S5. The HSQC spectrum of **1** (CD_3OD)

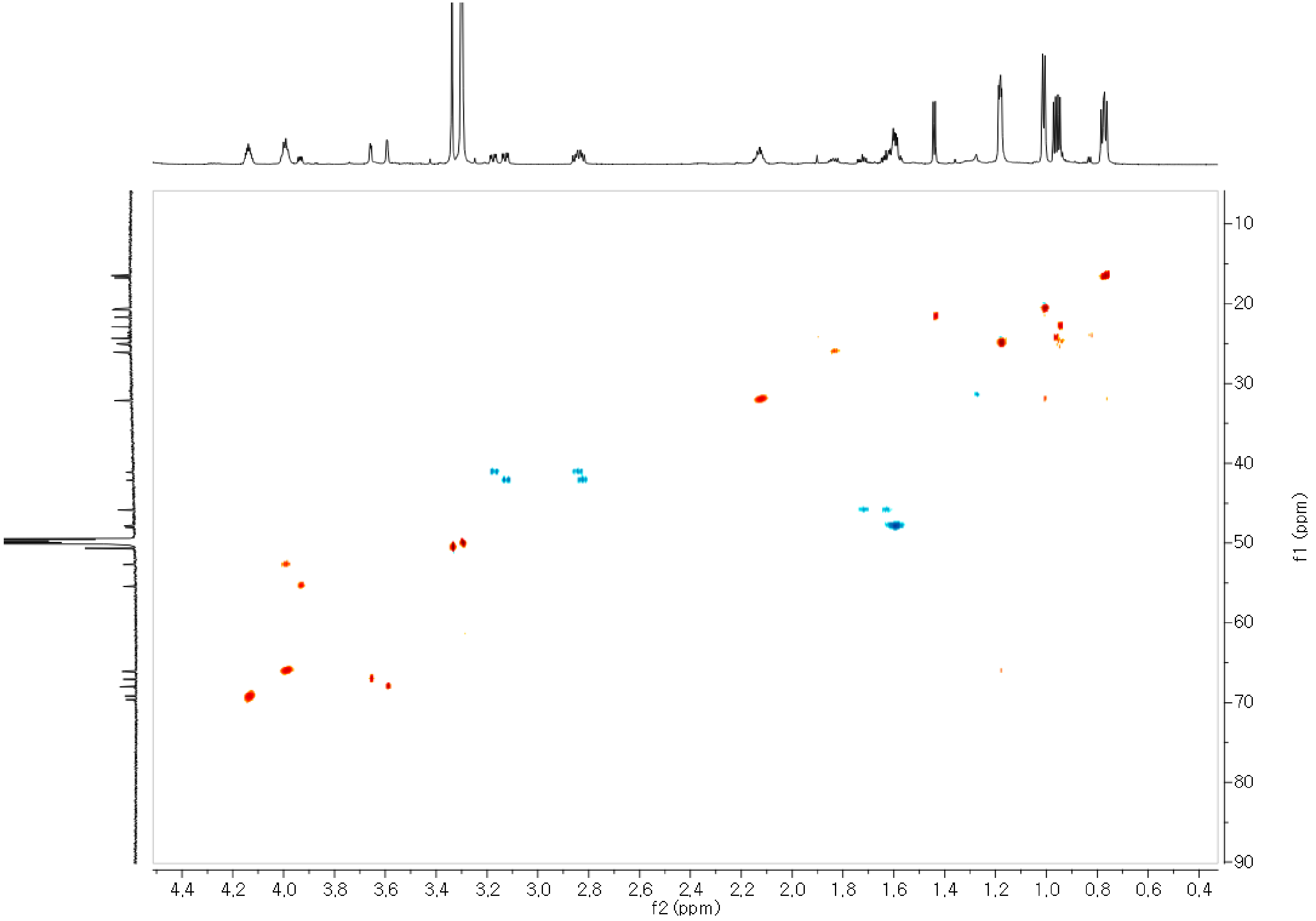

Figure S6. The HMBC spectrum of **1** (CD_3OD)

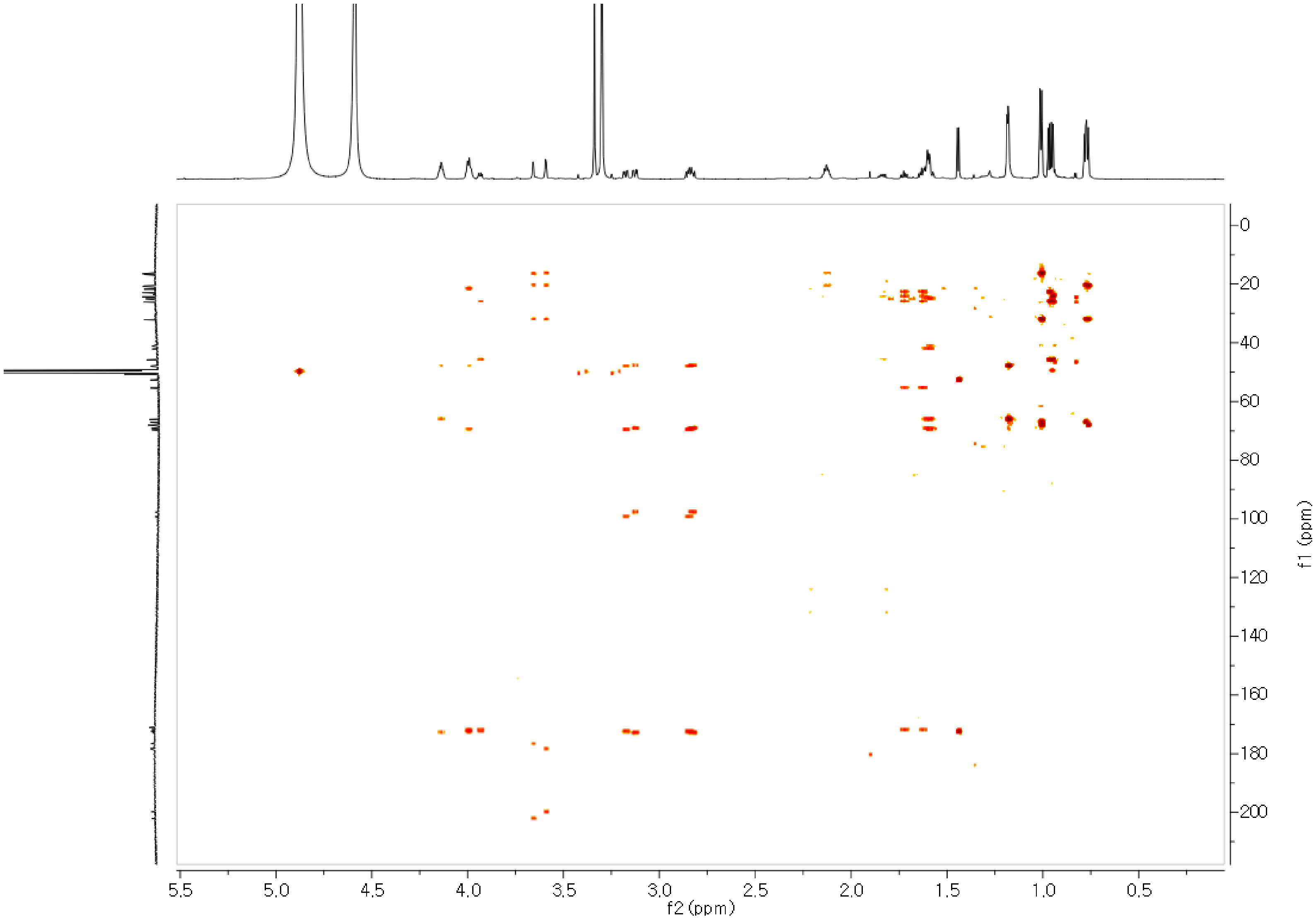

Figure S7. The ROESY spectrum of **1** (CD_3OD)

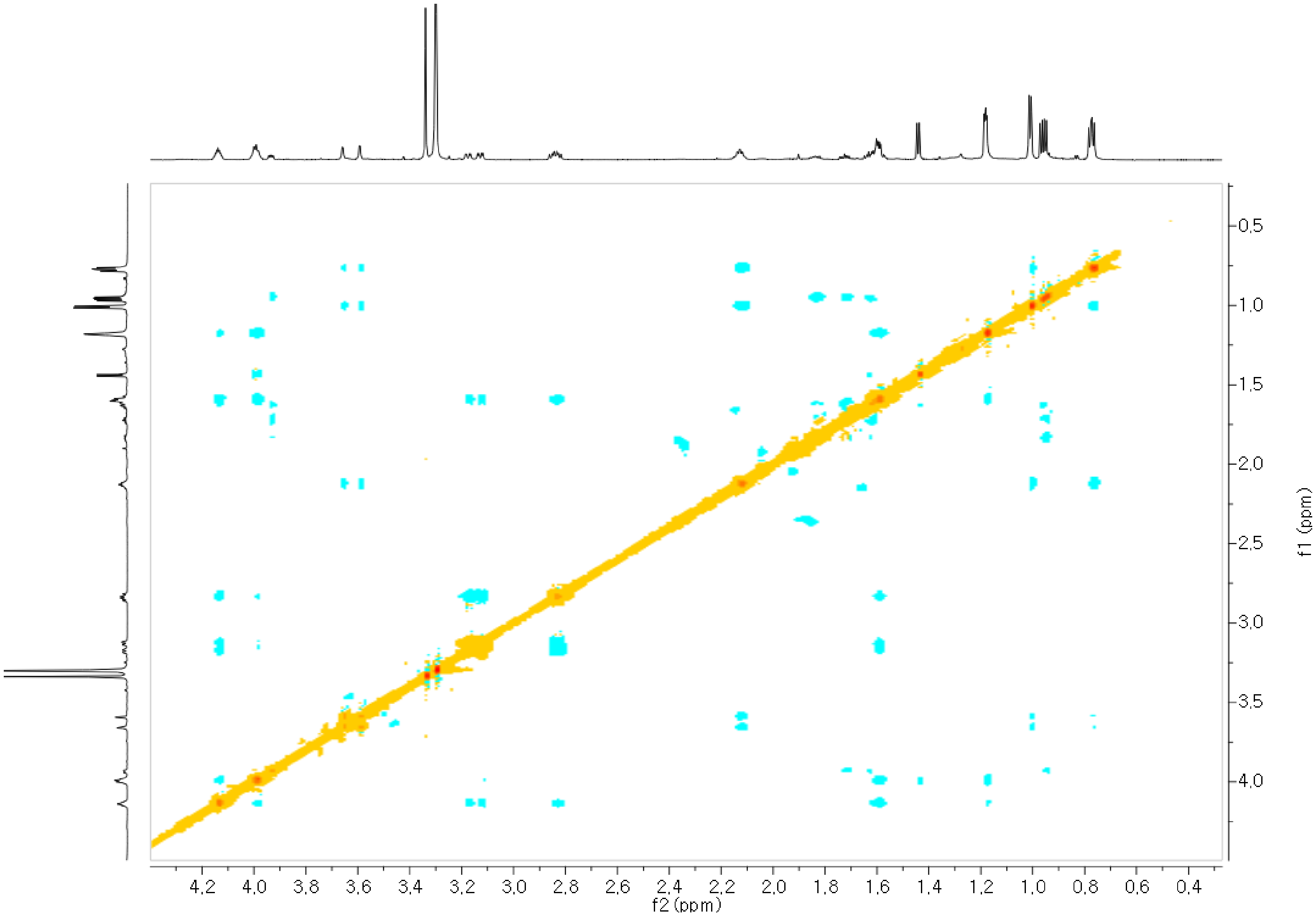

Figure S8. The ^{1}H NMR spectrum of the (*R*)-MTPA esterification of compound **1**

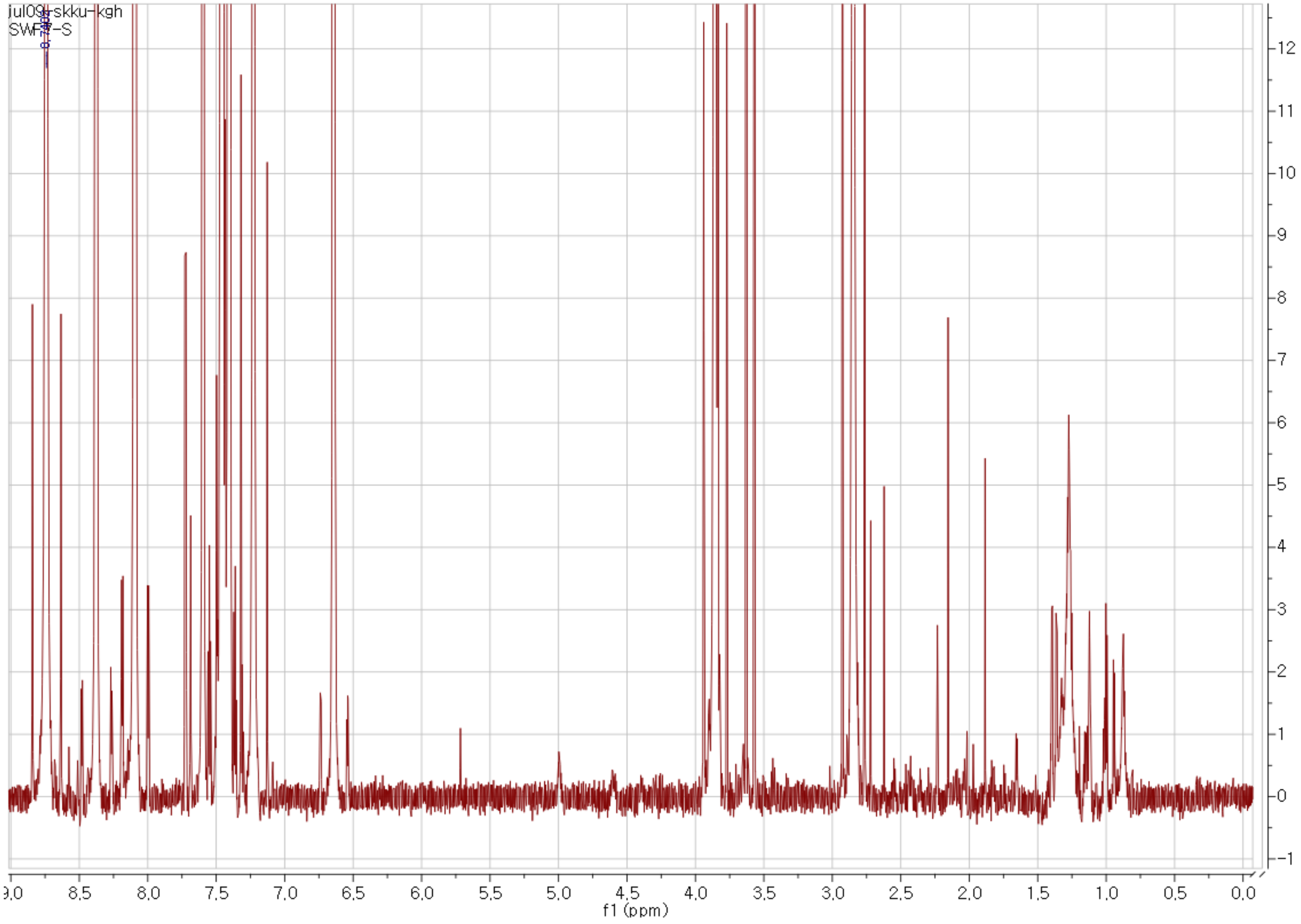

Figure S9. The TOCSY spectrum of the (*R*)-MTPA esterification of compound **1**

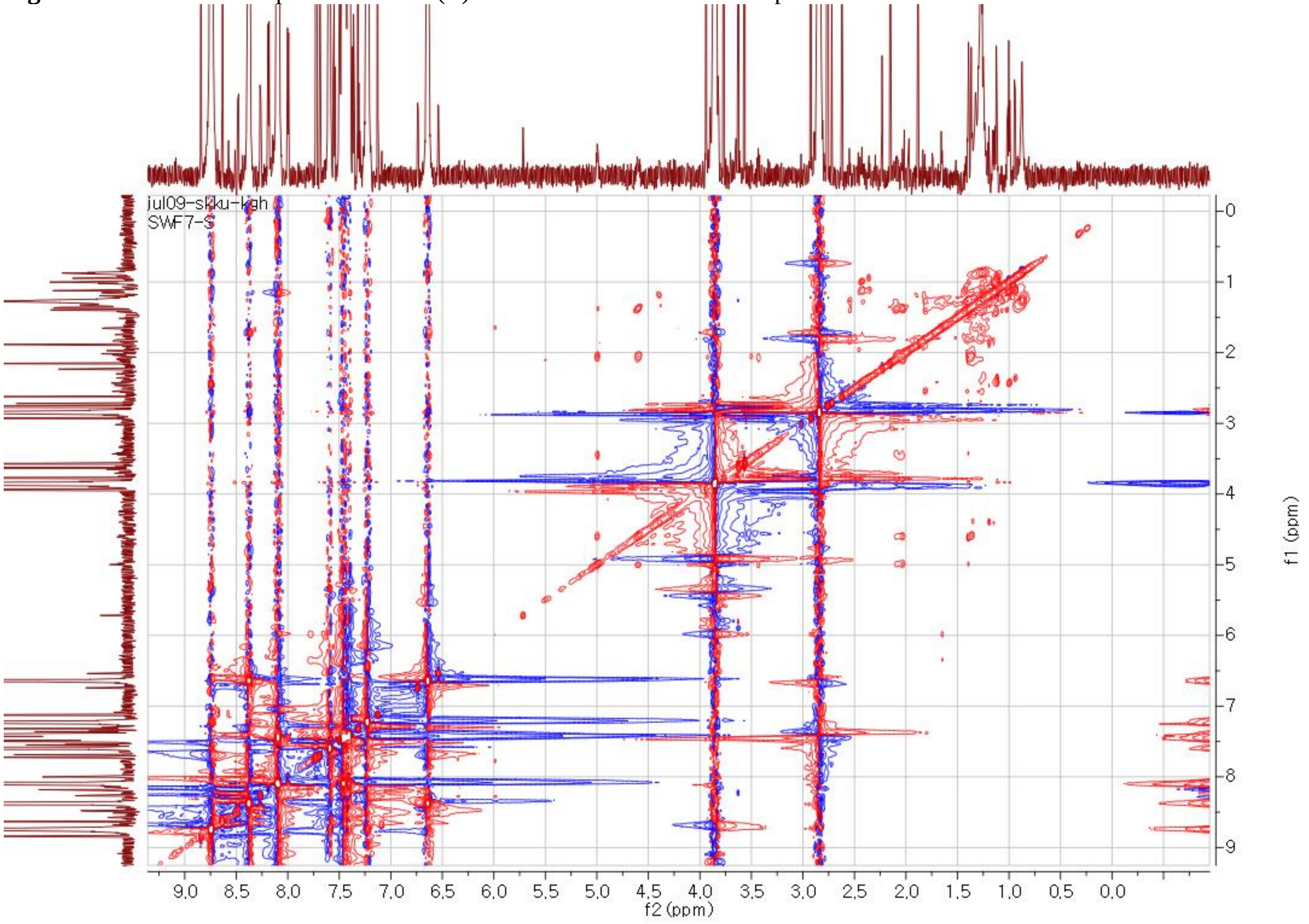

Figure S10. The ^{1}H NMR spectrum of the (*S*)-MTPA esterification of compound **1**

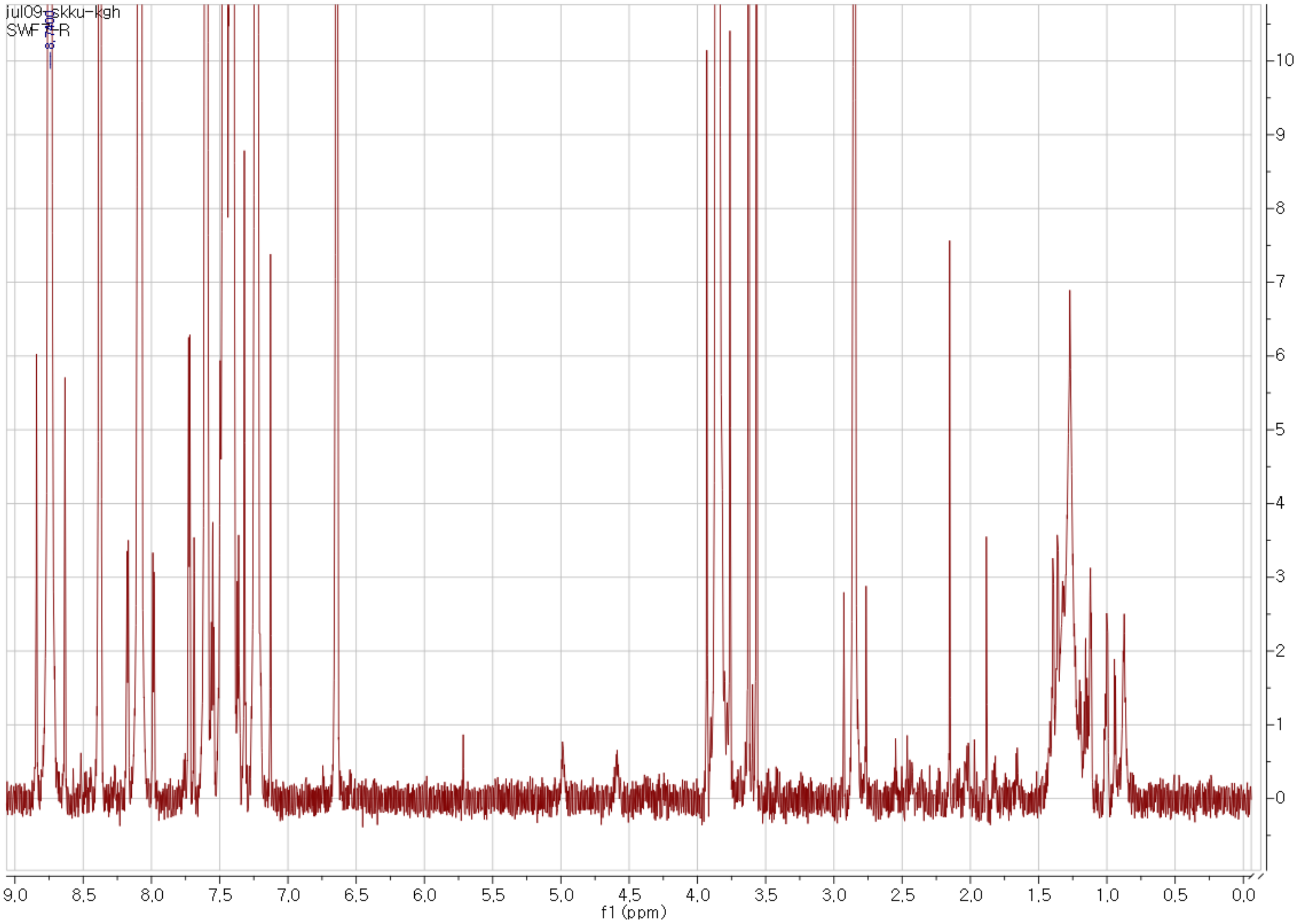

Figure S11. The TOCSY spectrum of the (*S*)-MTPA esterification of compound **1**

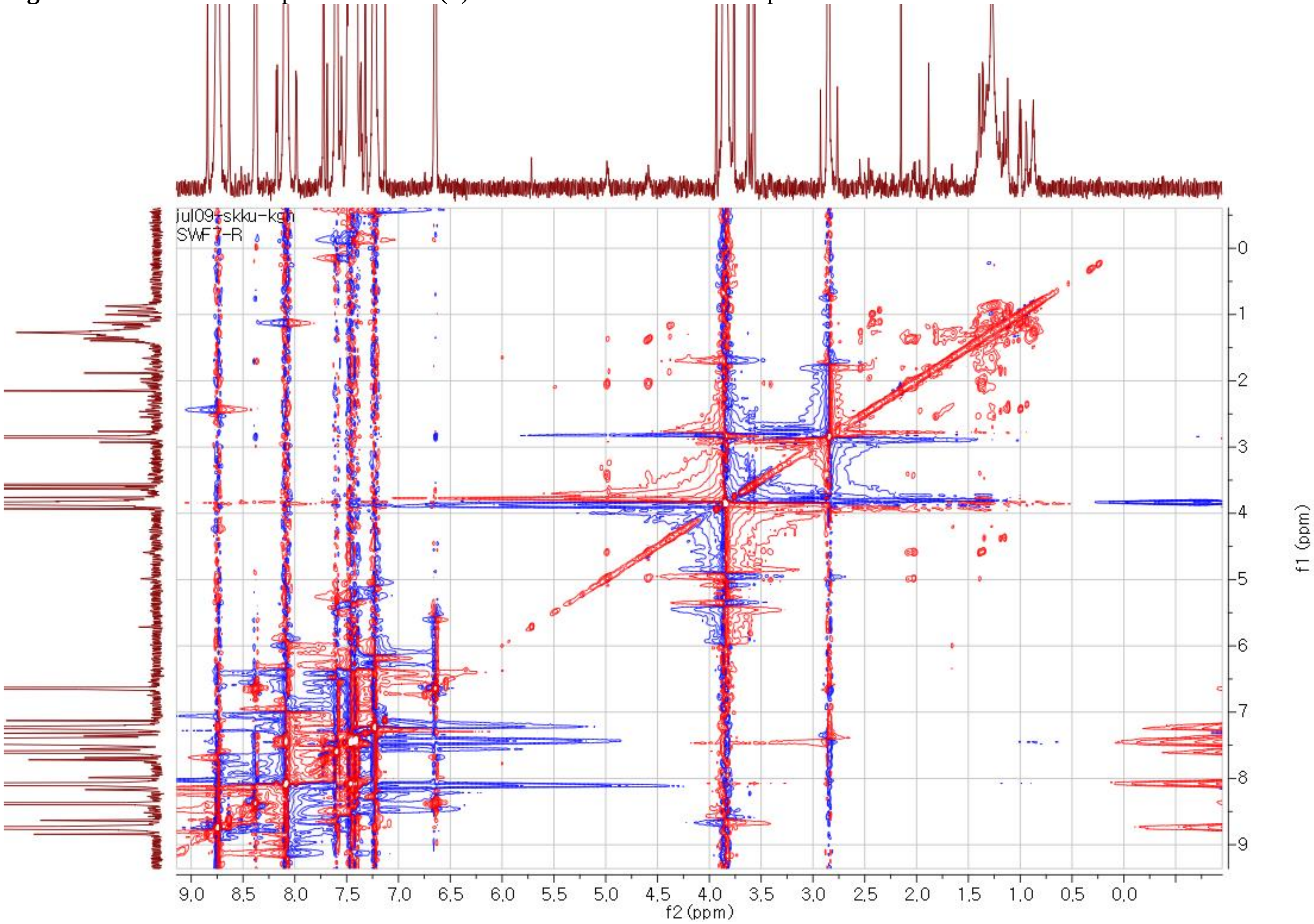

Figure S12. Media compositions for *C. sphaerospermum* SW67

- Potato dextrose broth (PDB): 26.5 g/L potato glucose extract.
- Potato dextrose agar, (PDA): 26.5 g/L potato extract glucose, 20.0 g/L agar.
- Malt extract broth (MEB): 19 g/L malt extract broth.
- Malt extract agar (MEA): 19 g/L malt extract broth, 20.0 g/L agar.
- Glucose peptone yeast agar (GluPe) 1 g/L glucose, 0.5 g/L peptone from casein, 0.1 g/L yeast extract, 20 g/L agar, dissolved in artificial seawater (ASW)
- Wickerham's yeast malt agar (Wick) [1]: 10 g glucose, 3 g/L yeast extract, 5 g peptone from casein, 20 g/L agar, dissolved in ASW

Analytical scale plate cultivation of *C. sphaerospermum* SW67

40 plates (92 mm x 16 mm) of each medium (MEA, PDA, GluPe or Wick) were inoculated with a 100 µL aliquot of a turbid fungal spore suspension of SW67 in sterile PBS. The suspension was evenly distributed over the agar surface and dried. Plates were incubated at 25 °C in the dark for 7, 14, 21 and 28 d. The densely covered agar plates were cut into squares, consolidated, and soaked in MeOH overnight. The solvent was filtered and removed under reduced pressure. The residue was dissolved in 10% MeOH, centrifuged and subjected to SPE on a pre-activated C18 column (Waters, 1 g). After loading, the C18 column was washed with 10%, and then eluted with 100% MeOH. The solvent of the 100% MeOH fraction was removed under reduced pressure and the extract was submitted to UHPLC-MS analysis under the concentration of 1.0 mg/mL in 100% MeOH solution (**Figures S13 and S14**).

LC-MS analysis was attended on an Agilent 1200 series HPLC system with a diode array detector and 6130 Series ESI mass spectrometer using an analytical Kinetex (2.1× 100 mm, 5 µm) (Agilent Technologies, Santa Clara, CA, USA).

UHPLC-MS analysis was performed using the following procedure: 2 µL of sample was injected and analyzed using the following gradient: 0 - 1 min: 10% D, 1 - 7 min: 70% D, 7-10 min: 100% D, 10 – 13.5 min: 10% D (B: ddH_2O with 0.1% formic acid; D: MeCN with 0.1% formic acid) with a flow rate of 0.7 mL/min.

Figure S13. Comparative total ion chromatogram (positive mode) of culture extracts obtained from SW67 cultured on A) MEA; B) PDA, C) GluPe and D) Wick medium. Incubation time: 7, 14, 21 and 28 d at 25 °C in the dark [6].

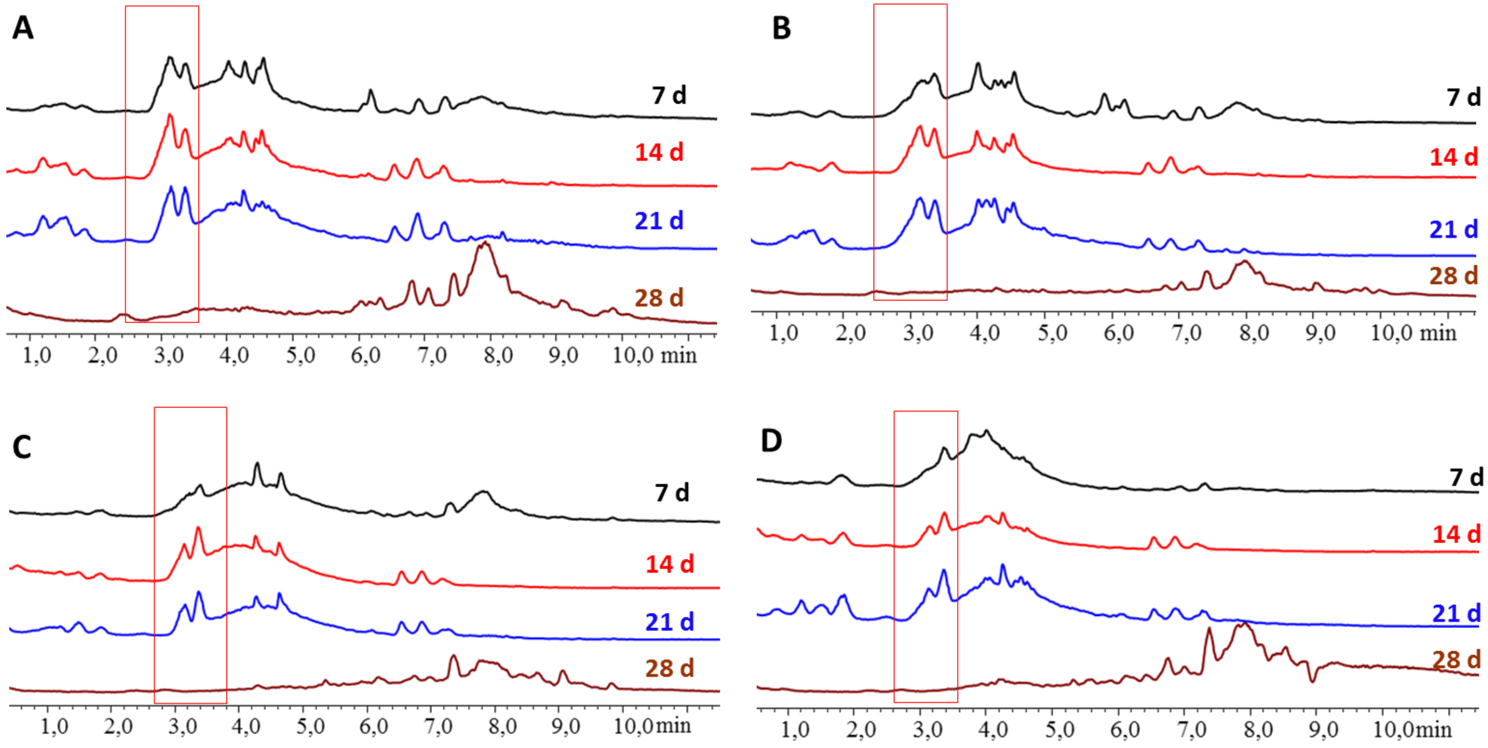

Figure S14. Comparative selected ion chromatogram (*m/z* 271.15) of culture extracts obtained from SW67 cultured on A) MEA; B) PDA, C) GluPe and D) Wick medium. Incubation time: 7, 14, 21 and 28 d at 25 °C in the dark.

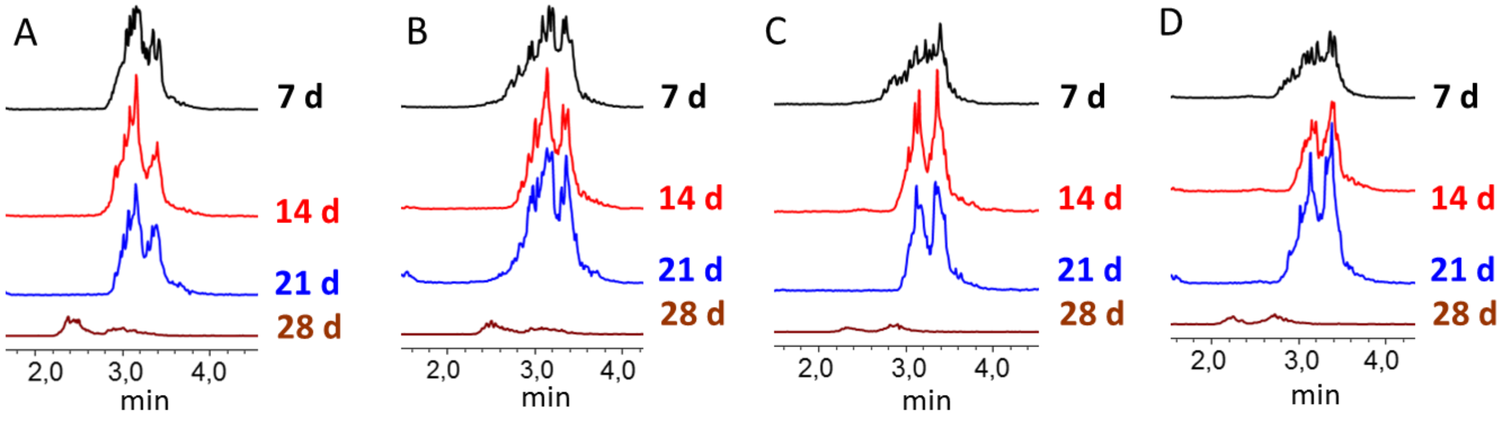

Figure S15. LC/MS analysis of extracts obtained from ^{13}C-sublemented cultures. Asterix (* and **) indicate the respective peaks. Selected MS ion chromatogram (compound **1**, *m/z* =271.15 $[M+H]^{+}$) of SW67 control grown on A) PDA and PDA medium supplemented with B) [1-^{13}C]-acetate, C) [2-^{13}C]-acetate and D) L-[1-^{13}C]-valine.

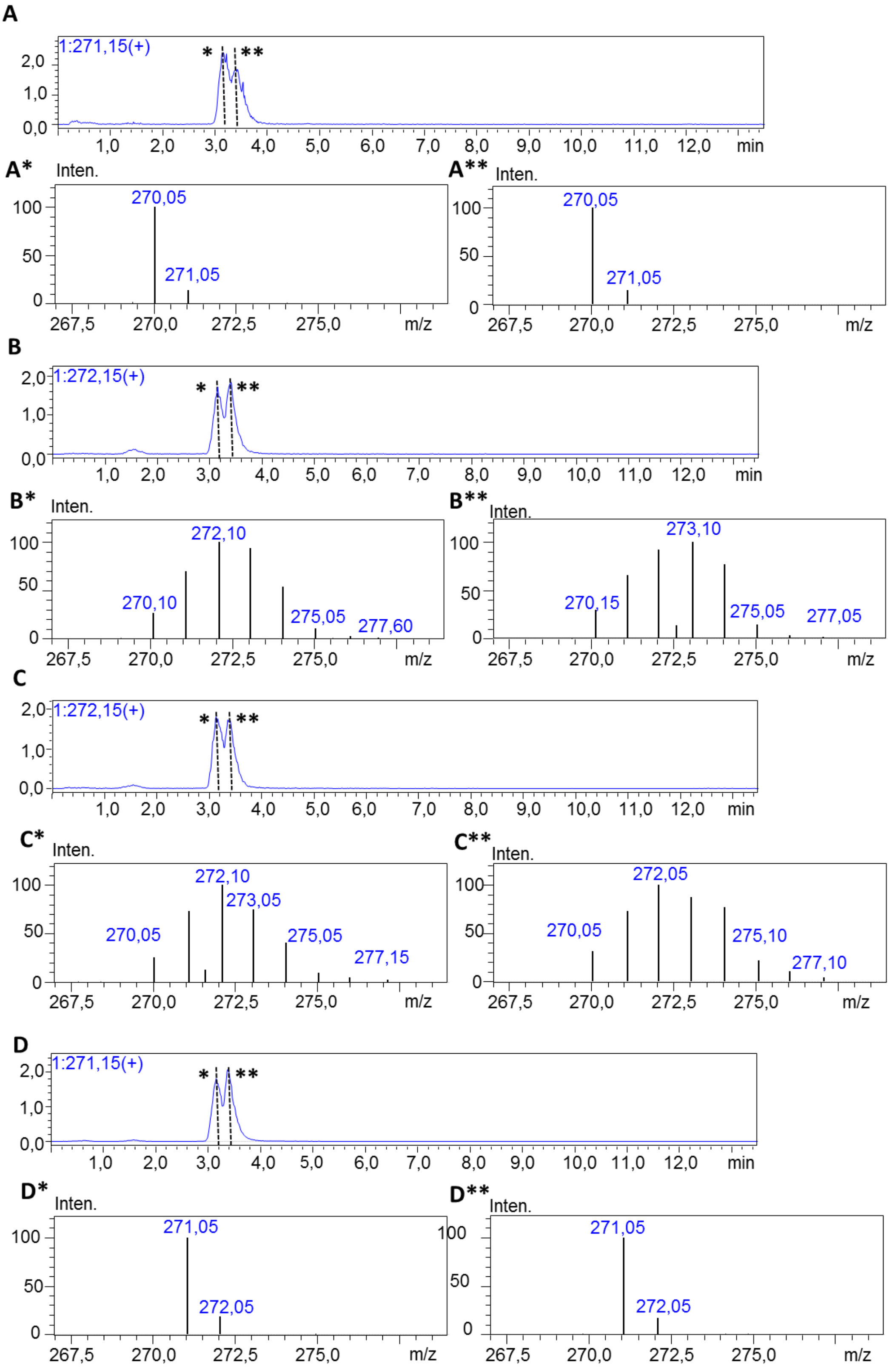

Figure S16. The ^{1}H NMR spectrum of **2** (CD_3OD, 800 MHz)

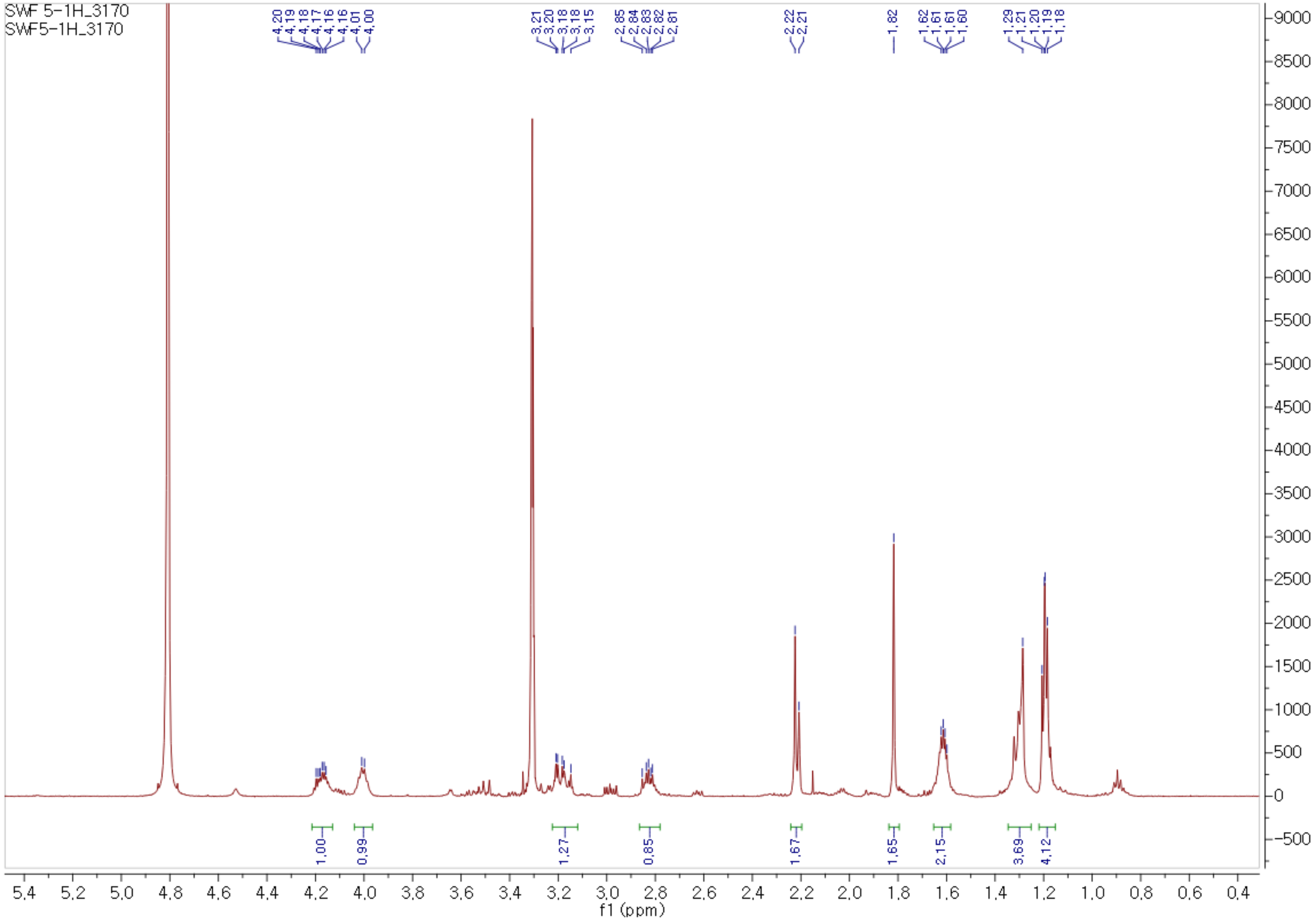

Figure S17. The LC/MS data of **2**

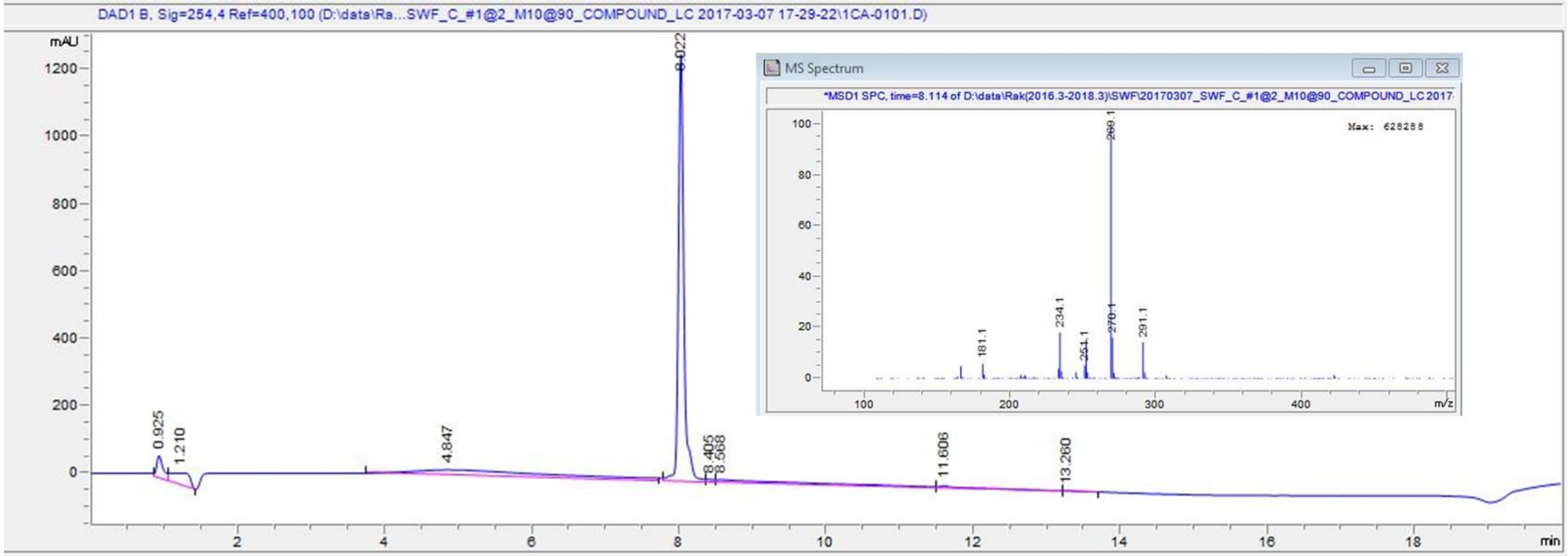

Figure S18. The ^{1}H NMR spectrum of **3** (CD_3OD, 800 MHz)

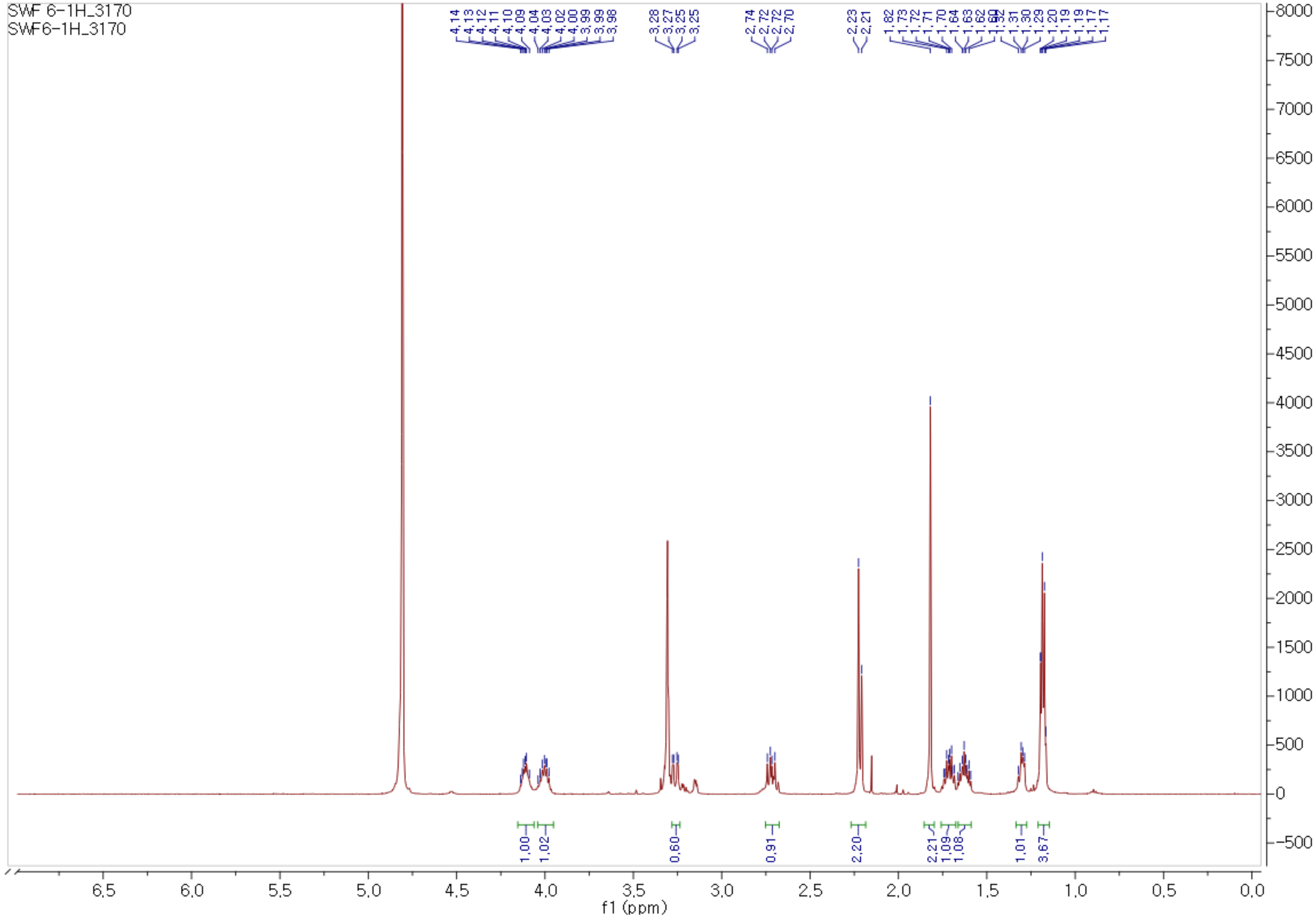

Figure S19. The LC/MS data of **3**

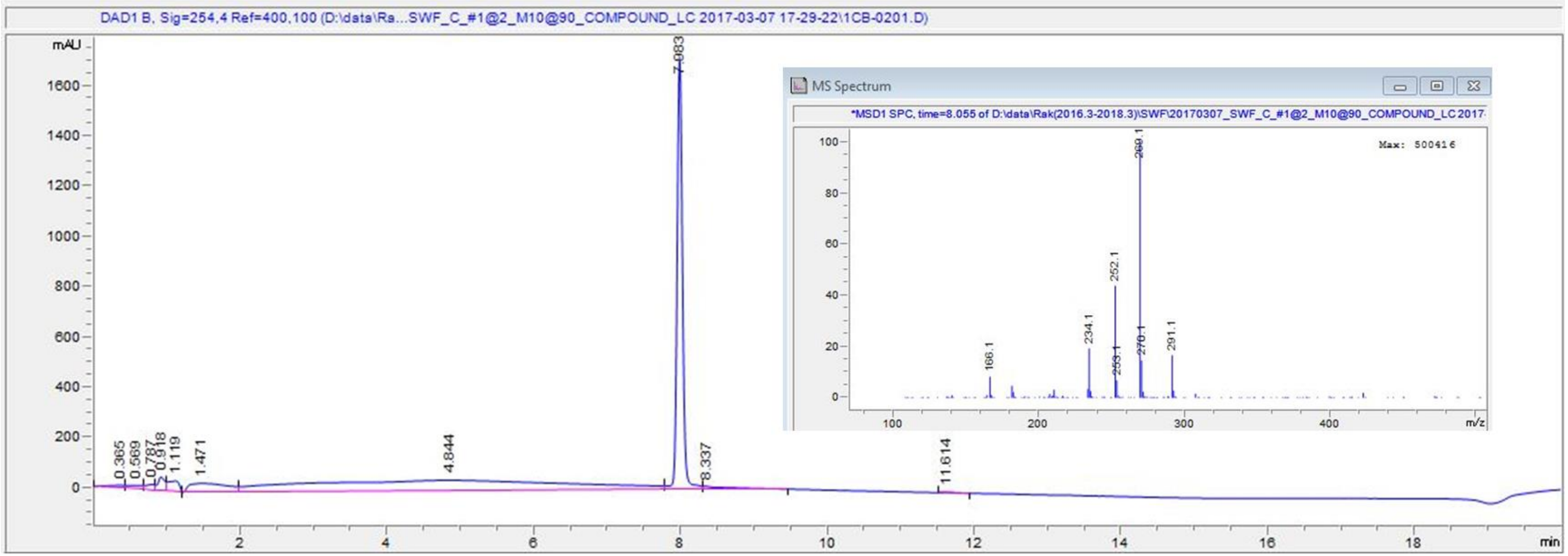

Figure S20. The ^{1}H NMR spectrum of **4** (CD_3OD, 800 MHz)

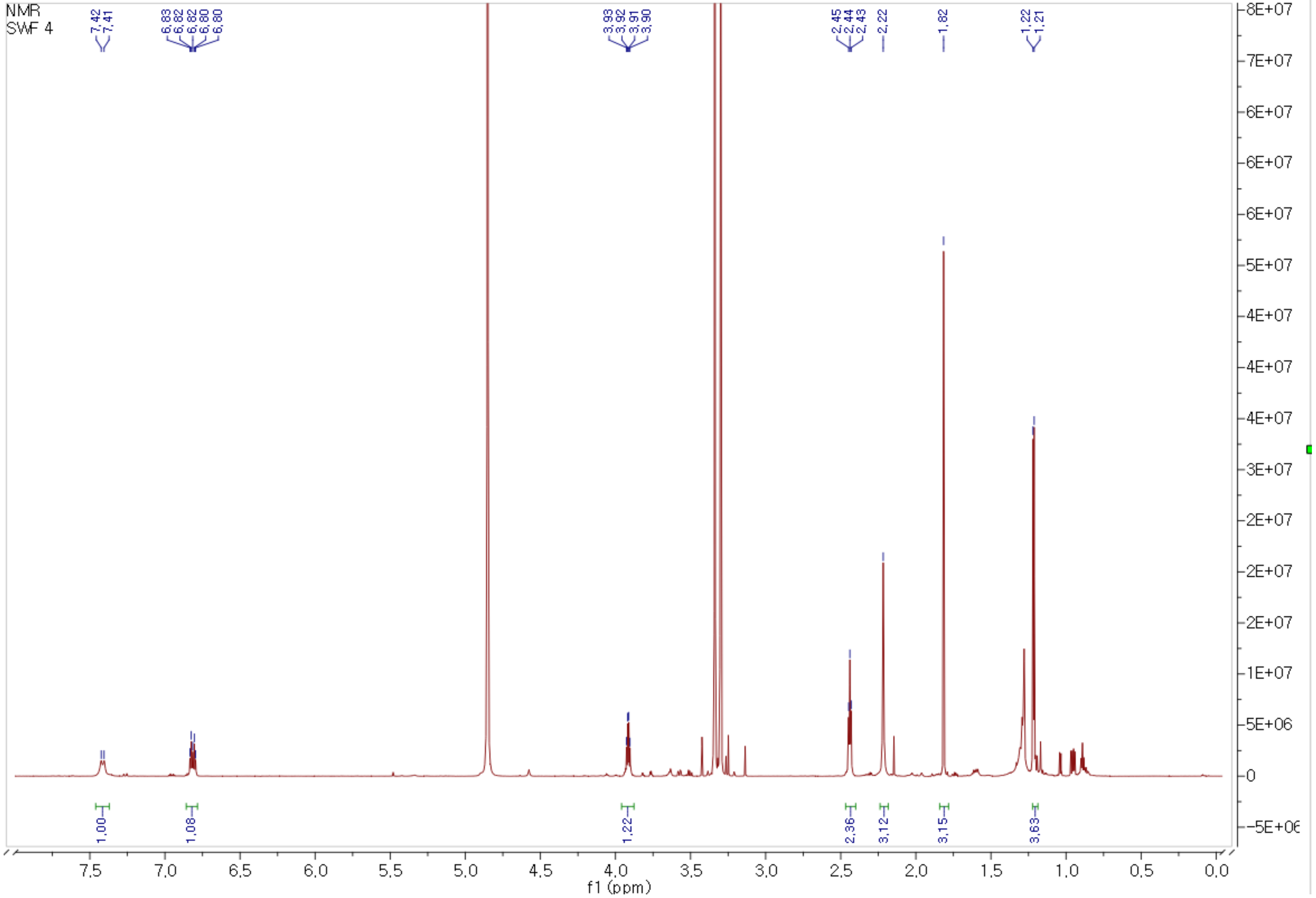

Figure S21. The LC/MS data of **4**

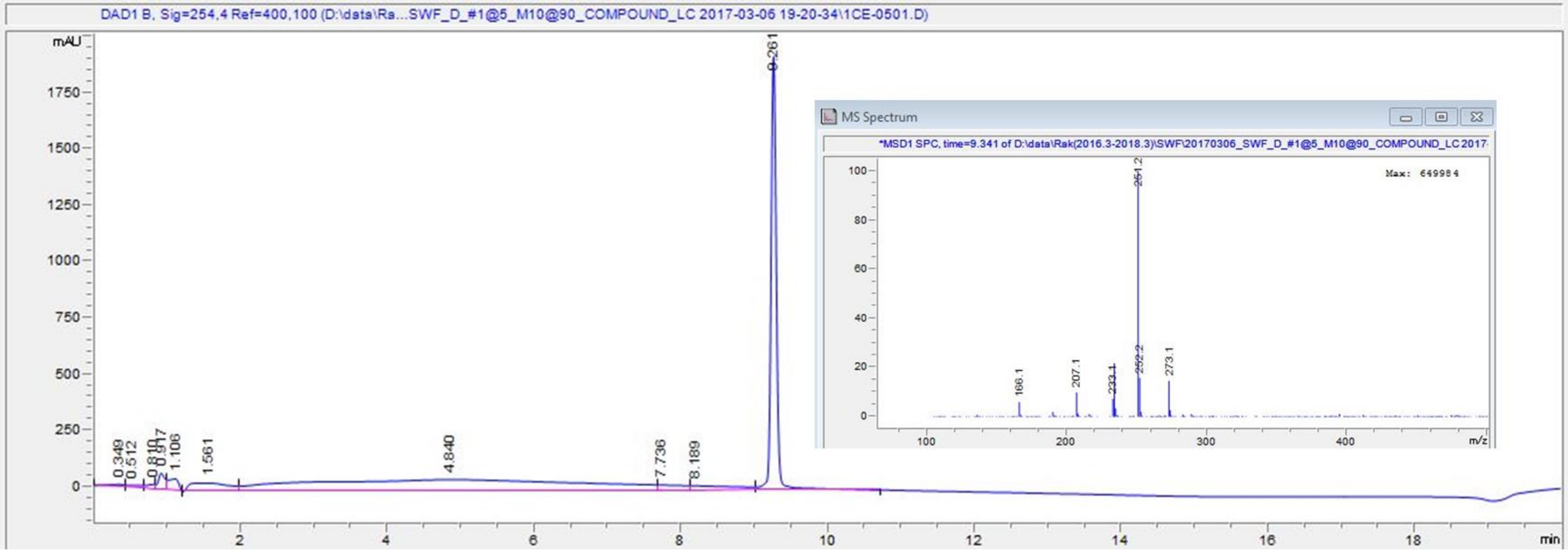

Figure S22. Calculated ECD spectra of **1Aa**, **1Ab**, **1Ba**, and **1Bb** and experimental ECD spectrum of **1**.

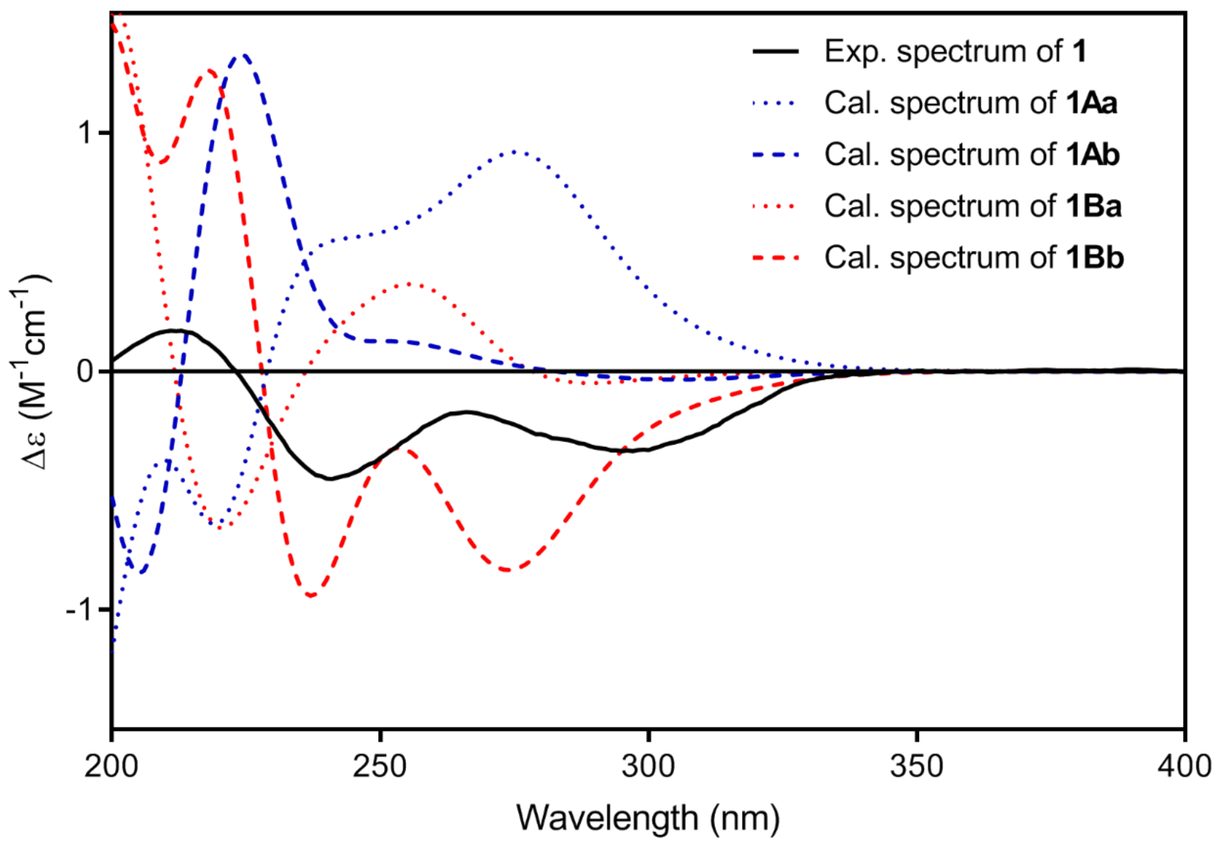

Table S1. Structures of reported hybrid polyketides containing the tetramic acid moiety.

**Compound**		***m/z*** **[M-H]-**	***m/z*** **[M+H]+**	***m/z*** **[M+Na]+**	**Ref**
Cladosporiumin G	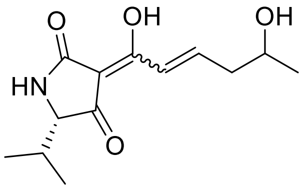	252.1236	254.1392	276.1205	**REF2**
Cladosporiumin H	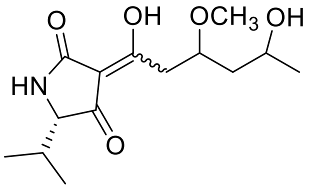	284.1503	286.1654	308.1464	**REF2**
Cladosporiumin I	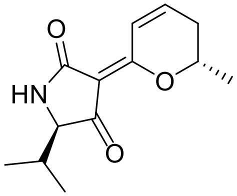	234.1130	236.1287	258.1098	**REF1**
Cladosporiumin J	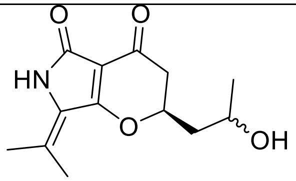	250.1079	252.1236	274.1053	**REF1**
Cladosporiumin K	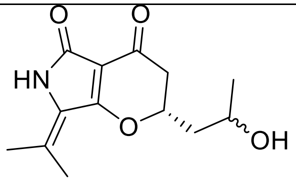				

Cladosporiumin L	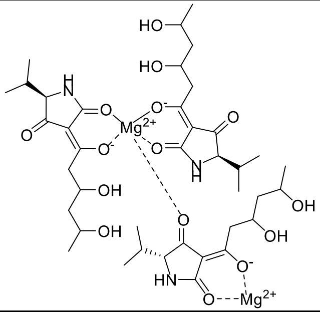		873.3955 [M$^+$]		**REF1**
Cladosporiumin M	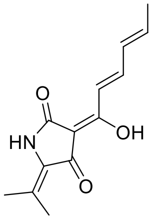	232.0974	234.1130	256.0950	**REF1**
Cladosporiumin N	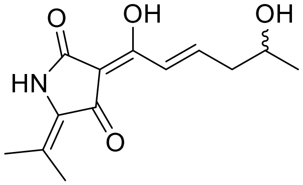	250.1079	252.1236	274.1055	**REF1**
Cladosporiumin O	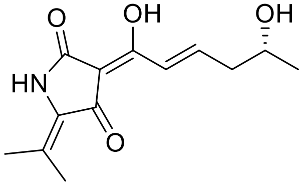				**REF1**
Cladosporiumin E	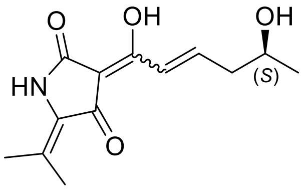				**REF1**

Cladosporiumin F	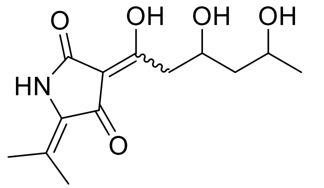	268.1185	270.1341	292.1161	**REF1**
Cladodionen	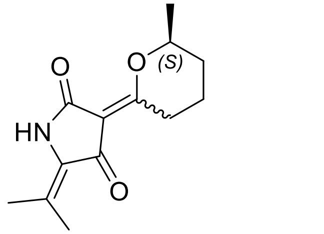	234.1130	236.1287	258.1106	**REF2**
**Cladosin L**	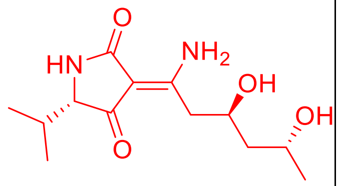	269.1507	271.1658	293.1477	
**Cladosin F**	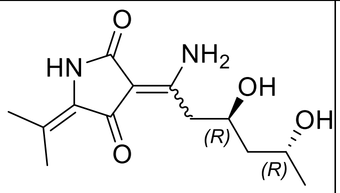	267.1345	269.1501	291.1316	**REF3**
**Cladosin B**	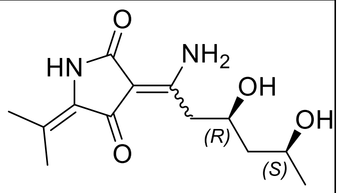				**REF4**

**Cladosin C**	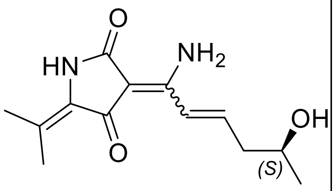	249.1239	251.1396	273.1215	**REF4**
Cladosin D	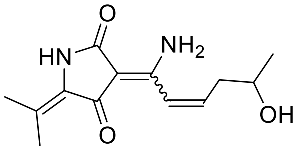				**REF4**
Cladosin A	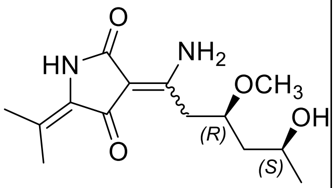	281.1501	283.1658	305.1477	**REF4**
Cladosin G	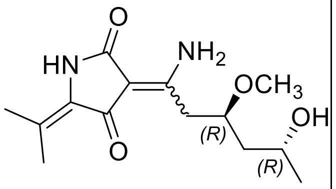				**REF5**
Cladosporiumin D	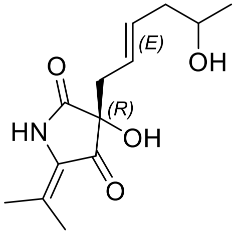	252.1236	254.1392	276.1212	**REF2**

Cladosin E	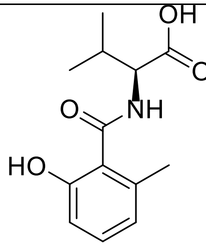	255,15	257.1627	279,14	**REF4**
Cladosporiumin A	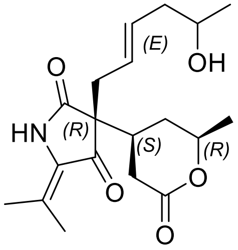	348.1811	350.1969	372.1787	**REF2**
Cladosporiumin B	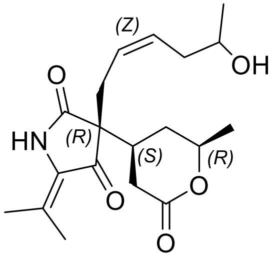				**REF2**
Cladosporiumin C	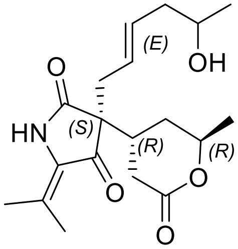				**REF2**

Cladosporiumin I	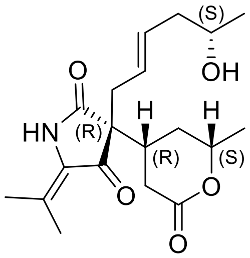	348.1811	350.1967	372.1787	**REF6**
Cladosporiumin J	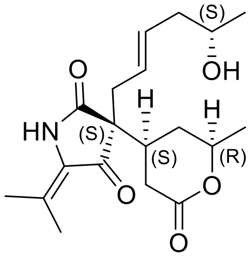				**REF6**
Cladosporicin A	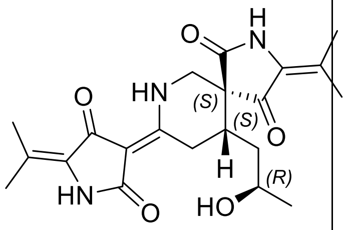	400.1873	402.2029	424.1848	**REF5**

Table S2. Gibbs free energies and Boltzmann distribution of conformers **1Aa**.

Conformers	B3LYP/6-31+G(d,p) Gibbs free energy (298.15 K)		
	G (Hartree)	ΔG (kcal/mol)	Boltmann distribution (%)
**1Aa**-1	-918.847402	0	64.81
**1Aa**-2	-918.846179	0.767444118	17.72
**1Aa**-3	-918.845335	1.297062137	7.24
**1Aa**-4	-918.844954	1.536143256	4.84
**1Aa**-5	-918.844161	2.033758290	2.09
**1Aa**-6	-918.843893	2.201930836	1.57
**1Aa**-7	-918.843644	2.358180701	1.21
**1Aa**-8	-918.842856	2.852658187	0.52

Table S3. Gibbs free energies and Boltzmann distribution of conformers **1Ab**.

Conformers	B3LYP/6-31+G(d,p) Gibbs free energy (298.15 K)		
	G (Hartree)	ΔG (kcal/mol)	Boltmann distribution (%)
**1Ab**-1	-918.848231	0	75.08
**1Ab**-2	-918.846724	0.945656817	15.19
**1Ab**-3	-918.845551	1.681725460	4.38
**1Ab**-4	-918.844673	2.232678801	1.73
**1Ab**-5	-918.844474	2.357553192	1.40
**1Ab**-6	-918.844223	2.515058076	1.07
**1Ab**-7	-918.843695	2.846383092	0.61
**1Ab**-8	-918.843257	3.121232253	0.39
**1Ab**-9	-918.842382	3.670303066	0.15

Table S4. Gibbs free energies and Boltzmann distribution of conformers **1Ba**.

Conformers	B3LYP/6-31+G(d,p) Gibbs free energy (298.15 K)		
	G (Hartree)	ΔG (kcal/mol)	Boltmann distribution (%)
**1Ba**-1	-918.847740	0.000000000	76.00
**1Ba**-2	-918.846254	0.932479117	15.73

**1Ba**-3	-918.844784	1.854918082	3.31
**1Ba**-4	-918.844044	2.319275112	1.51
**1Ba**-5	-918.843937	2.386418629	1.35
**1Ba**-6	-918.843692	2.540158456	1.04
**1Ba**-7	-918.843361	2.747864101	0.73
**1Ba**-8	-918.842606	3.221633773	0.33

Table S5. Gibbs free energies and Boltzmann distribution of conformers **1Bb**.

Conformers	B3LYP/6-31+G(d,p) Gibbs free energy (298.15 K)		
	G (Hartree)	ΔG (kcal/mol)	Boltmann distribution (%)
**1Bb**-1	-918.848120	0.000000000	74.16
**1Bb**-2	-918.846723	0.876630771	16.86
**1Bb**-3	-918.845602	1.580068921	5.14
**1Bb**-4	-918.844468	2.291664694	1.54
**1Bb**-5	-918.844329	2.378888514	1.33
**1Bb**-6	-918.843497	2.900976419	0.55
**1Bb**-7	-918.843219	3.075424060	0.41

Table S6. Coordinates of the conformers

1Aa-1

Center Number	Atomic Number	Atomic Type	Coordinates (Angstroms) X	Y	Z
1	8	0	-1.103304	2.781578	-0.615697
2	6	0	-1.493589	1.609422	-0.497719
3	7	0	-2.759197	1.168251	-0.790464
4	6	0	-2.919603	-0.278081	-0.670523
5	6	0	-4.092025	-0.737086	0.227221
6	6	0	-5.443640	-0.390665	-0.416046
7	6	0	-3.995309	-0.187460	1.657568
8	6	0	-1.537284	-0.722329	-0.148557
9	8	0	-1.265656	-1.914741	0.060286
10	6	0	-0.720534	0.452997	-0.022492
11	6	0	0.586662	0.555024	0.463117
12	6	0	1.328380	-0.617920	1.058385
13	6	0	2.179443	-1.452374	0.066448
14	6	0	3.294994	-0.692466	-0.666946
15	6	0	4.467159	-0.202731	0.182772
16	6	0	5.654705	0.237535	-0.672375
17	8	0	4.003645	0.904099	0.999709
18	8	0	1.381530	-2.048748	-0.943547
19	7	0	1.213867	1.731440	0.451504
20	1	0	-3.048377	-0.734160	-1.663555
21	1	0	2.653092	-2.238786	0.680508
22	1	0	4.786619	-1.012200	0.857414
23	1	0	-3.404383	1.772544	-1.278165
24	1	0	-3.999922	-1.830162	0.269299
25	1	0	-5.524266	-0.798667	-1.430379

26	1	0	-6.269492	-0.800831	0.174999
27	1	0	-5.594230	0.694811	-0.472138
28	1	0	-3.070628	-0.504486	2.150328
29	1	0	-4.026365	0.907546	1.663828
30	1	0	-4.833611	-0.551425	2.261137
31	1	0	0.598546	-1.292312	1.513275
32	1	0	1.990457	-0.245405	1.844676
33	1	0	2.871959	0.149113	-1.228364
34	1	0	3.689611	-1.392633	-1.411801
35	1	0	6.470823	0.619577	-0.047088
36	1	0	6.047948	-0.605385	-1.250025
37	1	0	5.356019	1.027855	-1.368978
38	1	0	4.744684	1.243495	1.518509
39	1	0	0.494084	-2.243425	-0.583432
40	1	0	0.714588	2.530908	0.057198
41	1	0	2.184267	1.785483	0.744358

1Aa-2

Center Number	Atomic Number	Atomic Type	Coordinates (Angstroms) X	Y	Z
1	8	0	-0.979436	2.959044	-0.635598
2	6	0	-1.438992	1.808798	-0.553872
3	7	0	-2.713278	1.443038	-0.896679
4	6	0	-2.984245	0.015185	-0.764691
5	6	0	-4.217964	-0.293378	0.125127
6	6	0	-4.007101	0.115397	1.590205
7	6	0	-4.645590	-1.763268	-0.000762
8	6	0	-1.635288	-0.521596	-0.233663

9	8	0	-1.425517	-1.730748	-0.046867
10	6	0	-0.751017	0.600874	-0.075776
11	6	0	0.548899	0.617101	0.439107
12	6	0	1.207119	-0.604988	1.033891
13	6	0	2.033416	-1.472580	0.049256
14	6	0	3.209682	-0.768694	-0.644043
15	6	0	4.386346	-0.361053	0.242013
16	6	0	5.619782	0.018943	-0.576263
17	8	0	3.967868	0.760466	1.063178
18	8	0	1.228040	-2.006031	-0.989546
19	7	0	1.245658	1.753803	0.457515
20	1	0	-3.148034	-0.438513	-1.752987
21	1	0	2.444900	-2.294493	0.661692
22	1	0	4.639659	-1.196987	0.912477
23	1	0	-3.343851	2.104591	-1.326095
24	1	0	-5.029673	0.328283	-0.283194
25	1	0	-3.673139	1.154617	1.674014
26	1	0	-4.943116	0.017223	2.150340
27	1	0	-3.262377	-0.525838	2.074937
28	1	0	-4.851894	-2.031190	-1.043541
29	1	0	-3.862706	-2.432518	0.366418
30	1	0	-5.558059	-1.942919	0.578225
31	1	0	0.429269	-1.242838	1.460749
32	1	0	1.869829	-0.282226	1.841398
33	1	0	2.850738	0.104255	-1.202135
34	1	0	3.581559	-1.479929	-1.390117
35	1	0	6.440462	0.343577	0.074961
36	1	0	5.977424	-0.838326	-1.155940
37	1	0	5.386687	0.834596	-1.268694
38	1	0	4.713892	1.048839	1.605174
39	1	0	0.320393	-2.147968	-0.655760

40	1	0	0.803127	2.587606	0.066408
41	1	0	2.210327	1.745959	0.773312

1Aa-3

Center Number	Atomic Number	Atomic Type	Coordinates (Angstroms) X	Y	Z
1	8	0	-2.440681	2.804849	-0.076255
2	6	0	-2.150972	1.604448	-0.207211
3	7	0	-3.008151	0.628912	-0.636175
4	6	0	-2.393095	-0.688583	-0.762135
5	6	0	-3.106666	-1.824333	0.007902
6	6	0	-4.474717	-2.135805	-0.618360
7	6	0	-3.229676	-1.537879	1.511299
8	6	0	-0.952370	-0.430403	-0.269428
9	8	0	-0.110793	-1.335704	-0.220800
10	6	0	-0.851289	0.967884	0.062901
11	6	0	0.241945	1.668293	0.578317
12	6	0	1.569914	1.019423	0.846344
13	6	0	2.426170	0.854679	-0.435534
14	6	0	3.683869	0.017052	-0.179697
15	6	0	3.476717	-1.507691	-0.270665
16	6	0	4.786900	-2.255197	-0.038264
17	8	0	2.537845	-1.995691	0.689661
18	8	0	2.854169	2.141843	-0.910543
19	7	0	0.132478	2.981418	0.810794
20	1	0	-2.326324	-0.980880	-1.820917
21	1	0	1.822083	0.364218	-1.210475
22	1	0	3.110594	-1.741967	-1.283731

23	1	0	-3.928998	0.867470	-0.974103
24	1	0	-2.458645	-2.700127	-0.125320
25	1	0	-4.388244	-2.359519	-1.688017
26	1	0	-4.932260	-3.003814	-0.132219
27	1	0	-5.171692	-1.296376	-0.500754
28	1	0	-2.249721	-1.396954	1.978216
29	1	0	-3.828967	-0.639891	1.697452
30	1	0	-3.717923	-2.377372	2.017088
31	1	0	1.416710	0.037383	1.295588
32	1	0	2.145969	1.630582	1.550350
33	1	0	4.110989	0.269256	0.799836
34	1	0	4.418279	0.310414	-0.938510
35	1	0	5.155156	-2.064717	0.975882
36	1	0	4.627984	-3.331961	-0.144309
37	1	0	5.555055	-1.940136	-0.752541
38	1	0	1.632765	-1.863167	0.355846
39	1	0	2.115226	2.583373	-1.349779
40	1	0	-0.768417	3.428762	0.618014
41	1	0	0.905399	3.499395	1.200764

1Aa-4

Center Number	Atomic Number	Atomic Type	Coordinates (Angstroms) X	Y	Z
1	8	0	-2.142909	2.854044	0.126076
2	6	0	-2.146903	1.629844	-0.083301
3	7	0	-3.228084	0.904049	-0.503286
4	6	0	-2.947979	-0.511463	-0.730708
5	6	0	-3.866875	-1.494460	0.030456

6	6	0	-5.300517	-1.446383	-0.520245
7	6	0	-3.842360	-1.276392	1.550127
8	6	0	-1.460189	-0.625478	-0.320881
9	8	0	-0.839431	-1.687939	-0.355247
10	6	0	-1.019440	0.698151	0.067868
11	6	0	0.228157	1.085181	0.544985
12	6	0	1.377116	0.122079	0.679549
13	6	0	2.162449	-0.042841	-0.645890
14	6	0	3.300740	-1.066564	-0.538737
15	6	0	4.487587	-0.682185	0.362774
16	6	0	5.436856	-1.857411	0.574081
17	8	0	5.262448	0.378382	-0.202417
18	8	0	2.736708	1.212478	-1.062396
19	7	0	0.453992	2.370403	0.858323
20	1	0	-3.008517	-0.745699	-1.804166
21	1	0	1.461203	-0.392964	-1.412173
22	1	0	4.109547	-0.366367	1.350189
23	1	0	-4.084581	1.368806	-0.767382
24	1	0	-3.448175	-2.486508	-0.182912
25	1	0	-5.323798	-1.622277	-1.601996
26	1	0	-5.922681	-2.211832	-0.044515
27	1	0	-5.775286	-0.476947	-0.322450
28	1	0	-2.833070	-1.389921	1.958452
29	1	0	-4.207137	-0.277801	1.814706
30	1	0	-4.484516	-2.010392	2.048156
31	1	0	0.985108	-0.858457	0.959094
32	1	0	2.056818	0.476113	1.461890
33	1	0	3.690427	-1.252537	-1.546979
34	1	0	2.862072	-2.006642	-0.183066
35	1	0	5.799764	-2.233728	-0.388471
36	1	0	6.301946	-1.534146	1.160304

37	1	0	4.938861	-2.674178	1.107437
38	1	0	4.648646	1.037497	-0.561256
39	1	0	2.047960	1.800236	-1.398838
40	1	0	-0.323946	3.028072	0.760569
41	1	0	1.341631	2.660321	1.239543

1Aa-5

Center Number	Atomic Number	Atomic Type	Coordinates (Angstroms) X	Y	Z
1	8	0	-2.094315	2.754697	-0.829568
2	6	0	-2.046267	1.531318	-0.620925
3	7	0	-3.014370	0.631244	-0.974470
4	6	0	-2.682389	-0.757624	-0.666718
5	6	0	-3.727888	-1.509945	0.188316
6	6	0	-5.021139	-1.747354	-0.606684
7	6	0	-4.012684	-0.814811	1.527428
8	6	0	-1.307009	-0.623801	0.029771
9	8	0	-0.682552	-1.592577	0.462320
10	6	0	-0.965462	0.783438	0.038118
11	6	0	0.157100	1.394200	0.586769
12	6	0	1.262926	0.612709	1.244331
13	6	0	2.315633	0.105678	0.230566
14	6	0	3.407320	-0.699827	0.936505
15	6	0	4.552038	-1.212587	0.037750
16	6	0	4.079154	-2.038248	-1.164027
17	8	0	5.416330	-0.150464	-0.372799
18	8	0	2.950302	1.213336	-0.445265
19	7	0	0.304473	2.724765	0.498984

20	1	0	-2.523159	-1.327483	-1.594474
21	1	0	1.808374	-0.525601	-0.507854
22	1	0	5.182030	-1.854354	0.665669
23	1	0	-3.803405	0.921996	-1.533235
24	1	0	-3.266519	-2.484128	0.395954
25	1	0	-4.824022	-2.261449	-1.554588
26	1	0	-5.719954	-2.364226	-0.031833
27	1	0	-5.534334	-0.803670	-0.831195
28	1	0	-3.105884	-0.716323	2.132648
29	1	0	-4.431421	0.186002	1.374818
30	1	0	-4.736361	-1.395602	2.108926
31	1	0	0.832340	-0.257191	1.745306
32	1	0	1.769077	1.243420	1.984533
33	1	0	2.922253	-1.557175	1.418996
34	1	0	3.851960	-0.085623	1.729754
35	1	0	3.503829	-1.428194	-1.868963
36	1	0	4.947152	-2.432565	-1.699842
37	1	0	3.452904	-2.880964	-0.847683
38	1	0	4.849414	0.569087	-0.692516
39	1	0	2.344774	1.582640	-1.101974
40	1	0	-0.440341	3.257824	0.042791
41	1	0	1.104447	3.182715	0.908464

1Aa-6

Center Number	Atomic Number	Atomic Type	Coordinates (Angstroms) X	Y	Z
1	8	0	-2.599253	2.819950	-0.122326
2	6	0	-2.283581	1.627973	-0.271149

3	7	0	-3.105414	0.647453	-0.749558
4	6	0	-2.487186	-0.671780	-0.832646
5	6	0	-3.267487	-1.762165	-0.049659
6	6	0	-3.258485	-1.523367	1.467333
7	6	0	-2.774441	-3.171949	-0.408142
8	6	0	-1.054604	-0.391704	-0.320162
9	8	0	-0.187082	-1.272900	-0.270963
10	6	0	-0.982730	1.006329	0.024956
11	6	0	0.088165	1.718524	0.570123
12	6	0	1.420323	1.089430	0.863554
13	6	0	2.312234	0.960953	-0.398096
14	6	0	3.580734	0.146213	-0.121720
15	6	0	3.409868	-1.380597	-0.245373
16	6	0	4.728489	-2.104363	0.012434
17	8	0	2.454502	-1.906125	0.678253
18	8	0	2.724903	2.263462	-0.844033
19	7	0	-0.046293	3.028298	0.809695
20	1	0	-2.405016	-0.991473	-1.881630
21	1	0	1.738370	0.469400	-1.195019
22	1	0	3.078448	-1.604199	-1.272761
23	1	0	-4.052655	0.853389	-1.032787
24	1	0	-4.308171	-1.673757	-0.397945
25	1	0	-3.585702	-0.509330	1.719320
26	1	0	-3.934966	-2.225963	1.965041
27	1	0	-2.257394	-1.676672	1.885245
28	1	0	-2.834785	-3.353981	-1.487471
29	1	0	-1.734693	-3.315223	-0.102170
30	1	0	-3.390634	-3.925870	0.093353
31	1	0	1.273922	0.098210	1.294118
32	1	0	1.966270	1.700480	1.591296
33	1	0	3.974933	0.389704	0.873678

34	1	0	4.329138	0.469056	-0.854435
35	1	0	5.063154	-1.924205	1.040011
36	1	0	4.596060	-3.182187	-0.116760
37	1	0	5.509856	-1.760469	-0.673622
38	1	0	1.556262	-1.775871	0.325933
39	1	0	1.988294	2.695709	-1.296244
40	1	0	-0.950745	3.462680	0.604061
41	1	0	0.709637	3.554238	1.221673

1Aa-7

Center Number	Atomic Number	Atomic Type	Coordinates (Angstroms) X	Y	Z
1	8	0	-2.166568	2.997933	0.073231
2	6	0	-2.195689	1.774357	-0.138985
3	7	0	-3.282516	1.074106	-0.581886
4	6	0	-3.053612	-0.357338	-0.760509
5	6	0	-4.047353	-1.239997	0.040361
6	6	0	-3.878451	-1.093361	1.559716
7	6	0	-3.970353	-2.708775	-0.402646
8	6	0	-1.565816	-0.502401	-0.350073
9	8	0	-0.956260	-1.572156	-0.381820
10	6	0	-1.095853	0.814078	0.031908
11	6	0	0.156795	1.173106	0.517922
12	6	0	1.279842	0.183239	0.675479
13	6	0	2.074294	-0.016904	-0.639568
14	6	0	3.183856	-1.069060	-0.509185
15	6	0	4.370322	-0.706758	0.401884
16	6	0	5.286170	-1.904102	0.635419

17	8	0	5.178864	0.327565	-0.164763
18	8	0	2.686164	1.218351	-1.062872
19	7	0	0.412464	2.455348	0.820919
20	1	0	-3.130654	-0.627328	-1.823789
21	1	0	1.372113	-0.356743	-1.409486
22	1	0	3.989739	-0.371306	1.381821
23	1	0	-4.152006	1.543081	-0.792188
24	1	0	-5.047038	-0.862219	-0.224571
25	1	0	-3.907439	-0.043798	1.870288
26	1	0	-4.683879	-1.619134	2.083156
27	1	0	-2.928899	-1.525780	1.894100
28	1	0	-4.151841	-2.811209	-1.478900
29	1	0	-2.985181	-3.131786	-0.188093
30	1	0	-4.726628	-3.302320	0.122253
31	1	0	0.860988	-0.783829	0.962416
32	1	0	1.960133	0.529804	1.460661
33	1	0	3.579620	-1.275755	-1.511010
34	1	0	2.716533	-1.993480	-0.148991
35	1	0	5.649841	-2.299345	-0.319229
36	1	0	6.152820	-1.597606	1.228312
37	1	0	4.761096	-2.702275	1.170971
38	1	0	4.586514	0.998405	-0.537697
39	1	0	2.017269	1.819526	-1.415260
40	1	0	-0.348584	3.130997	0.712673
41	1	0	1.303322	2.725869	1.208799

1Aa-8

Center	Atomic	Atomic	Coordinates (Angstroms)		
Number	Number	Type	X	Y	Z

1	8	0	-2.122799	2.910788	-0.774318
2	6	0	-2.090824	1.675127	-0.651343
3	7	0	-3.046870	0.810107	-1.104812
4	6	0	-2.767941	-0.599078	-0.840824
5	6	0	-3.901419	-1.309599	-0.054361
6	6	0	-4.068044	-0.761165	1.370384
7	6	0	-3.713077	-2.834047	-0.059909
8	6	0	-1.402851	-0.528937	-0.109454
9	8	0	-0.787239	-1.525490	0.271418
10	6	0	-1.043528	0.870920	-0.005476
11	6	0	0.069645	1.429863	0.613354
12	6	0	1.143367	0.594982	1.258503
13	6	0	2.218977	0.128043	0.249653
14	6	0	3.275596	-0.734752	0.941136
15	6	0	4.440535	-1.214199	0.050012
16	6	0	3.993542	-1.961717	-1.211372
17	8	0	5.333096	-0.144894	-0.271750
18	8	0	2.891460	1.261341	-0.342060
19	7	0	0.238004	2.760945	0.609618
20	1	0	-2.609543	-1.141055	-1.784493
21	1	0	1.725107	-0.451572	-0.538406
22	1	0	5.040721	-1.901288	0.658900
23	1	0	-3.855843	1.146845	-1.607151
24	1	0	-4.824118	-1.084109	-0.611191
25	1	0	-4.175824	0.328431	1.374105
26	1	0	-4.961810	-1.187005	1.838355
27	1	0	-3.208835	-1.024767	1.997070
28	1	0	-3.655221	-3.225486	-1.082254
29	1	0	-2.793207	-3.117213	0.458929
30	1	0	-4.558377	-3.321456	0.437756

31	1	0	0.685400	-0.294280	1.697053
32	1	0	1.635817	1.175430	2.047471
33	1	0	2.762679	-1.610248	1.357756
34	1	0	3.703908	-0.174766	1.782081
35	1	0	3.449857	-1.302774	-1.897130
36	1	0	4.871985	-2.339256	-1.742235
37	1	0	3.345028	-2.810476	-0.963435
38	1	0	4.787253	0.601059	-0.566707
39	1	0	2.312938	1.676989	-0.995395
40	1	0	-0.486486	3.331884	0.166565
41	1	0	1.030624	3.181153	1.070587

1Ab-1

Center Number	Atomic Number	Atomic Type	Coordinates (Angstroms) X	Y	Z
1	8	0	-0.904270	2.413078	-0.697047
2	6	0	-1.413539	1.279589	-0.691742
3	7	0	-2.761208	1.048265	-0.600249
4	6	0	-3.136272	-0.360248	-0.525799
5	6	0	-3.949261	-0.762643	0.727202
6	6	0	-5.360859	-0.156608	0.687739
7	6	0	-3.228925	-0.424112	2.040375
8	6	0	-1.762936	-1.051582	-0.612726
9	8	0	-1.611740	-2.281424	-0.573455
10	6	0	-0.756856	-0.032820	-0.751165
11	6	0	0.603759	-0.293458	-0.928102
12	6	0	1.624977	0.787511	-1.189058
13	6	0	2.300058	1.391778	0.070458

14	6	0	3.087840	0.406146	0.947317
15	6	0	4.356776	-0.192121	0.340771
16	6	0	5.234560	-0.870501	1.391816
17	8	0	3.964689	-1.152383	-0.675352
18	8	0	1.360063	2.016664	0.927205
19	7	0	1.042745	-1.554491	-0.907988
20	1	0	-3.711177	-0.652826	-1.417098
21	1	0	3.011892	2.143159	-0.315498
22	1	0	4.932740	0.606805	-0.151527
23	1	0	-3.402450	1.820852	-0.498267
24	1	0	-4.042221	-1.854292	0.656562
25	1	0	-5.884837	-0.413333	-0.240165
26	1	0	-5.962659	-0.526047	1.524910
27	1	0	-5.333739	0.937472	0.770598
28	1	0	-3.070441	0.654992	2.143658
29	1	0	-3.826354	-0.757176	2.895668
30	1	0	-2.253562	-0.916138	2.105493
31	1	0	1.132607	1.604601	-1.721159
32	1	0	2.408103	0.377164	-1.832181
33	1	0	2.430384	-0.401385	1.291065
34	1	0	3.376050	0.970522	1.841269
35	1	0	4.675433	-1.654613	1.913054
36	1	0	6.119964	-1.326284	0.932072
37	1	0	5.583961	-0.142177	2.130946
38	1	0	4.758918	-1.557034	-1.048052
39	1	0	0.618398	2.371303	0.394794
40	1	0	0.363053	-2.296602	-0.744331
41	1	0	2.039307	-1.738430	-0.967467

1Ab-2

Center Number	Atomic Number	Atomic Type	Coordinates (Angstroms) X	Y	Z
1	8	0	-0.765996	2.648230	-0.425308
2	6	0	-1.359696	1.562667	-0.540947
3	7	0	-2.722393	1.427354	-0.485162
4	6	0	-3.203805	0.048682	-0.507979
5	6	0	-4.040209	-0.329832	0.743241
6	6	0	-3.213606	-0.315938	2.037689
7	6	0	-4.759509	-1.672209	0.542374
8	6	0	-1.885818	-0.733679	-0.680913
9	8	0	-1.817334	-1.968783	-0.774783
10	6	0	-0.805673	0.216491	-0.728479
11	6	0	0.531200	-0.126304	-0.945037
12	6	0	1.628999	0.896672	-1.112032
13	6	0	2.354287	1.322036	0.192124
14	6	0	3.071470	0.199412	0.957821
15	6	0	4.289068	-0.427600	0.279426
16	6	0	5.120044	-1.268680	1.247682
17	8	0	3.821029	-1.251902	-0.820487
18	8	0	1.468348	1.926684	1.118017
19	7	0	0.875550	-1.411875	-1.051104
20	1	0	-3.818989	-0.128065	-1.402004
21	1	0	3.117999	2.053384	-0.127506
22	1	0	4.919987	0.371500	-0.139819
23	1	0	-3.297643	2.218834	-0.234029
24	1	0	-4.810339	0.453083	0.825827
25	1	0	-2.676203	0.628877	2.168437
26	1	0	-3.867654	-0.451248	2.905619
27	1	0	-2.479733	-1.129226	2.045106

28	1	0	-4.039946	-2.487080	0.423570
29	1	0	-5.397006	-1.893289	1.405295
30	1	0	-5.396528	-1.654585	-0.349605
31	1	0	1.196860	1.796664	-1.555273
32	1	0	2.375635	0.494764	-1.802177
33	1	0	2.357689	-0.587540	1.229346
34	1	0	3.405770	0.650225	1.898958
35	1	0	4.507289	-2.057393	1.696694
36	1	0	5.966722	-1.740410	0.734222
37	1	0	5.526480	-0.644327	2.050077
38	1	0	4.580883	-1.674970	-1.241300
39	1	0	0.750774	2.383748	0.632305
40	1	0	0.141796	-2.113555	-0.956659
41	1	0	1.855176	-1.661892	-1.142543

1Ab-3

Center Number	Atomic Number	Atomic Type	Coordinates (Angstroms) X	Y	Z
1	8	0	-0.367953	-1.581636	-0.676539
2	6	0	0.655493	-0.885345	-0.634314
3	7	0	1.924623	-1.405507	-0.586469
4	6	0	2.996784	-0.415608	-0.603087
5	6	0	4.025589	-0.536896	0.545082
6	6	0	4.874780	-1.807865	0.389291
7	6	0	3.377383	-0.462018	1.934862
8	6	0	2.201516	0.900064	-0.581959
9	8	0	2.728902	2.021284	-0.547949
10	6	0	0.797090	0.579899	-0.613998

11	6	0	-0.216508	1.538749	-0.653098
12	6	0	-1.678562	1.195400	-0.687523
13	6	0	-2.242746	0.816881	0.705706
14	6	0	-3.672954	0.274418	0.614331
15	6	0	-3.775642	-1.229175	0.288004
16	6	0	-5.231897	-1.685907	0.260167
17	8	0	-3.205892	-1.559331	-0.979008
18	8	0	-2.272573	1.976108	1.556192
19	7	0	0.115159	2.836584	-0.622595
20	1	0	3.541687	-0.454887	-1.558618
21	1	0	-1.599284	0.050678	1.157460
22	1	0	-3.245313	-1.785678	1.078049
23	1	0	2.062689	-2.398907	-0.701579
24	1	0	4.682692	0.333990	0.423195
25	1	0	5.354575	-1.854945	-0.595130
26	1	0	5.664651	-1.839263	1.147092
27	1	0	4.269559	-2.714209	0.516951
28	1	0	2.671463	-1.285490	2.088931
29	1	0	4.143894	-0.527641	2.714058
30	1	0	2.840058	0.480782	2.079506
31	1	0	-1.843946	0.361888	-1.370935
32	1	0	-2.252004	2.055254	-1.052110
33	1	0	-4.246630	0.848128	-0.125548
34	1	0	-4.139283	0.447644	1.590987
35	1	0	-5.773892	-1.169824	-0.540077
36	1	0	-5.283556	-2.760799	0.065471
37	1	0	-5.732110	-1.475377	1.211561
38	1	0	-2.241565	-1.664620	-0.885064
39	1	0	-1.381061	2.156840	1.881980
40	1	0	1.106064	3.084247	-0.597337
41	1	0	-0.599008	3.547672	-0.667734

1Ab-4

Center Number	Atomic Number	Atomic Type	Coordinates (Angstroms) X	Y	Z
1	8	0	-0.374959	-0.447813	2.308025
2	6	0	-1.142315	-0.004071	1.436056
3	7	0	-2.463555	-0.341789	1.338320
4	6	0	-3.169089	0.253129	0.206634
5	6	0	-3.830127	-0.754626	-0.763647
6	6	0	-5.023088	-1.458028	-0.097717
7	6	0	-2.832347	-1.766999	-1.344314
8	6	0	-2.055549	1.090971	-0.443864
9	8	0	-2.218681	1.780023	-1.460360
10	6	0	-0.855978	0.931624	0.339108
11	6	0	0.329714	1.622092	0.094053
12	6	0	1.540532	1.538452	0.990073
13	6	0	2.520415	0.361003	0.717227
14	6	0	3.212212	0.442031	-0.647466
15	6	0	4.164523	-0.731819	-0.970148
16	6	0	5.246416	-0.965359	0.091265
17	8	0	3.441045	-1.931391	-1.241052
18	8	0	1.877824	-0.902861	0.816320
19	7	0	0.394340	2.435630	-0.968387
20	1	0	-3.943317	0.949553	0.561005
21	1	0	3.284840	0.445039	1.505958
22	1	0	4.661766	-0.485610	-1.916849
23	1	0	-2.868677	-1.000911	1.986625
24	1	0	-4.212026	-0.135262	-1.585711

25	1	0	-5.746332	-0.737587	0.301380
26	1	0	-5.547251	-2.092520	-0.820001
27	1	0	-4.701091	-2.108473	0.725508
28	1	0	-2.018856	-1.272847	-1.884452
29	1	0	-2.388494	-2.387959	-0.558480
30	1	0	-3.339383	-2.434095	-2.049080
31	1	0	1.192794	1.450134	2.022560
32	1	0	2.108313	2.473487	0.908895
33	1	0	3.785731	1.378933	-0.687104
34	1	0	2.457587	0.477894	-1.442189
35	1	0	5.920930	-1.757593	-0.245776
36	1	0	5.839121	-0.058396	0.265348
37	1	0	4.808473	-1.283088	1.043157
38	1	0	2.784884	-2.032285	-0.531067
39	1	0	1.180081	-0.873509	1.507199
40	1	0	-0.430811	2.515800	-1.565891
41	1	0	1.222474	2.978348	-1.157601

1Ab-5

Center Number	Atomic Number	Atomic Type	Coordinates (Angstroms) X	Y	Z
1	8	0	0.423086	-2.126500	-0.759057
2	6	0	1.235512	-1.202541	-0.680855
3	7	0	2.598000	-1.365571	-0.558140
4	6	0	3.370943	-0.127213	-0.568100
5	6	0	4.345781	0.052899	0.619136
6	6	0	5.503315	-0.953947	0.535564
7	6	0	3.641571	-0.014766	1.981739

8	6	0	2.258598	0.933840	-0.617439
9	8	0	2.468804	2.155301	-0.599039
10	6	0	0.991292	0.253484	-0.692146
11	6	0	-0.235234	0.900506	-0.803540
12	6	0	-1.547198	0.166215	-0.874447
13	6	0	-2.097567	-0.196452	0.527339
14	6	0	-3.410044	-0.989295	0.461163
15	6	0	-4.647064	-0.228011	-0.047971
16	6	0	-5.823619	-1.167130	-0.294098
17	8	0	-5.091380	0.752989	0.893125
18	8	0	-2.337549	0.993965	1.307240
19	7	0	-0.273205	2.242272	-0.809313
20	1	0	3.948472	-0.039128	-1.501402
21	1	0	-1.349607	-0.817905	1.032358
22	1	0	-4.402912	0.271328	-1.001197
23	1	0	3.000815	-2.285628	-0.660403
24	1	0	4.757371	1.062765	0.492662
25	1	0	6.019921	-0.896043	-0.429536
26	1	0	6.241345	-0.759887	1.321006
27	1	0	5.150202	-1.983844	0.671794
28	1	0	3.166305	-0.989869	2.134400
29	1	0	4.364562	0.136823	2.790086
30	1	0	2.873108	0.759191	2.077500
31	1	0	-1.396744	-0.765986	-1.423304
32	1	0	-2.279719	0.785717	-1.402732
33	1	0	-3.636223	-1.363225	1.467147
34	1	0	-3.231289	-1.863618	-0.176121
35	1	0	-5.594865	-1.883490	-1.090345
36	1	0	-6.068674	-1.720503	0.618902
37	1	0	-6.704485	-0.588667	-0.587415
38	1	0	-4.309009	1.208248	1.240227

39	1	0	-1.504952	1.343068	1.649519
40	1	0	0.611166	2.750546	-0.759948
41	1	0	-1.149760	2.731617	-0.907256

1Ab-6

Center Number	Atomic Number	Atomic Type	Coordinates (Angstroms) X	Y	Z
1	8	0	-0.538980	-1.696394	-0.580018
2	6	0	0.542799	-1.092771	-0.585446
3	7	0	1.761390	-1.718144	-0.538372
4	6	0	2.920500	-0.832603	-0.568840
5	6	0	3.901706	-1.061855	0.611092
6	6	0	3.282430	-0.714200	1.972581
7	6	0	5.228492	-0.322531	0.382048
8	6	0	2.243297	0.550387	-0.609808
9	8	0	2.858222	1.627217	-0.635858
10	6	0	0.815234	0.353223	-0.626814
11	6	0	-0.110776	1.394299	-0.712699
12	6	0	-1.597238	1.177928	-0.739952
13	6	0	-2.196234	0.919994	0.666205
14	6	0	-3.668434	0.501400	0.591227
15	6	0	-3.902838	-1.001437	0.337466
16	6	0	-5.393769	-1.328534	0.321560
17	8	0	-3.361088	-1.442494	-0.907865
18	8	0	-2.126559	2.117990	1.458690
19	7	0	0.331264	2.658918	-0.737373
20	1	0	3.475942	-0.958580	-1.510098
21	1	0	-1.623748	0.123630	1.159315

22	1	0	-3.426065	-1.563172	1.157376
23	1	0	1.816321	-2.726546	-0.545956
24	1	0	4.113862	-2.141998	0.604162
25	1	0	2.323645	-1.221736	2.120967
26	1	0	3.951795	-1.021011	2.783016
27	1	0	3.121010	0.365378	2.067877
28	1	0	5.074602	0.759734	0.353934
29	1	0	5.934025	-0.552677	1.187545
30	1	0	5.693216	-0.620127	-0.565099
31	1	0	-1.833496	0.329127	-1.382353
32	1	0	-2.092296	2.065325	-1.150581
33	1	0	-4.187236	1.086587	-0.179539
34	1	0	-4.120294	0.762509	1.555070
35	1	0	-5.885767	-0.806275	-0.506499
36	1	0	-5.539914	-2.402880	0.178735
37	1	0	-5.875994	-1.028471	1.258102
38	1	0	-2.409985	-1.626890	-0.801726
39	1	0	-1.223029	2.236782	1.779841
40	1	0	1.340147	2.819964	-0.723772
41	1	0	-0.319094	3.425540	-0.821422

1Ab-7

Center Number	Atomic Number	Atomic Type	Coordinates (Angstroms) X	Y	Z
1	8	0	-0.274742	-1.097441	1.971083
2	6	0	-1.123274	-0.491583	1.313188
3	7	0	-2.426438	-0.905497	1.144684
4	6	0	-3.274172	0.015023	0.392954

5	6	0	-4.028765	-0.609621	-0.804089
6	6	0	-5.107739	-1.590473	-0.319949
7	6	0	-3.087028	-1.269983	-1.821038
8	6	0	-2.266901	1.103517	-0.014716
9	8	0	-2.555851	2.083344	-0.716802
10	6	0	-0.989051	0.771794	0.561054
11	6	0	0.148795	1.562968	0.441058
12	6	0	1.475469	1.189580	1.046028
13	6	0	2.302925	0.252482	0.135304
14	6	0	3.630600	-0.116168	0.798841
15	6	0	4.575673	-1.004545	-0.036798
16	6	0	3.933764	-2.306038	-0.530269
17	8	0	5.165215	-0.276833	-1.117251
18	8	0	2.600177	0.894172	-1.125364
19	7	0	0.091568	2.694793	-0.278342
20	1	0	-4.015612	0.487226	1.055984
21	1	0	1.716736	-0.654590	-0.049356
22	1	0	5.419453	-1.265643	0.613498
23	1	0	-2.774790	-1.686217	1.681665
24	1	0	-4.527124	0.237233	-1.293676
25	1	0	-5.793393	-1.117145	0.392441
26	1	0	-5.703235	-1.956511	-1.162965
27	1	0	-4.663331	-2.467911	0.166424
28	1	0	-2.524372	-2.091705	-1.364757
29	1	0	-3.661041	-1.680224	-2.658482
30	1	0	-2.370596	-0.551822	-2.232967
31	1	0	1.296363	0.667805	1.988594
32	1	0	2.061026	2.096767	1.235977
33	1	0	3.395092	-0.631474	1.737962
34	1	0	4.172551	0.802799	1.055921
35	1	0	3.128437	-2.110154	-1.246616

36	1	0	4.687215	-2.914737	-1.038059
37	1	0	3.519234	-2.887139	0.302060
38	1	0	4.447574	0.203021	-1.560045
39	1	0	1.814391	0.882160	-1.687814
40	1	0	-0.802629	2.956616	-0.696294
41	1	0	0.901963	3.288928	-0.366636

1Ab-8

Center Number	Atomic Number	Atomic Type	Coordinates (Angstroms) X	Y	Z
1	8	0	-0.271694	2.343785	-0.483856
2	6	0	-1.151102	1.480167	-0.519602
3	7	0	-2.499088	1.727149	-0.402839
4	6	0	-3.363487	0.555797	-0.504189
5	6	0	-4.329493	0.396703	0.699999
6	6	0	-3.591566	0.125718	2.019098
7	6	0	-5.402136	-0.665831	0.417569
8	6	0	-2.328868	-0.573128	-0.676104
9	8	0	-2.616403	-1.773584	-0.797409
10	6	0	-1.015026	0.019215	-0.683156
11	6	0	0.162010	-0.697959	-0.872168
12	6	0	1.522794	-0.054571	-0.877540
13	6	0	2.103803	0.109219	0.549066
14	6	0	3.467216	0.813780	0.556401
15	6	0	4.644780	0.038087	-0.060207
16	6	0	5.882838	0.917215	-0.205737
17	8	0	5.024789	-1.080559	0.745970
18	8	0	2.265694	-1.174668	1.187808

19	7	0	0.105869	-2.031404	-1.017556
20	1	0	-3.964718	0.597097	-1.424973
21	1	0	1.402787	0.719024	1.129975
22	1	0	4.360668	-0.320984	-1.064158
23	1	0	-2.838416	2.675728	-0.332739
24	1	0	-4.836582	1.369319	0.792712
25	1	0	-2.809750	0.869836	2.203017
26	1	0	-4.291884	0.161262	2.860163
27	1	0	-3.130073	-0.868221	2.015282
28	1	0	-4.951632	-1.654305	0.291648
29	1	0	-6.116718	-0.712529	1.246348
30	1	0	-5.963073	-0.434229	-0.495347
31	1	0	1.437383	0.941381	-1.317409
32	1	0	2.206691	-0.661675	-1.480209
33	1	0	3.726239	1.048125	1.596122
34	1	0	3.343839	1.768325	0.030907
35	1	0	6.171744	1.334483	0.764962
36	1	0	6.719093	0.320404	-0.581349
37	1	0	5.700102	1.741080	-0.903865
38	1	0	4.214683	-1.517653	1.050162
39	1	0	1.410612	-1.509938	1.485843
40	1	0	-0.813072	-2.477036	-1.021304
41	1	0	0.944952	-2.565993	-1.183720

1Ab-9

Center Number	Atomic Number	Atomic Type	Coordinates (Angstroms) X	Y	Z
1	8	0	-0.112335	-1.326594	1.969502

2	6	0	-1.031965	-0.744972	1.389383
3	7	0	-2.315922	-1.224812	1.275307
4	6	0	-3.251432	-0.354959	0.569775
5	6	0	-3.977710	-1.057309	-0.608152
6	6	0	-3.019187	-1.463299	-1.737068
7	6	0	-5.145401	-0.206461	-1.129510
8	6	0	-2.336985	0.821330	0.176275
9	8	0	-2.712243	1.817010	-0.461155
10	6	0	-1.016385	0.553790	0.687509
11	6	0	0.058747	1.427934	0.563948
12	6	0	1.429262	1.124846	1.107664
13	6	0	2.291050	0.298492	0.124419
14	6	0	3.663938	-0.002793	0.727093
15	6	0	4.642541	-0.774660	-0.182373
16	6	0	4.080790	-2.093355	-0.725408
17	8	0	5.141307	0.049023	-1.239457
18	8	0	2.497459	1.022204	-1.109640
19	7	0	-0.101432	2.581955	-0.102850
20	1	0	-4.013751	0.034982	1.261041
21	1	0	1.766368	-0.639403	-0.088999
22	1	0	5.524403	-1.005153	0.427718
23	1	0	-2.578787	-2.087988	1.728840
24	1	0	-4.399314	-1.978765	-0.178159
25	1	0	-2.173476	-2.045563	-1.356904
26	1	0	-3.542696	-2.077589	-2.477117
27	1	0	-2.627319	-0.581872	-2.257106
28	1	0	-5.856046	0.029849	-0.328972
29	1	0	-4.786259	0.738059	-1.547566
30	1	0	-5.689713	-0.747832	-1.910745
31	1	0	1.322102	0.545264	2.026985
32	1	0	1.953159	2.062899	1.325887

33	1	0	3.498766	-0.581050	1.644309
34	1	0	4.145716	0.939937	1.015984
35	1	0	4.858989	-2.617512	-1.287305
36	1	0	3.737952	-2.745506	0.086654
37	1	0	3.239664	-1.921450	-1.405863
38	1	0	4.376357	0.495499	-1.635460
39	1	0	1.694689	0.982172	-1.646232
40	1	0	0.661458	3.236636	-0.186206
41	1	0	-1.027860	2.799728	-0.473308

1Ba-1

Center Number	Atomic Number	Atomic Type	Coordinates (Angstroms) X	Y	Z
1	8	0	1.149922	2.862130	0.613989
2	6	0	1.543046	1.781806	0.145860
3	7	0	2.849444	1.370273	0.105918
4	6	0	3.045701	0.082316	-0.551511
5	6	0	3.829553	-0.963537	0.273836
6	6	0	3.192980	-1.237560	1.644062
7	6	0	5.306071	-0.561066	0.411162
8	6	0	1.596666	-0.350615	-0.862021
9	8	0	1.329861	-1.464736	-1.338387
10	6	0	0.727223	0.723006	-0.467315
11	6	0	-0.656996	0.826040	-0.634455
12	6	0	-1.467730	-0.208545	-1.378045
13	6	0	-2.042892	-1.361545	-0.515757
14	6	0	-2.990318	-0.943987	0.619006
15	6	0	-4.342380	-0.362775	0.206599

16	6	0	-5.319436	-0.288036	1.379280
17	8	0	-4.118333	0.962799	-0.340911
18	8	0	-1.016040	-2.129838	0.091460
19	7	0	-1.309199	1.891027	-0.167414
20	1	0	3.560412	0.216870	-1.515678
21	1	0	-2.614585	-1.993243	-1.218514
22	1	0	-4.776845	-0.989335	-0.587834
23	1	0	3.592986	2.000961	0.366738
24	1	0	3.776316	-1.883943	-0.321780
25	1	0	2.161138	-1.589561	1.549374
26	1	0	3.759239	-2.009828	2.175536
27	1	0	3.188933	-0.336186	2.266760
28	1	0	5.770255	-0.385811	-0.566507
29	1	0	5.420287	0.351313	1.010409
30	1	0	5.875831	-1.349383	0.914630
31	1	0	-0.832686	-0.657347	-2.146041
32	1	0	-2.302251	0.295565	-1.872703
33	1	0	-2.483173	-0.247264	1.297079
34	1	0	-3.179123	-1.855259	1.197551
35	1	0	-5.539061	-1.289643	1.763195
36	1	0	-4.899583	0.312497	2.193010
37	1	0	-6.270189	0.164430	1.072172
38	1	0	-4.970075	1.352695	-0.577551
39	1	0	-0.226737	-2.127814	-0.485260
40	1	0	-0.763677	2.607295	0.315556
41	1	0	-2.321332	1.928042	-0.233077

1Ba-2

Center Atomic Atomic Coordinates (Angstroms)

Number	Number	Type	X	Y	Z
1	8	0	1.017537	3.043346	0.587111
2	6	0	1.489938	1.977394	0.160510
3	7	0	2.819577	1.655066	0.144187
4	6	0	3.115053	0.339032	-0.412518
5	6	0	3.953478	-0.550927	0.542836
6	6	0	4.498982	-1.791948	-0.179535
7	6	0	3.194778	-0.923040	1.825286
8	6	0	1.701515	-0.193660	-0.744537
9	8	0	1.502832	-1.337423	-1.184526
10	6	0	0.758768	0.842992	-0.423050
11	6	0	-0.623275	0.856445	-0.635731
12	6	0	-1.350640	-0.250334	-1.361653
13	6	0	-1.892404	-1.399726	-0.473566
14	6	0	-2.897144	-0.992236	0.614597
15	6	0	-4.265107	-0.504228	0.138706
16	6	0	-5.281493	-0.437183	1.277940
17	8	0	-4.096494	0.808677	-0.457034
18	8	0	-0.846594	-2.089038	0.193199
19	7	0	-1.350112	1.899520	-0.233572
20	1	0	3.662368	0.437801	-1.362309
21	1	0	-2.406811	-2.086789	-1.168639
22	1	0	-4.639257	-1.185474	-0.641277
23	1	0	3.517736	2.305386	0.474445
24	1	0	4.814936	0.071707	0.831007
25	1	0	5.084209	-1.514062	-1.064180
26	1	0	5.153534	-2.361288	0.489378
27	1	0	3.684682	-2.443376	-0.507414
28	1	0	2.795543	-0.036438	2.328573
29	1	0	2.360411	-1.599218	1.611135

30	1	0	3.864520	-1.432579	2.526258
31	1	0	-0.666684	-0.692159	-2.090665
32	1	0	-2.193897	0.187855	-1.902192
33	1	0	-2.450855	-0.242726	1.279029
34	1	0	-3.054366	-1.889720	1.223379
35	1	0	-5.458117	-1.433283	1.696800
36	1	0	-4.921119	0.218048	2.077861
37	1	0	-6.245337	-0.051265	0.924318
38	1	0	-4.960266	1.140494	-0.735019
39	1	0	-0.037788	-2.054743	-0.354733
40	1	0	-0.862805	2.664008	0.237794
41	1	0	-2.359836	1.875735	-0.333577

1Ba-3

Center Number	Atomic Number	Atomic Type	Coordinates (Angstroms) X	Y	Z
1	8	0	2.242245	2.872225	0.144525
2	6	0	2.228269	1.665231	-0.147466
3	7	0	3.334032	0.865253	-0.250639
4	6	0	3.046739	-0.496340	-0.696058
5	6	0	3.624044	-1.615868	0.198829
6	6	0	3.153467	-1.514008	1.656790
7	6	0	5.157222	-1.659372	0.106500
8	6	0	1.500717	-0.502659	-0.768136
9	8	0	0.850354	-1.507945	-1.053488
10	6	0	1.051377	0.836108	-0.446296
11	6	0	-0.246240	1.335664	-0.461104
12	6	0	-1.441165	0.476049	-0.775524

13	6	0	-1.976731	-0.264835	0.475418
14	6	0	-3.156833	-1.192779	0.156785
15	6	0	-4.470489	-0.512295	-0.267639
16	6	0	-5.490778	-1.526706	-0.774504
17	8	0	-5.084587	0.183025	0.820504
18	8	0	-2.412612	0.671640	1.481239
19	7	0	-0.470964	2.620330	-0.142965
20	1	0	3.417808	-0.649344	-1.720992
21	1	0	-1.162155	-0.877375	0.879108
22	1	0	-4.265103	0.202498	-1.082809
23	1	0	4.258838	1.268051	-0.205501
24	1	0	3.225796	-2.545372	-0.228073
25	1	0	2.062634	-1.565552	1.732823
26	1	0	3.564311	-2.340327	2.246289
27	1	0	3.484648	-0.576507	2.116870
28	1	0	5.496651	-1.752572	-0.931632
29	1	0	5.612012	-0.756575	0.533690
30	1	0	5.553715	-2.513943	0.664899
31	1	0	-1.150225	-0.277739	-1.510619
32	1	0	-2.236778	1.101183	-1.194619
33	1	0	-3.364452	-1.800512	1.045928
34	1	0	-2.829077	-1.878055	-0.634192
35	1	0	-5.132682	-2.024941	-1.681751
36	1	0	-5.688181	-2.285354	-0.009450
37	1	0	-6.433650	-1.021382	-1.002906
38	1	0	-4.388617	0.653552	1.304434
39	1	0	-1.650591	1.100112	1.892246
40	1	0	-1.399108	3.010790	-0.202109
41	1	0	0.341934	3.209579	0.055804

1Ba-4

Center Number	Atomic Number	Atomic Type	Coordinates (Angstroms) X	Y	Z
1	8	0	-2.345155	2.687442	-0.943086
2	6	0	-2.255727	1.605651	-0.339776
3	7	0	-3.270864	0.699928	-0.188689
4	6	0	-2.925049	-0.451527	0.641552
5	6	0	-3.210367	-1.829923	0.004400
6	6	0	-2.500715	-2.019914	-1.343829
7	6	0	-4.721787	-2.078244	-0.118080
8	6	0	-1.425068	-0.209365	0.936804
9	8	0	-0.733190	-1.005865	1.570966
10	6	0	-1.070409	1.060753	0.338150
11	6	0	0.143663	1.735697	0.406218
12	6	0	1.347794	1.166888	1.107979
13	6	0	2.188546	0.245518	0.193211
14	6	0	3.386657	-0.327934	0.951404
15	6	0	4.339924	-1.219010	0.127888
16	6	0	3.645212	-2.387861	-0.579859
17	8	0	5.120488	-0.449537	-0.789803
18	8	0	2.694519	0.977131	-0.944651
19	7	0	0.284166	2.922252	-0.204379
20	1	0	-3.455305	-0.401170	1.604831
21	1	0	1.547533	-0.571068	-0.158261
22	1	0	5.071074	-1.633313	0.832729
23	1	0	-4.210238	0.937231	-0.472951
24	1	0	-2.797658	-2.555773	0.716888
25	1	0	-2.707395	-3.018106	-1.743824
26	1	0	-2.844004	-1.285386	-2.080638

27	1	0	-1.414849	-1.922901	-1.244727
28	1	0	-4.918484	-3.093164	-0.479428
29	1	0	-5.229319	-1.963300	0.846712
30	1	0	-5.186787	-1.386512	-0.831985
31	1	0	1.012730	0.576505	1.963696
32	1	0	1.988202	1.984055	1.460170
33	1	0	2.993571	-0.910444	1.793603
34	1	0	3.975978	0.497430	1.370515
35	1	0	2.953246	-2.035274	-1.352471
36	1	0	4.395172	-3.015565	-1.069298
37	1	0	3.082819	-3.006553	0.129659
38	1	0	4.509491	0.144765	-1.253319
39	1	0	1.978845	1.131621	-1.575776
40	1	0	1.148500	3.437562	-0.135107
41	1	0	-0.535565	3.316354	-0.673323

1Ba-5

Center Number	Atomic Number	Atomic Type	Coordinates (Angstroms) X	Y	Z
1	8	0	-1.926264	2.622878	-1.298546
2	6	0	-1.979195	1.626218	-0.560330
3	7	0	-3.080815	0.837854	-0.375923
4	6	0	-2.890475	-0.234041	0.596614
5	6	0	-3.236851	-1.652608	0.087292
6	6	0	-2.460014	-2.035404	-1.180638
7	6	0	-4.752213	-1.808119	-0.113677
8	6	0	-1.404642	-0.071321	0.975493
9	8	0	-0.828166	-0.863075	1.736744

10	6	0	-0.899915	1.083442	0.282450
11	6	0	0.348877	1.695704	0.399003
12	6	0	1.408130	1.222146	1.363700
13	6	0	2.348003	0.090568	0.861841
14	6	0	3.222671	0.497749	-0.327891
15	6	0	4.154396	-0.611456	-0.867050
16	6	0	5.070731	-1.226796	0.197511
17	8	0	3.420732	-1.619984	-1.560162
18	8	0	1.625842	-1.081744	0.501808
19	7	0	0.613906	2.780234	-0.337006
20	1	0	-3.482840	-0.038567	1.503686
21	1	0	3.000824	-0.133715	1.720129
22	1	0	4.786054	-0.148248	-1.635386
23	1	0	-3.967823	1.089960	-0.786470
24	1	0	-2.926216	-2.322380	0.899456
25	1	0	-2.706326	-3.059869	-1.478471
26	1	0	-2.712542	-1.374101	-2.016639
27	1	0	-1.377627	-1.987615	-1.026282
28	1	0	-5.118960	-1.167416	-0.925712
29	1	0	-5.000619	-2.840142	-0.382576
30	1	0	-5.309369	-1.556690	0.796340
31	1	0	0.908061	0.864337	2.268205
32	1	0	2.034697	2.076112	1.648982
33	1	0	3.840164	1.354232	-0.022428
34	1	0	2.586000	0.823988	-1.158758
35	1	0	4.494517	-1.766490	0.956269
36	1	0	5.748440	-1.942482	-0.276434
37	1	0	5.674503	-0.459561	0.698441
38	1	0	2.668490	-1.863364	-0.995388
39	1	0	0.847528	-1.188487	1.087223
40	1	0	1.489673	3.271153	-0.245432

41	1	0	-0.122447	3.121628	-0.962918

1Ba-6

Center Number	Atomic Number	Atomic Type	Coordinates (Angstroms) X	Y	Z
1	8	0	2.881025	2.619950	-0.125846
2	6	0	2.479721	1.455216	-0.283307
3	7	0	3.277470	0.348178	-0.350584
4	6	0	2.558383	-0.900166	-0.584305
5	6	0	2.891877	-1.998335	0.459810
6	6	0	2.376841	-3.374784	0.013514
7	6	0	2.409610	-1.638120	1.872585
8	6	0	1.084903	-0.427020	-0.607171
9	8	0	0.139899	-1.212232	-0.754390
10	6	0	1.086975	1.004355	-0.433817
11	6	0	0.005157	1.888229	-0.450202
12	6	0	-1.416889	1.433518	-0.616217
13	6	0	-2.030856	0.888638	0.698628
14	6	0	-3.405747	0.252050	0.467752
15	6	0	-3.370483	-1.222709	0.021173
16	6	0	-4.781158	-1.780798	-0.145454
17	8	0	-2.696185	-1.407015	-1.224993
18	8	0	-2.204703	1.958666	1.642060
19	7	0	0.222547	3.197209	-0.276264
20	1	0	2.788593	-1.293413	-1.585716
21	1	0	-1.355397	0.135020	1.124941
22	1	0	-2.850820	-1.802563	0.801723
23	1	0	4.283870	0.431379	-0.334111

24	1	0	3.991533	-2.045324	0.483263
25	1	0	2.770494	-3.647860	-0.972429
26	1	0	2.692959	-4.145258	0.724950
27	1	0	1.285433	-3.385288	-0.047893
28	1	0	1.315669	-1.654284	1.931182
29	1	0	2.789780	-2.363164	2.599773
30	1	0	2.757796	-0.645529	2.176752
31	1	0	-1.467568	0.654834	-1.378312
32	1	0	-2.038219	2.274002	-0.945708
33	1	0	-3.975112	0.840949	-0.263319
34	1	0	-3.944417	0.310087	1.420487
35	1	0	-5.308921	-1.241234	-0.939607
36	1	0	-4.735794	-2.836868	-0.425773
37	1	0	-5.356651	-1.686428	0.781571
38	1	0	-1.734585	-1.431263	-1.074766
39	1	0	-1.347522	2.194276	2.021185
40	1	0	1.191400	3.511581	-0.168274
41	1	0	-0.542917	3.853249	-0.314563

1Ba-7

Center Number	Atomic Number	Atomic Type	Coordinates (Angstroms) X	Y	Z
1	8	0	2.283799	2.998331	0.098632
2	6	0	2.296439	1.783536	-0.160151
3	7	0	3.415899	1.003061	-0.236457
4	6	0	3.163822	-0.388118	-0.603828
5	6	0	3.785492	-1.403072	0.390471
6	6	0	3.766608	-2.828707	-0.181058

7	6	0	3.141378	-1.338447	1.783101
8	6	0	1.617144	-0.422027	-0.708005
9	8	0	0.978213	-1.439770	-0.977434
10	6	0	1.140301	0.920328	-0.441369
11	6	0	-0.165957	1.394837	-0.492378
12	6	0	-1.342198	0.503734	-0.789100
13	6	0	-1.877280	-0.204491	0.480706
14	6	0	-3.037027	-1.164256	0.182263
15	6	0	-4.358219	-0.522819	-0.278093
16	6	0	-5.354817	-1.572330	-0.760001
17	8	0	-4.996026	0.197734	0.779565
18	8	0	-2.340513	0.756958	1.450074
19	7	0	-0.417478	2.686524	-0.226614
20	1	0	3.563637	-0.596945	-1.607250
21	1	0	-1.055935	-0.787769	0.912884
22	1	0	-4.157121	0.167477	-1.115138
23	1	0	4.336296	1.406484	-0.134450
24	1	0	4.838293	-1.097783	0.493767
25	1	0	4.280708	-2.877707	-1.148071
26	1	0	4.274173	-3.517501	0.502852
27	1	0	2.741052	-3.177527	-0.329294
28	1	0	2.110742	-1.709263	1.757181
29	1	0	3.699412	-1.962549	2.488936
30	1	0	3.131919	-0.316314	2.175842
31	1	0	-1.030493	-0.268611	-1.495839
32	1	0	-2.144338	1.100556	-1.236108
33	1	0	-3.243040	-1.745297	1.089458
34	1	0	-2.688710	-1.869686	-0.581770
35	1	0	-5.546602	-2.308009	0.028561
36	1	0	-6.304153	-1.092356	-1.014787
37	1	0	-4.978429	-2.094493	-1.646146

38	1	0	-4.313670	0.697052	1.253969
39	1	0	-1.590676	1.213755	1.852906
40	1	0	0.381672	3.296979	-0.036060
41	1	0	-1.351582	3.057380	-0.311989

1Ba-8

Center Number	Atomic Number	Atomic Type	Coordinates (Angstroms) X	Y	Z
1	8	0	2.425025	2.804135	0.862695
2	6	0	2.352224	1.681906	0.335260
3	7	0	3.369363	0.770990	0.271255
4	6	0	3.041000	-0.457721	-0.447297
5	6	0	3.336232	-1.740353	0.372858
6	6	0	3.271153	-2.995760	-0.509835
7	6	0	2.440998	-1.869002	1.614040
8	6	0	1.551711	-0.231519	-0.815343
9	8	0	0.868426	-1.062008	-1.415555
10	6	0	1.185901	1.084115	-0.330221
11	6	0	-0.022322	1.754407	-0.489791
12	6	0	-1.208878	1.135775	-1.179132
13	6	0	-2.081704	0.293542	-0.219291
14	6	0	-3.260133	-0.334490	-0.964953
15	6	0	-4.243697	-1.152177	-0.101914
16	6	0	-3.578528	-2.262615	0.719178
17	8	0	-5.046793	-0.307876	0.726123
18	8	0	-2.617049	1.115452	0.840865
19	7	0	-0.174392	2.987183	0.018162
20	1	0	3.605703	-0.511850	-1.389948

21	1	0	-1.456152	-0.494334	0.215645
22	1	0	-4.955788	-1.619276	-0.793130
23	1	0	4.291055	0.999049	0.615872
24	1	0	4.375285	-1.628367	0.719403
25	1	0	3.957335	-2.920636	-1.361495
26	1	0	3.553278	-3.881272	0.069884
27	1	0	2.262585	-3.144582	-0.904862
28	1	0	2.468641	-0.961784	2.226660
29	1	0	1.400954	-2.063884	1.329998
30	1	0	2.775125	-2.704295	2.238346
31	1	0	-0.852602	0.478022	-1.974991
32	1	0	-1.833784	1.924986	-1.613422
33	1	0	-2.845679	-0.984584	-1.745191
34	1	0	-3.831234	0.456656	-1.467039
35	1	0	-2.998681	-2.938927	0.079720
36	1	0	-2.908424	-1.851322	1.481980
37	1	0	-4.347124	-2.845809	1.234108
38	1	0	-4.446206	0.318916	1.159482
39	1	0	-1.918966	1.319220	1.477695
40	1	0	0.632291	3.414426	0.480831
41	1	0	-1.033018	3.498171	-0.119843

1Bb-1

Center Number	Atomic Number	Atomic Type	Coordinates (Angstroms) X	Y	Z
1	8	0	0.809303	2.411076	0.481344
2	6	0	1.336549	1.337815	0.145039
3	7	0	2.649955	1.223348	-0.243248

4	6	0	3.002997	-0.091532	-0.776422
5	6	0	4.252508	-0.744230	-0.142709
6	6	0	4.132100	-0.900368	1.379882
7	6	0	5.526808	0.022474	-0.528484
8	6	0	1.706447	-0.886699	-0.538989
9	8	0	1.578414	-2.088499	-0.814471
10	6	0	0.738122	0.002000	0.045792
11	6	0	-0.541713	-0.387370	0.448544
12	6	0	-1.498931	0.535859	1.162904
13	6	0	-2.461084	1.341427	0.249812
14	6	0	-3.411269	0.509371	-0.625393
15	6	0	-4.488884	-0.295645	0.100248
16	6	0	-5.574482	-0.796373	-0.851626
17	8	0	-3.847930	-1.419555	0.759450
18	8	0	-1.759523	2.197128	-0.635025
19	7	0	-0.948001	-1.640818	0.234430
20	1	0	3.159172	-0.031845	-1.864357
21	1	0	-3.078849	1.942324	0.940890
22	1	0	-4.949805	0.335054	0.876210
23	1	0	3.169511	2.064643	-0.449679
24	1	0	4.302450	-1.745617	-0.590015
25	1	0	5.026993	-1.386484	1.782825
26	1	0	4.028376	0.073890	1.869779
27	1	0	3.269045	-1.514650	1.655568
28	1	0	5.627748	0.116969	-1.616101
29	1	0	5.532596	1.031111	-0.096597
30	1	0	6.417262	-0.494254	-0.154764
31	1	0	-0.915982	1.256145	1.741290
32	1	0	-2.102071	-0.057381	1.855462
33	1	0	-2.835836	-0.155842	-1.280293
34	1	0	-3.913509	1.228533	-1.282131

35	1	0	-6.103720	0.045348	-1.310136
36	1	0	-5.136816	-1.407706	-1.647771
37	1	0	-6.316720	-1.403652	-0.319331
38	1	0	-4.523151	-1.953151	1.198545
39	1	0	-0.919515	2.480911	-0.218444
40	1	0	-1.900543	-1.905005	0.466202
41	1	0	-0.307444	-2.278635	-0.237396

1Bb-2

Center Number	Atomic Number	Atomic Type	Coordinates (Angstroms) X	Y	Z
1	8	0	0.663693	2.616300	0.327007
2	6	0	1.269631	1.563855	0.064798
3	7	0	2.585699	1.517346	-0.320993
4	6	0	3.049769	0.197371	-0.743471
5	6	0	4.357560	-0.247134	-0.038597
6	6	0	4.958539	-1.490081	-0.712047
7	6	0	4.174085	-0.448808	1.472501
8	6	0	1.809965	-0.673209	-0.453055
9	8	0	1.757810	-1.897408	-0.646457
10	6	0	0.775314	0.182655	0.065332
11	6	0	-0.477052	-0.268955	0.489736
12	6	0	-1.506359	0.632026	1.128733
13	6	0	-2.514902	1.296087	0.153879
14	6	0	-3.391854	0.334793	-0.663494
15	6	0	-4.417186	-0.490995	0.112763
16	6	0	-5.452750	-1.137707	-0.806321
17	8	0	-3.704824	-1.513907	0.857546

18	8	0	-1.868572	2.134939	-0.787383
19	7	0	-0.790204	-1.560774	0.367292
20	1	0	3.219578	0.183046	-1.830599
21	1	0	-3.182574	1.898168	0.795773
22	1	0	-4.931381	0.159850	0.836884
23	1	0	3.061924	2.375809	-0.559947
24	1	0	5.061074	0.587141	-0.182685
25	1	0	5.916187	-1.747659	-0.246657
26	1	0	4.286129	-2.347683	-0.622121
27	1	0	5.140810	-1.317366	-1.779326
28	1	0	3.720236	0.430115	1.941493
29	1	0	3.539526	-1.317527	1.680206
30	1	0	5.142980	-0.623272	1.952442
31	1	0	-0.983746	1.433298	1.655907
32	1	0	-2.072476	0.048227	1.859537
33	1	0	-2.761772	-0.332951	-1.263155
34	1	0	-3.936784	0.965101	-1.375163
35	1	0	-6.035527	-0.372492	-1.329433
36	1	0	-4.963058	-1.772667	-1.552084
37	1	0	-6.155766	-1.756305	-0.235232
38	1	0	-4.344917	-2.061137	1.331106
39	1	0	-1.056987	2.510437	-0.387446
40	1	0	-1.723961	-1.874999	0.612652
41	1	0	-0.101373	-2.181936	-0.056473

1Bb-3

Center Number	Atomic Number	Atomic Type	Coordinates (Angstroms) X	Y	Z

1	8	0	0.321330	-1.599741	-0.190415
2	6	0	-0.667671	-0.857373	-0.261640
3	7	0	-1.911273	-1.286562	-0.652276
4	6	0	-2.918775	-0.236091	-0.760764
5	6	0	-4.217407	-0.481111	0.043256
6	6	0	-3.957599	-0.694876	1.541251
7	6	0	-5.023080	-1.642735	-0.558489
8	6	0	-2.130284	0.997258	-0.291045
9	8	0	-2.620477	2.131988	-0.198680
10	6	0	-0.782733	0.585073	0.009313
11	6	0	0.201540	1.445882	0.497422
12	6	0	1.603812	1.006569	0.808979
13	6	0	2.500810	0.916842	-0.452261
14	6	0	3.856713	0.273058	-0.143162
15	6	0	3.862268	-1.268770	-0.167966
16	6	0	5.259884	-1.816429	0.109232
17	8	0	2.985260	-1.839995	0.803341
18	8	0	2.753593	2.234347	-0.970715
19	7	0	-0.093414	2.742189	0.665037
20	1	0	-3.192395	-0.073568	-1.814391
21	1	0	1.987316	0.317638	-1.215318
22	1	0	3.547805	-1.593787	-1.173241
23	1	0	-2.028295	-2.226790	-1.000850
24	1	0	-4.798957	0.441979	-0.078937
25	1	0	-4.904394	-0.838834	2.072164
26	1	0	-3.337850	-1.581353	1.714430
27	1	0	-3.454076	0.166314	1.991925
28	1	0	-5.227439	-1.484007	-1.623730
29	1	0	-4.492712	-2.597326	-0.451502
30	1	0	-5.985250	-1.750278	-0.046835
31	1	0	1.585072	0.031137	1.295804

32	1	0	2.071690	1.722006	1.494851
33	1	0	4.226926	0.623400	0.829409
34	1	0	4.557842	0.632288	-0.905185
35	1	0	5.988213	-1.428741	-0.611131
36	1	0	5.582693	-1.533802	1.117407
37	1	0	5.252762	-2.908392	0.049738
38	1	0	2.076445	-1.856077	0.451631
39	1	0	1.980869	2.537057	-1.465462
40	1	0	0.597038	3.379846	1.031686
41	1	0	-1.043016	3.055333	0.455205

1Bb-4

Center Number	Atomic Number	Atomic Type	Coordinates (Angstroms) X	Y	Z
1	8	0	-0.423837	-2.123392	-0.194021
2	6	0	-1.219466	-1.183475	-0.254028
3	7	0	-2.553050	-1.304734	-0.577849
4	6	0	-3.279044	-0.042497	-0.683895
5	6	0	-4.566040	0.052900	0.169388
6	6	0	-4.307136	-0.187673	1.663449
7	6	0	-5.651944	-0.890285	-0.370718
8	6	0	-2.199041	0.973724	-0.276091
9	8	0	-2.396590	2.194627	-0.192538
10	6	0	-0.978157	0.253922	-0.019869
11	6	0	0.203799	0.850729	0.406964
12	6	0	1.469335	0.076839	0.661865
13	6	0	2.305564	-0.128767	-0.625924
14	6	0	3.573819	-0.957911	-0.381557

15	6	0	4.675265	-0.294804	0.464656
16	6	0	5.771892	-1.287207	0.837974
17	8	0	5.310150	0.777883	-0.236619
18	8	0	2.705564	1.140888	-1.184081
19	7	0	0.248090	2.182864	0.565576
20	1	0	-3.547424	0.162769	-1.731798
21	1	0	1.681884	-0.665197	-1.349496
22	1	0	4.234869	0.095417	1.398140
23	1	0	-2.905431	-2.189715	-0.912356
24	1	0	-4.911640	1.086897	0.040003
25	1	0	-5.239248	-0.089078	2.229670
26	1	0	-3.911283	-1.193660	1.839907
27	1	0	-3.594452	0.535751	2.072346
28	1	0	-5.853511	-0.708312	-1.432811
29	1	0	-5.363652	-1.942499	-0.253820
30	1	0	-6.590837	-0.750831	0.175461
31	1	0	1.205507	-0.910380	1.046982
32	1	0	2.073215	0.607850	1.405466
33	1	0	4.008009	-1.216098	-1.355118
34	1	0	3.263759	-1.896596	0.093063
35	1	0	5.378729	-2.085765	1.476135
36	1	0	6.204346	-1.735908	-0.062856
37	1	0	6.571167	-0.770791	1.377467
38	1	0	4.619768	1.291080	-0.683652
39	1	0	1.969686	1.542334	-1.663122
40	1	0	1.090401	2.634789	0.887595
41	1	0	-0.604503	2.718020	0.393482

1Bb-5

Center Number	Atomic Number	Atomic Type	Coordinates (Angstroms) X	Y	Z
1	8	0	0.486593	-1.682269	-0.267263
2	6	0	-0.558895	-1.021080	-0.334533
3	7	0	-1.750516	-1.528345	-0.782924
4	6	0	-2.856189	-0.577499	-0.831890
5	6	0	-4.106532	-1.045368	-0.040158
6	6	0	-5.324345	-0.163035	-0.352785
7	6	0	-3.847359	-1.146947	1.469931
8	6	0	-2.179768	0.700258	-0.300671
9	8	0	-2.755192	1.791405	-0.171465
10	6	0	-0.804506	0.390842	0.001737
11	6	0	0.097653	1.309981	0.539979
12	6	0	1.530207	0.978594	0.848129
13	6	0	2.441492	1.022284	-0.405266
14	6	0	3.845535	0.484518	-0.108995
15	6	0	3.984463	-1.048615	-0.198784
16	6	0	5.422659	-1.485207	0.067934
17	8	0	3.154818	-1.734354	0.739448
18	8	0	2.583995	2.378159	-0.863086
19	7	0	-0.307877	2.567613	0.762058
20	1	0	-3.156555	-0.390551	-1.873539
21	1	0	1.987718	0.415674	-1.199735
22	1	0	3.704935	-1.356228	-1.219738
23	1	0	-1.803582	-2.478849	-1.120109
24	1	0	-4.321033	-2.058378	-0.413710
25	1	0	-6.212832	-0.550501	0.157105
26	1	0	-5.157535	0.866680	-0.024686
27	1	0	-5.537746	-0.142299	-1.427878
28	1	0	-2.963049	-1.754359	1.688008

29	1	0	-3.702550	-0.155514	1.913319
30	1	0	-4.703509	-1.611143	1.970457
31	1	0	1.591561	-0.015342	1.291923
32	1	0	1.929250	1.700444	1.569866
33	1	0	4.177200	0.823217	0.881449
34	1	0	4.518416	0.935155	-0.847565
35	1	0	6.118675	-1.006103	-0.628998
36	1	0	5.714413	-1.218863	1.089915
37	1	0	5.510165	-2.570226	-0.037206
38	1	0	2.252648	-1.813212	0.379186
39	1	0	1.789137	2.637282	-1.347452
40	1	0	0.322910	3.243740	1.165459
41	1	0	-1.278089	2.808617	0.551142

1Bb-6

Center Number	Atomic Number	Atomic Type	Coordinates (Angstroms) X	Y	Z
1	8	0	-0.241246	-1.856288	0.831741
2	6	0	-1.053123	-1.076540	0.328867
3	7	0	-2.294278	-1.437091	-0.148206
4	6	0	-3.043542	-0.362111	-0.792252
5	6	0	-4.472532	-0.133149	-0.245523
6	6	0	-4.492840	0.153294	1.262683
7	6	0	-5.391429	-1.310742	-0.605244
8	6	0	-2.108123	0.840256	-0.580613
9	8	0	-2.373033	1.994069	-0.948068
10	6	0	-0.926545	0.376356	0.099866
11	6	0	0.125293	1.200742	0.485608

12	6	0	1.354688	0.689728	1.187651
13	6	0	2.435664	0.183778	0.203489
14	6	0	3.655708	-0.341613	0.961277
15	6	0	4.832250	-0.824875	0.087912
16	6	0	4.444616	-1.885248	-0.948818
17	8	0	5.519035	0.269841	-0.524102
18	8	0	2.878195	1.251364	-0.664909
19	7	0	0.080055	2.509199	0.188928
20	1	0	-3.116396	-0.537230	-1.876831
21	1	0	2.000929	-0.619487	-0.401485
22	1	0	5.569334	-1.267314	0.769072
23	1	0	-2.535710	-2.414918	-0.217689
24	1	0	-4.833839	0.759793	-0.772261
25	1	0	-4.093166	-0.692764	1.832157
26	1	0	-3.904589	1.041730	1.513859
27	1	0	-5.519350	0.330726	1.600265
28	1	0	-6.422646	-1.102570	-0.301062
29	1	0	-5.394662	-1.505988	-1.683968
30	1	0	-5.083263	-2.230896	-0.093125
31	1	0	1.071207	-0.145840	1.830945
32	1	0	1.787650	1.490423	1.798791
33	1	0	3.316238	-1.171871	1.592454
34	1	0	4.032334	0.444544	1.627941
35	1	0	3.956920	-2.745956	-0.475969
36	1	0	3.764228	-1.477994	-1.704639
37	1	0	5.342450	-2.235053	-1.466101
38	1	0	4.842671	0.846293	-0.913400
39	1	0	2.215683	1.405266	-1.351459
40	1	0	0.827229	3.125704	0.470387
41	1	0	-0.747832	2.867499	-0.289662

1Bb-7

Center Number	Atomic Number	Atomic Type	Coordinates (Angstroms) X	Y	Z
1	8	0	-0.280909	-2.264442	-0.285749
2	6	0	-1.135437	-1.376797	-0.335923
3	7	0	-2.446025	-1.568742	-0.706523
4	6	0	-3.271594	-0.365777	-0.740606
5	6	0	-4.559409	-0.477781	0.118815
6	6	0	-5.525613	0.680459	-0.171476
7	6	0	-4.260178	-0.609379	1.619147
8	6	0	-2.265901	0.707797	-0.281889
9	8	0	-2.535487	1.912218	-0.158266
10	6	0	-1.000336	0.062811	-0.038451
11	6	0	0.134255	0.721602	0.424523
12	6	0	1.448729	0.026457	0.658732
13	6	0	2.304062	-0.069007	-0.629247
14	6	0	3.622800	-0.822825	-0.409361
15	6	0	4.670995	-0.128320	0.478125
16	6	0	5.829722	-1.061329	0.815285
17	8	0	5.236544	1.017122	-0.165133
18	8	0	2.623054	1.246481	-1.130587
19	7	0	0.086744	2.045877	0.638465
20	1	0	-3.567000	-0.128094	-1.773686
21	1	0	1.721621	-0.614031	-1.380313
22	1	0	4.198712	0.186204	1.424491
23	1	0	-2.758053	-2.476905	-1.019166
24	1	0	-5.043978	-1.409783	-0.210239
25	1	0	-6.457612	0.545327	0.387808

26	1	0	-5.085536	1.639823	0.114587
27	1	0	-5.779167	0.732056	-1.236802
28	1	0	-3.550017	-1.418314	1.818671
29	1	0	-3.845312	0.321303	2.022113
30	1	0	-5.180263	-0.827527	2.171415
31	1	0	1.251431	-0.991216	1.002028
32	1	0	2.010533	0.567447	1.427658
33	1	0	4.080452	-1.006413	-1.389093
34	1	0	3.372108	-1.800829	0.018635
35	1	0	5.487142	-1.914169	1.410960
36	1	0	6.297887	-1.436254	-0.101282
37	1	0	6.588367	-0.518646	1.386725
38	1	0	4.516571	1.502084	-0.596767
39	1	0	1.863236	1.621191	-1.593697
40	1	0	0.894092	2.539560	0.987609
41	1	0	-0.798433	2.528753	0.476572

Table S7. Detailed analysis of the gene NRPS/PKS hybdrid gene cluster of *C. sphaerospermum* SW67 and *C. sphaerospermum* UM843 including top BLAST hits from the UniProtKB sequence database sorted after BLAST score.

Identifier UM 843	Identifier SW67	Size (aa)	Gene name	Annotation	Database	Closest homolog (origin)	Annotation	Identity [%]/ score	Acc No.
Ctg24_orf36	Ctg15_orf36	2167	*clsA*	N-acetyltransferase	UniProt	*Pseudocercospora musae* 113226<br>*Penicillium brasilianum* ATCC 12072<br>*Aspergillus clavatus* ATCC 1007	Uncharacterized protein<br>Putative siderophore esterase<br>Putative siderophore esterase	72/ 1088<br>66/1014<br>67/1012	A0A139GV05<br>A0A1S9RZT7<br>A1C4M6
Ctg24_orf37	Ctg15_orf37	3465	*clsB*	ABC transporter	UniProt	*Aspergillus novofumigatus* IBT 16806<br>*Penicillium brasilianum*<br><br>*Aspergillus lentulus*	Putative ABC multidrug transporter<br>Putative Multidrug/pheromone exporter, ABC superfamily<br>Leptomycin B resistance protein pmd1	73/4191<br>74/4187<br><br>73/4166	A0A2I1C1G8<br>A0A0F7VIH0<br><br>A0A0S7DHX8
					MIBIG	*Amycolatopsis coloradensis* ATCC:53629<br>*Streptomyces glaucescens* ATCC:19761<br>*Sphingomonas sp. ATCC 53159*	ABC_transporter<br>ABC_transporter_ATP-binding_protein<br>ABC_transporter	42/183<br>42/170<br>41/182	OLZ52458.1<br>WP_078957969.1<br>ABW74893.1
Ctg24_orf38	Ctg15_orf38	989	*clsC*	Uncharacterized protein	UniProt	*Rachicladosporium antarcticum*	Uncharacterized proteins	77/1337<br>76/1306<br>76/1299	A0A1V8T847<br>A0A1V8UD24<br>A0A1V8TCT0
					MIBiG	*Purpureocillium lilacinum* ATCC:10114<br>*Aspergillus niger* ATCC 1015<br>*Alternaria alternate* ATCC 66981	Phenylacetyl-ligase_protein<br>Hypothetical_protein<br>Acyl-CoA Synthetase	35/194<br>34/271<br>33/231	AQ83766.1<br>EHA28233.1<br>BAH83502.1
Ctg24_orf39	Ctg15_orf39	1672	*clsD*	AMP-dependent synthetase and ligase	UniProt	*Rachicladosporium* sp. CCFEE 5018<br>*Cercospora beticola*<br>*Sphaerulina musiva strain* SO2202	Uncharacterized protein<br>Putative 4-coumarate--CoA ligase 1<br>Acetyl-CoA synthetase-like protein	78/2338<br>74/2212<br>73/2206	A0A1V8UD69<br>A0A2G5HD40<br>M3CCD9
					MIBiG	*Purpureocillium lilacinum* ATCC10114<br><br>*Aspergillus niger* ATCC 1015<br><br>*Bacillus subtilis* subsp. spizizenii ATCC 6633	Phenylacetyl- ligase protein (leucinostatin A)<br>AMP-dependent synthetase and ligase (azanigerone A)<br>Acyl-CoA dehydrogenase (mycosubtilin)	35/194<br><br>34/271<br><br>31/217	OAQ83766.1<br><br>EHA28233.1<br><br>AAF08800.1
Ctg24_orf40	Ctg15_orf40	1514	*clsE*	Uncharacterized protein	UniProt	*Rachicladosporium* sp. CCFEE 5018	Uncharacterized proteins	64.8/1608<br>64.3/1599<br>64.2/1579	A0A1V8UP25<br>A0A1V8URL1<br>A0A1V8T8L7

Ctg24_orf41	Ctg15_orf41	3694	*clsF*	Gamma-glutamyltranspeptidase	UniProt	*Quercus suber*<br>*Exophiala* dermatitidis strain ATCC 34100<br>*Macrophomina phaseolina* strain MS6	Gamma-glutamyltransferase/<br>Gamma-glutamyltranspeptidase<br>Gamma-glutamyltranspeptidase	66/1974<br>69.4/1964<br>67.0/1953	A0A2P4I3Z8<br>H6BKZ8<br>K2RPI9
					MIBiG	*Aspergillus flavus* NRRL3357<br><br>*Amycolatopsis orientalis* subsp.<br><br>*Vinearia*<br>*Streptomyces sp.* ICBB 8177	Gamma_glutamyl_transpeptidase (ustiloxin B)<br>Gamma-Glutamyltransferase (BE-7585A)<br><br>Gamma-glutamyltransferase-like_hydrolase (limazepine C)	51/531<br><br>29/172<br><br>24/96	EED49422.1<br><br>AVV48310.1<br><br>AOC89013.1
Ctg24_orf42	Ctg15_orf42	2149	*clsG*	Drug resistance transporter	UniProt	*Beauveria bassiana* strain ARSEF 2860<br>*Beauveria bassiana*<br>*Beauveria bassiana* D1-5	MFS drug efflux transporter<br>Efflux pump roqT<br>Putative HC-toxin efflux carrier TOXA	71.1/1840<br>71.1/1814<br>72.2/1806	J4UG41<br>A0A2N6P2Z4<br>A0A0A2VQU0
					MIBIG	*Alternaria brassicicola* ATCC 96836<br>*Penicillium expansum* ATCC 24692	MFS_transporter<br>MFS_transporter	36/314<br>36/284	ACZ57546.1<br>AIG62136.1
Ctg24_orf43	Ctg15_orf43	2109	*clsH*	Uncharacterized protein	UniProt	*Beauveria bassiana*	Uncharacterized protein	61.1/1038<br>61.2/1008<br>60.5/990	A0A0A2VAT7<br>A0A2S7YHY1<br>A0A2N6P2Z6
Ctg24_orf44	Ctg15_orf44	11262	*clsI*	NRPS/PKS	UniProt	*Elaphomyces granulatus*<br>*Aspergillus carbonarius* strain ITEM 5010<br><br>*Talaromyces stipitatus* strain ATCC 10500	Polyketide synthase, putative<br>Hybrid_NRPS/PKS_enzyme, (cytochalasin E)<br>Hybrid_PKS-NRPS (aspyridone A)	41.1/5469<br>41.5/5357<br><br>39.9/5284	A0A232LUQ5<br>A0A1R3RKP6<br><br>B8M2A8
					MIBiG	*Aspergillus clavatus* NRRL 1<br>*Aspergillus nidulans* FGSC A4<br>*Phoma betae* 137527	Polyketide_synthase (betaenone C)	41/682<br>41/651<br>40/677	EAW09117.1<br>CBF80487.1<br>BAQ25466.1
Ctg24_orf45	Ctg15_orf45	1102	*clsJ*	Uncharacterized protein	UniProt	*Verticillium longisporum*<br>*Aspergillus steynii* IBT 23096<br>*Aspergillus campestris* IBT 28561	Uncharacterized proteins	40.2/359<br>39.9/353<br>37.4/333	A0A0G4MDE0<br>A0A2I2FWU0<br>A0A2I1D470
Ctg24_orf46	Ctg15_orf46	2578	*clsK*	Cytochrome P450	UniProt	*Aspergillus steynii* IBT 23096<br>*Meliniomyces variabilis*<br>*Penicillium occitanis*	Cytochrome P450<br>Cytochrome P450<br>Cytochrome P450	41.9/379<br>40.4/310<br>34.7/309	A0A2I2FWM4<br>A0A2J6S9G9<br>A0A2H3IRR7

					MIBiG	*Aspergillus clavatus* NRRL 1<br>*Cordana terrestris* 1293529<br>*Sordaria araneosa* 573841	Cytochrome_P450 (cytochalasin E)<br>Cytochrome_P450 (sespendole)<br>P450_monooxygenase (sordarin)	35/86<br>32/92<br>32/86	EAW09122.1<br>BBD84646.1<br>CBX55165.1
Ctg24_ orf47	Ctg15_orf47	1810	*clsL*	Sugar transport protein	UniProt	*Cercospora beticola*<br>*Cercospora zeina*<br>*Aureobasidium pullulans* EXF-150	Lactose permease<br>Lactose permease<br>MFS sugar Transporter-like protein	74.6/2159<br>74.3/2156<br>72.6/2021	A0A2G5HEM8<br>A0A2I0RTH1<br>A0A074XF51
					MIBiG	*Sordaria araneosa* 573841<br>*Aspergillus flavus* NRRL3357<br>*Streptomyces nanchangensis* 204925	Putative_MFS_sugar_transporter<br>MFS_glucose_transporter,_putative<br>Sugar_transporter	39/349<br>25/134<br>25/97	BAV32173.1<br>EED57519.1<br>ADC45555.1

a) Homologs of biosynthetic gene clusters encoding for characterized compounds were considered; b) percent identity and positive alignments were determined using BLASTp of the UniProt server, following default parameters and using the UniProtKB database. Percent of positives is the proportion of the query sequence that aligns to each homolog residues for which the alignment scores have positive values.

Table S8. List of NR-PKSs used for alignment of unknown ClsI and CluI domains.

**Accession No.**	**Organism**	**Product**
B1GVX7	*Botryotinia fuckeliana* B05.10	Botcinin acid
Q9Y8A5	*Aspergillus terreus* ATCC 1012	Lovastatin nonaketide
S0EET5	*Gibberella fujikuroi* CBS 195.34	Fujikurin A

Table S9. List of NR-PKSs related to known polyketides used for phylogenetic analysis of fungal product-release enzymes.

Group	Accession No.	Organism	Products	Catalytic mechanisms
I	AGC95321	*Aspergillus terreus* AtCURS2	10,11-dehydrocurvularin	TE (macrolactone closure)
I	ACM42403	*Chaetomium chiversii* RADS2	radicicol	TE (macrolactone closure)
I	ACD39762	*Hypomyces subiculosus* Hpm3	hypothemycin	TE (macrolactone closure)
II	AAD31436	*Exophiala dermatitidis* PKS1	T4HN	TE/CLC (second-ring cyclization)
II	AAN59953	*Glarea lozoyensis* PKS1	T4HN	TE/CLC (second-ring cyclization)
II	ABD47522	*Ophiostoma piceae* PKSA	T4HN	TE/CLC (second-ring cyclization)
III	AAC39471	*Aspergillus fumigatus* Alb1	naphthopyrones	TE/CLC (second-ring cyclization)
III	EDP55264	*Aspergillus fumigatus* PksP	T4HN	TE/CLC (the second-ring cyclization)
III	CAB92399	*Fusarium fujikuroi* PKS4	bikaverin	TE/CLC (second-ring cyclization)
IV	AAS90093	*Aspergillus flavus* PksA	aflatoxin	TE/CLC (second-ring cyclization)
IV	Q12397	*Aspergillus nidulans* StcA	sterigmatocystin	TE/CLC (second-ring cyclization)
IV	AAT69682	*Cercospora nicotianae* CTB1	cercosporin	TE (pyrone formation)
V	XP_746434	*Aspergillus fumigatus* EncB	endocrocin	MβL-TE
V	CBF70385	*Aspergillus nidulans* AptB	asperthecin	MβL-TE
V	ADI24932	*Penicillium aethiopicum* VrtG	viridicatumtoxin	MβL-TE
VI	XP_681652	*Aspergillus nidulans* AusA	3,5-dimethylorsellinic acid, austinol, dehydroaustinol	TE-like (hydrolysis)
VI	XP_664052	*Aspergillus nidulans* PkbA	3-methylorsellinic acid, cichorine	TE-like (hydrolysis)
VI	ADY00130	*Penicillium brevicompactum* MpaC	5-methylorsellinic acid, mycophenolic acid	TE-like (hydrolysis)
VII	XP_658638	*Aspergillus nidulans* AfoE	asperfuranone	R
VII	AGN71604	*Monascus pilosus* PKS5	rubropunctatin	R
VII	ACLA_078660	*Aspergillus clavatus* NRRL 1	cytochalasin E	R
VII	A0A0C6E0I7	*Phoma betae* 137527	betaenone	R
VII	clsI	*Cladosporium sphaerospermum* SW67	cladosins, cladosporiumins, and cladosporicins	R
VII	cluI	*Cladosporium sphaerospermum* UM843	Unknown	R
VII	EHA28237	*Aspergillus niger* ATCC 1015	azaphilones	R
VII	ANIA08412	*Emericella nidulans* FGSC A4	aspyridones	R
VII	S0EEY3	*Gibberella fujikuroi* CBS 195.34	fusarin C	R
VII	A0JJU1	*Beauveria bassiana* ARSEF 1564	tenellin	R

References

1. Liang, X.; Huang, Z.H.; Ma, X.; Qi, S.H. Unstable Tetramic Acid Derivatives from the Deep-Sea-Derived Fungus Cladosporium sphaerospermum EIODSF 008. *Marine drugs*, **2018**, *16*, 448.

2. Huang, Z.H.; Nong, X.H.; Liang, X.; Qi, S.H. New tetramic acid derivatives from the deep-sea-derived fungus Cladosporium sp. SCSIO z0025. *Tetrahedron*, **2018**, *74*, 2620-2626.

3. Zhu, G.; Kong, F.; Wang, Y.; Fu, P.; Zhu, W. Cladodionen, a cytotoxic hybrid polyketide from the marine-derived Cladosporium sp. OUCMDZ-1635. *Marine drugs*, **2018**, *16*, 71.

4. Wu, G.; Sun, X.; Yu, G.; Wang, W.; Zhu, T.; Gu, Q.; Li, D. Cladosins A–E, hybrid polyketides from a deep-sea-derived fungus, *Cladosporium sphaerospermum*. *Journal of Natural Products*, **2014**, *77*, 270-275.

5. Yu, G.H.; Wu, G.W.; Zhu, T.J.; Gu, Q.Q.; Li, D.H. Cladosins F and G, two new hybrid polyketides from the deep-sea-derived Cladosporium sphaerospermum 2005-01-E3. *Journal of Asian Natural Products Research*, **2015**, *17*, 120-124.

6. Rischera, M.; Lee, S.R.; Eom, H.J.; Park, H.B.; Vollmers, J.; Kaster, A.K.; Shin, Y.H.; Oh, D.C.; Kim, K.H.; Beemelmanns, C. Spirocyclic cladosporicin A and cladosporiumins I and J from a *Hydractinia*-associated *Cladosporium sphaerospermum* SW67. *Organic Chemistry Frontiers*, **2019**, *6*, 1084–1093.
